# Frequency of Human Brucellosis Complications in West Asia: A Systematic Review and Meta‐Analysis

**DOI:** 10.1002/mbo3.70388

**Published:** 2026-09-07

**Authors:** Aliasghar Fakhri‐Demeshghieh, Elnaz Rostamzaman, Fatemeh Sayareh, Amir Nikoukar, Faranak Farahani, Farniya Bakhtiary nia, Helia Sepahvand, Maryam Khaleghi, Mohammadsaeed Azamian, Mohammad Reza Shirzadi, Hesameddin Akbarein, Vahid Rahmanian

**Affiliations:** ^1^ University of Tehran Tehran Iran; ^2^ Department of Epidemiology and Biostatistics, School of Public Health Tehran University of Medical Sciences Tehran Iran; ^3^ Department of Microbiology and Clinical Immunology, Faculty of Veterinary Medicine University of Warmia and Mazury in Olsztyn Olsztyn Poland; ^4^ Department of Avian Diseases, Faculty of Veterinary Medicine University of Tehran Tehran Iran; ^5^ Faculty of Veterinary Medicine University of Tehran Tehran Iran; ^6^ Department of Food Hygiene and Quality Control, Division of Epidemiology & Zoonoses, Faculty of Veterinary Medicine University of Tehran Tehran Iran; ^7^ Center for Communicable Diseases Management Ministry of Health and Medical Education Tehran Iran; ^8^ Department of Public Health Torbat Jam University of Medical Sciences Torbat Jam Iran

**Keywords:** brucellosis, meta‐analysis, Middle East, systematic review, West Asia

## Abstract

**Background:**

Brucellosis is a multi‐systemic zoonotic infection. The West Asia/Middle East region is an important global hotspot for brucellosis. This systematic review and meta‐analysis aimed to aggregate and synthesize all the available evidence regarding the complications of brucellosis in West Asia/Middle East region.

**Methods:**

PubMed, Embase, Scopus, Web of Science, Google Scholar, and Proquest were searched. Selection of studies, data extraction, and the risk of bias assessment were performed in duplicate. Data extraction was performed for 254 complications. Meta‐analysis was performed using a random‐effects model with Freeman–Tukey double arcsine transformation. Where applicable small‐study effects was assessed using funnel plots and Egger's test. Separate by‐country, by‐age, and by‐publication‐decade subgroup analyses were performed if feasible.

**Results:**

Out of 9518 results, 240 studies (260 references) were included. The majority of the included studies were conducted in Turkey (*n* = 177). The reported complications varied and different complication categorization systems were detected. The highest pooled estimate was observed for musculoskeletal involvement (50%, 95%CI: 39%–61%, *I* ^2^ = 96.63%). The evidences is up‐to‐date until March 4, 2024.

**Conclusions:**

Some complications such as the complications of the eye were not reported in all the countries. Therefore, it's recommended to determine the relative frequency of those complications in regions without such reports. The pooled estimates of different complications of brucellosis were different. There's a need for the standardization of the reporting of the complications of brucellosis to achieve comparability between studies and across different regions.

## Graphical abstract

In 51.66% of the included studies in West Asia, the sample size was inadequate for at least half of the complications measured.

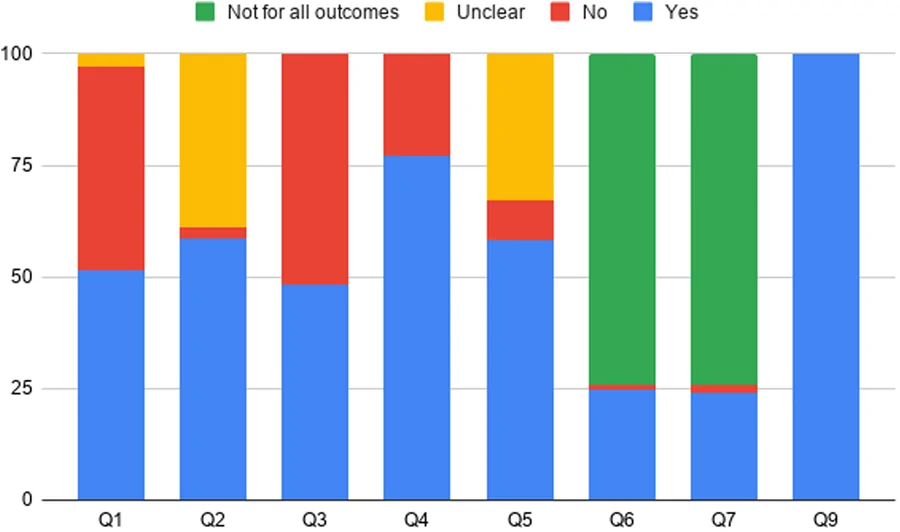

## 1 Introduction

Brucellosis is a zoonotic infection caused by the species of Brucella particularly *Brucella melitensis* (Zhao et al. 2025). The transmission of brucellosis to humans generally occurs via the consumption of unpasteurized dairy products or contact with infected animals (Getachew et al. 2025). However, its airborne transmission has also been documented (Abd El‐Wahab 2025).

In humans, brucellosis infection is multi‐systemic (Safdari 2025). Brucellosis is able to cause complications such as osteo‐articular involvement, cardiovascular complications, hematologic complications, genitourinary complications, gastrointestinal involvement, respiratory involvement, nervous system involvement, cutaneous involvement, and ocular involvement (Pang et al. 2025; Khan et al. 2025). Brucellosis also has a low case fatality rate in humans with endocarditis being considered as the main cause of mortality (Bhat et al. 2024; Ragmoun et al. 2025; Wei et al. 2025). Moreover, for human brucellosis, it has been estimated that treatment costs per patient can range from 340 to 4095 dollars in severe cases (Oreiby et al. 2025). Early and accurate diagnosis of brucellosis is vital for its effective management and treatment (Shi et al. 2024).

The West Asia/Middle East region is an important global hotspot for brucellosis (Holloway et al. 2025) where brucellosis causes a significant public health challenge (Kaki et al. 2025). The pooled prevalence of Brucella spp in milk and other dairy products in the West Asia/Middle East region has been estimated to be 36% (95% confidence interval [CI]: 28%–54%) in raw cow milk, 13% (95%CI: −22% to 48%) in raw buffalo milk, 9% (95%CI: −16% to 35%) in cheese with the overall prevalence of *Brucella* spp. across all products being estimated to be 29% (95%CI: 23%–35%) (Abedi et al. 2020). The pooled prevalence of brucellosis in the West Asia/Middle East region has been estimated to be 20.45% (95% CI: 17.85%–23.19%) with the pooled prevalence being higher in Oman, Lebanon, Palestine, Jordan, and Kuwait (Dadar et al. 2024). However, the relative frequency of the complications of brucellosis in the West Asia/Middle East region is unknown.

Moreover, further research is required to better characterize the clinical course of brucellosis (Di Bari et al. 2022). Therefore, this systematic review and meta‐analysis aimed to aggregate and synthesize all the available evidence regarding the complications of brucellosis in West Asia/Middle East region.

## 2 Methods

This systematic review and meta‐analysis was designed and conducted according to the instructions of JBI Manual for Evidence Synthesis (Aromataris and Munn 2020) and was reported according to the Preferred Reporting Items for Systematic Reviews and Meta‐Analyses 2020 (PRISMA 2020) statement (Page et al. 2021). The study protocol obtained official approval from the Ethics Committee of Torbat Jam Faculty of Medical Sciences [IR.TRJUMS.REC.1403.003].

### 2.1 Inclusion and Exclusion Criteria

The inclusion criteria were studies investigating the relative frequency of any of the complications of brucellosis in adults and children diagnosed with human brucellosis, confirmed through laboratory tests such as serology or Polymerase chain reaction (PCR), in countries within the West Asia region. We defined brucellosis complications as the involvement of any organs due to brucellosis. We accepted the clinical‐ and laboratory‐based definitions of the complications used by the authors of the included studies. The exclusion criteria consisted of studies written in languages other than English or Persian, studies without clear diagnostic confirmation of brucellosis, case reports, and studies on non‐human brucellosis cases.

### 2.2 Search Strategy

PubMed, Embase, Scopus, Web of Science, Google Scholar, and Proquest were searched using two blocks of keywords: “Brucella” OR “Brucellosis” OR “Malta Fever” and the list of countries. The search strategy was translated for each database. No restrictions based on date, language, study design, publication type, or the availability of data were made. This search strategy was a revision to the strategy that was stated in the protocol and it was revised to maximize the comprehensiveness of the search. The search was performed on March 4, 2024 except for Google Scholar for which the search was performed from March 2 to 4, 2024. The search strategy algorithms used for each database are presented in Supporting Information: Table S1. The references retrieved by the search were stored in an EndNote file. Duplicate results were omitted using the EndNote 20 software.

### 2.3 Title and Abstract Screening

ER, FS, AN, FF, FB, HS, MK, and MA independently screened the titles and abstracts of the results from each database. AFD resolved the conflicts risen on the inclusion of studies.

### 2.4 Data Extraction

AFD, AN, HS, MK, and MA independently extracted the data into Google Sheets. The data of each study was extracted independently by two review authors. Due to the limitation in the labor force, we only extracted data for 254 outcomes chosen based on their clinical importance and their frequency of being reported in the included studies. Inconsistencies on the extracted data were resolved by re‐checking the full‐text of the studies (by AFD) and reaching consensus. AFD emailed the corresponding authors of studies with incomplete outcome (numerator or denominator) data requesting the missing information. None of the corresponding authors of studies with incomplete outcome data provided the requested data. Therefore, the missing outcome data of those studies was not included. For studies that reported the percentage of a complication without reporting the numerators, we imputed the numerators according to the reported total number of brucellosis patients in the studies. For studies with two or more reports, we combined the data of the reports.

### 2.5 Risk of Bias Assessment

AFD, MA, and VR independently assessed the risk of bias of the included studies using JBI Critical Appraisal Checklist for Studies Reporting Prevalence data (Munn et al. 2015). The judgements for each item were stored in Google Sheets. Then, the judgements of the two review authors were compared. Conflicts risen on the risk of bias of the included studies were resolved with consensus.

### 2.6 Small‐Study Effects

For outcomes reported in 10 or more number of studies, funnel plots were drawn and Egger's test was performed to assess the small‐study effects. A *p*‐value of 0.05 was considered statistically significant.

### 2.7 Data Synthesis

We used the total number of patients with brucellosis as the denominator for the complications except sex‐specific complications for which we used the total number of brucellosis patients of that sex as the denominator and pregnancy‐related complications for which we used the number of pregnant women with brucellosis as the denominator. We used a random‐effects model for pooled estimation. We applied a Freeman‐Tukey double arcsine transformation before the synthesis in order to stabilize the variance. We used I‐squared and Q statistics to assess the heterogeneity. AFD performed the analyses using STATA 17.0.

### 2.8 Subgroup Analysis

Where feasible, we performed separate subgroup analyses for country, the age of participants (studies conducted on children vs studies conducted on adults), and publication period (2020–2024 vs. 2010–2019 vs. 2001–2009 vs. 1990–1999 vs. 1980–1989 vs. 1970–1979 vs. 1960–1969 vs. 1950–1959).

### 2.9 Sensitivity Analysis

For outcomes for which meta‐analysis was deemed feasible and contained at least three included studies, we performed sensitivity analysis with leave‐one‐out method.

### 2.10 Quality of Evidence

AFD and VR independently assessed the quality of evidence of for each outcome by adopting the applicable components (risk of bias, inconsistency, and imprecision) of the Grading of Recommendations, Assessment, Development and Evaluation (GRADE) approach. Disagreements on the certainty of evidence of the outcomes were resolved by discussion.

## 3 Results

### 3.1 Results of the Search

**Figure 1:**
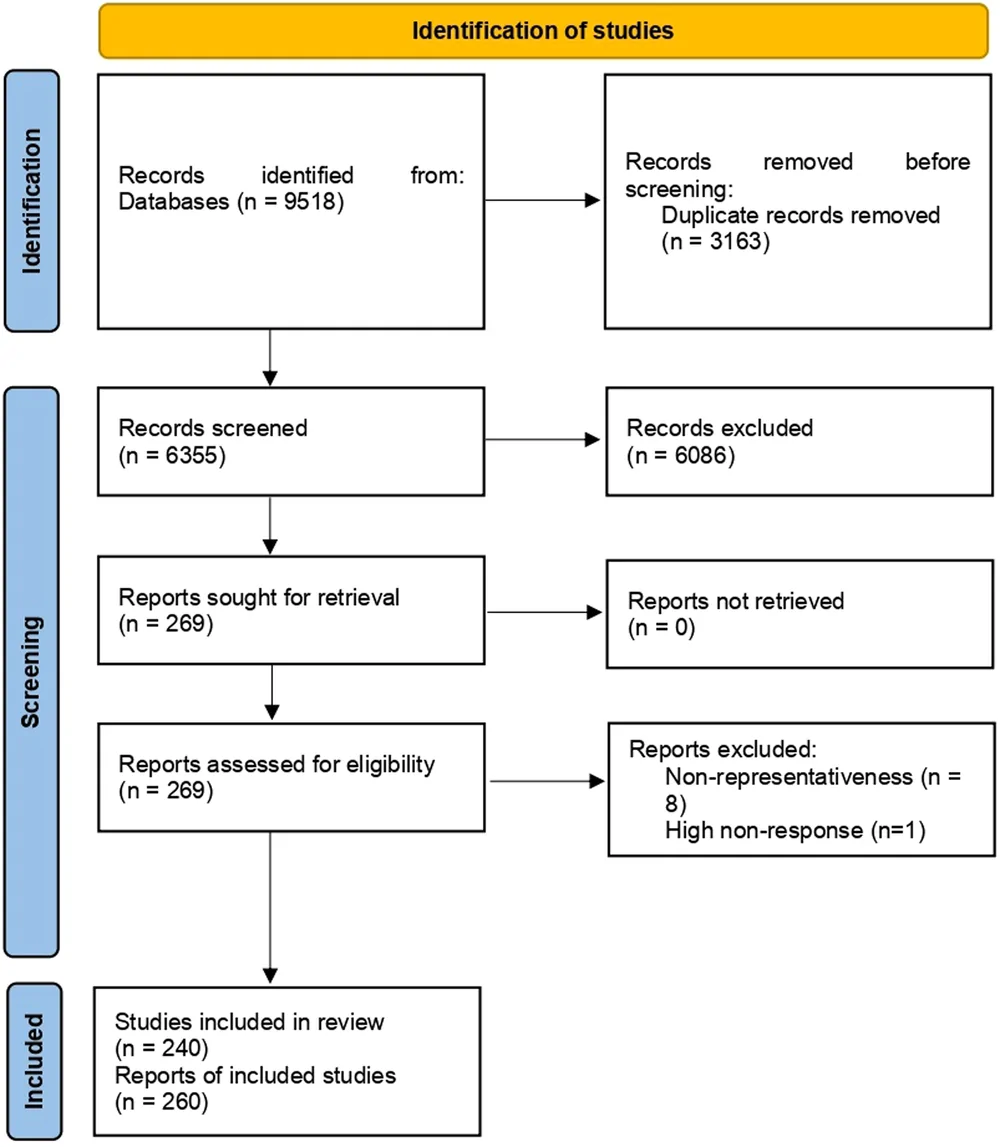
Flow chart of the included studies.

Initially, 9518 results were retrieved by the search. After the removal of 3163 duplicate results, the remaining 6355 results entered the stage of title‐and‐abstract screening of which 6086 results were omitted. In full text screening, nine studies were excluded. Finally, 240 studies belonging to 260 references were included (Figure 1).

### 3.2 Included Studies

Most of the included studies were conducted in Turkey (*n* = 117), Iran (*n* = 57), and Saudi Arabia (*n* = 34). There were 12 studies conducted in Occupied Palestine. Six studies were conducted in Kuwait. Three studies were conducted in Iraq, Jordan, and Oman each. Two studies were performed in Qatar while from Lebanon, United Arab Emirates, and Yemen only one study was included each. The majority of studies included participants of both sexes (*n* = 220) while 10 and seven studies only included males and females respectively. The sex of the participants was not given (NG) in three studies. 68 studies included brucellosis patients of all ages while 81 studies only contained adults and adolescence (adults‐only) and 61 studies only contained children participants (children‐only). The age of the participant was not reported in 30 studies (Table 1).

**Table 1:** Characteristics of the included studies.

	Study ID	Publication year	Country	Type age	Age average	Sex	Sample period
1	Abbasi 2020 (Abbasi 2020)	2020	Iran	Mixed	34.17 ± 17.10 years	Mixed	2011–2015
2	Abd Alrheam 2015 (Abd Alrheam 2015)	2015	Saudi Arabia	Adults only	NG	Mixed	2010– 2014
3	Abdi‐Liae 2007 (Abdi‐Liae 2007)	2007	Iran	Mixed	Median: 38 years	Mixed	1997–2002
4	Afify 2013 (Afify 2013)	2013	Saudi Arabia	Children only	7.6 ± 1.8 years	Mixed	2010–2011
5	Akbayram 2011 (Akbayram 2011a)	2011	Turkey	Children only	NG	Mixed	2004–2010
6	Akbayram 2015 (Akbayram 2015, Karaman 2016)	2015	Turkey	Children only	11.06 ± 4.3 years	Mixed	2009–2015
7	Akcali 2007 (Akcali 2007)	2007	Turkey	Mixed	45.81 ± 15.62 years	Mixed	2000–2002
8	Akdeniz 1998 (Akdeniz 1998)	1998	Turkey	NG	NG	Mixed	1994–1997
9	Akinci 2006 (Akinci 2006)	2006	Turkey	NG	NG	Male	2001–2004
10	Akkoc 2023 (Akkoc 2023)	2023	Turkey	Children only	136 ± 51.6 months	Mixed	2017–2018
11	Al Hashan 2017 (Al Hashan 2017)	2017	Saudi Arabia	Children only	6.1 ± 2.2 years	Mixed	2013–2017
12	Al Nokhatha 2021 (Al Nokhatha 2021)	2021	United Arab Emirates	Adults only	44 years	Mixed	2009–2016
13	Al Sawafi 2023 (Al Sawafi 2023)	2023	Oman	Children only	6.0 ± 3.8 years	Mixed	2006–2020
14	Al Shamahy 2001 (Al Shamahy 2001)	2001	Yemen	Mixed	25.6 years	Mixed	1992–1993
15	Al‐Dubooni 1986 (Al‐Dubooni 1986)	1986	Iraq	Children only	NG	Mixed	1983–1984
16	Al‐Eissa 1993 (Al‐Eissa 1993, Al‐Eissa 1993)	1993	Saudi Arabia	Children only	7.8 years	Mixed	1985–1991
17	Al‐Eissa1990 (Al‐Eissa 1990a, Al‐Eissa 1990b)	1990	Saudi Arabia	Children only	8.2 years	Mixed	1984–1987
18	Al‐Freihi 1986 (Al‐Freihi 1986)	1986	Saudi Arabia	Mixed	Median: 35 years	Mixed	1981–1985
19	Al‐Koubaisy 2011 (Al‐Koubaisy 2011)	2011	Iraq	Adults only	NG	Mixed	2009–2010
20	AlRashidi 2022 (AlRashidi 2022)	2022	Saudi Arabia	Children only	8.2 ± 4.6 years	Mixed	2021
21	Artuk 2019 (Artuk 2019)	2019	Turkey	Mixed	25.62 ± 5.46 years	Mixed	2001–2012
22	Aygen 2002 (Aygen 2002)	2002	Turkey	Adults only	44.1 ± 16.4 years	Mixed	1989–1998
23	Aypak 2012 (Aypak 2012)	2012	Turkey	Mixed	25 ± 18.6 years	Mixed	2010
24	Magableh 2007 (Magableh 2007)	2007	Jordan	Mixed	NG	Mixed	1996–2006
25	Baykan 2019 (Baykan 2019)	2019	Turkey	Mixed	Median: 33 years	Male	2014–2018
26	Bayram 1997 (Bayram 1997)	1997	Turkey	Adults only	NG	Male	1992–1996
27	Beheshti 2001 (Beheshti 2001)	2001	Iran	Mixed	30	Mixed	NG
28	Behzadnia 2018 (Behzadnia 2018)	2018	Iran	Children only	12.5 years	Mixed	2010–2016
29	Benbir 2013 (Benbir 2013)	2013	Turkey	Mixed	42.35 ± 16.46 years	Mixed	NG
30	Benjamin 1992 (Benjamin 1992, Benjamin 1992)	1992	Saudi Arabia	Children only	NG	Mixed	1985–1990
31	Besharat 2010 (Besharat 2010)	2010	Iran	Adults only	NG	Mixed	NG
32	Bodur 2003 (Bodur 2003, Bodur 2004)	2003	Turkey	Adults only	44.2 ± 17.7 years	Mixed	2001–2002
33	Bozdemir 2017 (Bozdemir 2017)	2017	Turkey	Children only	Median: 130 months	Mixed	2013–2016
34	Bozgeyik 2014 (Bozgeyik 2014)	2014	Turkey	Mixed	50.2 years	Mixed	NG
35	Bozgeyik 2008 (Bozgeyik 2008)	2008	Turkey	Mixed	52.8 years	Mixed	2002–2007
36	Bukharie 2009 (Bukharie 2009)	2009	Saudi Arabia	Adults only	Median: 32 ± 9 years	Mixed	1998–2007
37	Bulut 2011 (Bulut 2011)	2011	Turkey	Adults only	44.0 ± 18.3 years	Mixed	1999–2005
38	Caksen 2002 (Caksen 2002, Çaksen 2001)	2002	Turkey	Children only	8.31 ± 3.58 years	Mixed	1994–1998
39	Celen 2010 (Celen 2010)	2010	Turkey	Adults only	NG	Male	1998–2006
40	Celik 2023 (Celik 2023)	2023	Turkey	Adults only	NG	Male	2012–2022
41	Çelik 2023 (Çelik 2023)	2023	Turkey	Adults only	41.79 ± 11.31 years	Mixed	2018
42	Çiftdogan 2020 (Çiftdogan 2020)	2020	Turkey	Children only	7.37 ± 4.14 years	Mixed	2012–2013
43	Çirakli 2015 (Çirakli 2015)	2015	Turkey	Children only	11 years	Mixed	2008–2013
44	Conkar 2018 (Conkar 2018)	2018	Turkey	Children only	12.7 ± 2.97 years	Mixed	2013–2014
45	Coskun 2004 (Coskun 2004)	2004	Turkey	NG	NG	NG	NG
46	Coskun 2009 (Coskun 2009)	2009	Turkey	Adults only	Median: 26 years	Male	1997–2008
47	Dabaja‐Younis 2023 (Dabaja‐Younis 2023)	2023	Occupied Palestine	Mixed	NG	Mixed	2000–2021
48	Dal 2013 (Dal 2013)	2013	Turkey	Adults only	33 years	Mixed	2005–2009
49	Demir 2014 (Demir 2014)	2014	Turkey	Adults only	40.9 ± 15.4 years	Mixed	2012–2013
50	Demirtuerk 2008 (Demirtuerk 2008)	2008	Turkey	NG	NG	Mixed	2002–2006
51	Ebrahimpour 2017 (Ebrahimpour 2017)	2017	Iran	Mixed	NG	Mixed	2005–2015
52	Eini 2012 (Eini 2012a, Eini 2012b)	2012	Iran	Mixed	40.84 ± 20.29 years	Mixed	2005–2010
53	El Bayoumy 2014 (El Bayoumy 2014)	2014	Kuwait	Mixed	42 years	Mixed	2008–2012
54	Çiğdem 2019 (Çiğdem 2019)	2019	Turkey	Children only	7.4 years	Mixed	2016–2018
55	El‐Amin 2001 (El‐Amin 2001)	2001	Oman	Children only	5.6 years±3.343	Mixed	1995–1998
56	El‐Koumi 2013 (El‐Koumi 2013)	2013	Saudi Arabia	Children only	7.6 ± 1.8 years	Mixed	2011–2012
57	Elhassanien 2014 (Elhassanien 2014)	2014	Kuwait	Children only	9.5 ± 3.2 years	Mixed	2008–2013
58	Elshamy 2008 (Elshamy 2008)	2008	Saudi Arabia	Adults only	27 ± 6 years	Female	2005–2007
59	Erat 2023 (Erat 2023)	2023	Turkey	Children only	10.45 ± 4.36 years	Mixed	2019–2021
60	Eroglu 2020 (Eroglu 2020)	2020	Turkey	Adults only	38 years	Mixed	2018
61	Ertek 2006 (Ertek 2006)	2006	Turkey	Adults only	NG	Mixed	1985–2002
62	Esmailpour 2010 (Esmailpour 2010)	2010	Iran	Mixed	NG	Mixed	1998–2006
63	Fallah 2005 (Fallah 2005)	2005	Saudi Arabia	Mixed	NG	Mixed	1995–2001
64	Fruchtman 2020 (Fruchtman 2020, Fruchtman 2015)	2020	Occupied Palestine	Children only	8.48 ± 4.81 year	Mixed	2005–2012
65	Geyik 2002 (Geyik 2002)	2002	Turkey	Mixed	33.14 ± 15.03 years	Mixed	1992–2000
66	Golsha 2011 (Golsha 2011)	2011	Iran	Adults only	NG	Mixed	2004–2008
67	Gonen 2014 (Gonen 2014)	2014	Turkey	Adults only	Median: 47.4 ± 17.1 years	Mixed	2004–2012
68	Gottesman 1996 (Gottesman 1996)	1996	Occupied Palestine	Children only	NG	Mixed	1987–1992
69	Gozdaz 2020 (Gozdas 2020)	2020	Turkey	Adults only	NG	Male	2010–2018
70	Gul 2014 (Gul 2014)	2014	Turkey	Adults only	25.62 ± 5.46 years	Mixed	1997–2011
71	Guler 2014 (Guler 2014)	2014	Turkey	Mixed	39.6 ± 18.2 years	Mixed	2006–2012
72	Güngör 2002 (Güngör 2002)	2002	Turkey	NG	NG	Mixed	1996–2000
73	Gür 2003 (Gür 2003)	2003	Turkey	Mixed	32.69 ± 14.39 years	Mixed	1992–2000
74	Hadadi 2009 (Hadadi 2009)	2009	Iran	Mixed	35.5 ± 11.3 years	Mixed	1998–2005
75	Hamid Hashemi 2016 (Hamid Hashemi 2016)	2016	Iran	NG	NG	NG	2013–2014
76	Hasanjani Roushan 2016a (Hasanjani Roushan 2016a)	2016	Iran	Mixed	34 ± 16.9 years	Mixed	NG
77	Hasanjani Roushan 2004 (Hasanjani Roushan 2004, Hasanjani Roushan 2006, Hasanjani Roushan 2004)	2004	Iran	Adults only	36.97 ± 15 years	Mixed	1997–2003
78	Hasanjani Roushan 2016b (Hasanjani Roushan 2016b)	2016	Iran	Children only	NG	Mixed	2005–2015
79	Hashemi 2007 (Hashemi 2007)	2007	Iran	Mixed	26.9 ± 19.2 years	Mixed	2004–2005
80	Hassan 2021 (Hassan 2021)	2021	Oman	Adults only	Median: 31.5 years	Mixed	2015–2018
81	Hatipoglu 2005 (Hatipoglu 2005)	2005	Turkey	NG	NG	Mixed	2001–2003
82	Heidari 2011 (Heidari 2011)	2011	Iran	NG	35 ± 19 years	Mixed	NG
83	Hizel 2007 (Hizel 2007)	2007	Turkey	Adults only	43 years	Mixed	1997–2003
84	Inan 2019 (Inan 2019)	2019	Turkey	Adults only	28.8 ± 6.28 years	Female	2002–2015
85	Issa 1999 (Issa 1999)	1999	Jordan	Children only	NG	Mixed	NG
86	Kaptan 2013 (Kaptan 2013)	2013	Turkey	Adults only	49 years	Mixed	2005–2008
87	Karaoglan 2008 (Karaoglan 2008)	2008	Turkey	NG	NG	Mixed	2003–2006
88	Karli 2015 (Karli 2015)	2015	Turkey	Children only	NG	Mixed	2008–2013
89	Kayaaslan 2016 (Kayaaslan 2016)	2016	Turkey	Adults only	41.5 ± 17.0 years	Mixed	2002–2014
90	Kazak 2016 (Kazak 2016)	2016	Turkey	Adults only	NG	Mixed	1986–2012
91	Keyvanfar 2021 (Keyvanfar 2021)	2021	Iran	Mixed	43.07 ± 18.521 years	Mixed	2015–2020
92	Khateeb 1990 (Khateeb 1990, Khateeb 1988)	1990	Kuwait	Mixed	33 years	Mixed	1985–1986
93	Kokoglu 2006 (Kokoglu 2006)	2006	Turkey	Adults only	32.2 ± 14.1 years	Mixed	2000–2002
94	Kursun 2013 (Kursun 2013)	2013	Turkey	Adults only	48 ± 17 years	Mixed	2004–2011
95	Kurtaran 2012 (Kurtaran 2012)	2012	Turkey	Adults only	40 ± 17 years	Mixed	1997–2010
96	Kutlu 2018 (Kutlu 2018)	2018	Turkey	Adults only	48.5 ± 15.9 year	Mixed	2007–2016
97	Lazutkin 2018 (Lazutkin 2018)	2018	Occupied Palestine	Mixed	36 years	Mixed	2007–2016
98	Madkour 1988 (Madkour 1988)	1988	Saudi Arabia	Mixed	NG	Mixed	1986–1987
99	Majzoobi 2023 (Majzoobi 2023)	2023	Iran	Adults only	27.84 ± 6.13 years	Female	2018–2020
100	Malik 1997 (Malik 1997)	1997	Saudi Arabia	Adults only	32 years	Mixed	1986–1989
101	McLean 1992 (McLean 1992)	1992	Saudi Arabia	NG	NG	Mixed	1983–1991
102	Megged 2016 (Megged 2016)	2016	Occupied Palestine	Mixed	NG	Mixed	2014
103	Ulug 2011 (Ulug 2011)	2011	Turkey	Adults only	36.4 ± 14.2 years	Mixed	2007–2008
104	Mermut 2011 (Mermut 2011)	2011	Turkey	Adults only	NG	Mixed	1996–2008
105	Mohamed 1986 (Mohamed 1986)	1986	Saudi Arabia	Mixed	NG	Mixed	1981–1982
106	Mohammad Saeed 2012 (Mohammad Saeed 2012)	2012	Iran	Children only	8.02 years	Mixed	2003–2006
107	Mousa 1988 (Mousa 1988a, Mousa 1988b, Mousa 1987)	1988	Kuwait	Mixed	NG	Mixed	1983–1986
108	Mugahi 2014 (Mugahi 2014)	2014	Iran	Mixed	38.1 years	Mixed	1998–2007
109	Nabavi 2019 (Nabavi 2019)	2019	Iran	Mixed	39.59 ± 17.28 years	Mixed	2009–2015
110	Najari 2018 (Najari 2018)	2018	Iran	Adults only	42.5 ± 18.3 years	Mixed	2012–2015
111	Namiduru 2003 (Namiduru 2003, Namiduru 2004)	2003	Turkey	Adults only	NG	Mixed	1995–2000
112	Norton 1984 (Norton 1984)	1984	Saudi Arabia	Mixed	NG	Mixed	1977‐1984
113	Okur 2012 (Okur 2012)	2012	Turkey	Children only	9.14 ± 3.4 years	Mixed	2009–2010
114	Özdem 2022 (Özdem 2022)	2022	Turkey	Children only	Median: 10.4 years	Mixed	2005–2021
115	Özen 2022 (Özen 2022)	2022	Turkey	Children only	Median: 11.3 years	Mixed	2018–2021
116	Parlak 2015 (Parlak 2015)	2015	Turkey	Children only	10.0 ± 3.95 years	Mixed	2009–2013
117	Rajapakse 1987 (Rajapakse 1987)	1987	Saudi Arabia	NG	NG	Mixed	1982–1985
118	Rashad 2019 (Rashad 2019)	2019	Saudi Arabia	Children only	NG	NG	2016–2017
119	Roushan 2005 (Roushan 2005)	2005	Iran	Children only	10.49 ± 3.49 years	Mixed	1999–2003
120	Roushan 2011 (Roushan 2011)	2011	Iran	Adults only	25.0 ± 4.62 years	Female	2000–2010
121	Salman 2022 (Salman 2022)	2022	Turkey	Children only	10.7 ± 4.5 years	Mixed	2011–2021
122	Sanivar 2017 (Sanivar 2017)	2017	Turkey	Adults only	37.90 ± 13.64 years	Mixed	2013–2014
123	Savas 2007 (Savas 2007)	2007	Turkey	Mixed	45.81 ± 15.62 years	Mixed	2000–2002
124	Savion 2023 (Savion 2023)	2023	Occupied Palestine	Children only	11.01 ± 4.914 years	Mixed	2010–2020
125	Sayad 2019 (Sayad 2019)	2019	Iran	NG	NG	Mixed	2011–2016
126	Shahnaz 2007 (Shahnaz 2007)	2007	Iran	Children only	7.3 years	Mixed	1996–2005
127	Shalev 1994 (Shalev 1994)	1994	Occupied Palestine	Children only	NG	Mixed	1989
128	Zia Sheikholeslami 2007 (Zia Sheikholeslami 2007)	2007	Iran	NG	NG	Mixed	2006
129	Simsek 2011 (Simsek 2011)	2011	Turkey	NG	NG	Mixed	1998–2008
130	Somily 2017 (Somily 2017)	2017	Saudi Arabia	Mixed	29 ± 8 years	Mixed	2003–2013
131	Sungur 2009 (Sungur 2009)	2009	Turkey	Mixed	NG	Mixed	1992–2006
132	Tanir 2009 (Tanir 2009)	2009	Turkey	Children only	9.02 ± 3.59 years	Mixed	1997–2006
133	Tasbakan 2003 (Tasbakan 2003)	2003	Turkey	Adults only	40.2 years	Mixed	1996–2001
134	Tasova 1999 (Tasova 1999)	1999	Turkey	Adults only	32.3 ± 14 years	Mixed	1990–1997
135	Teke 2015 (Teke 2015)	2015	Turkey	Children only	NG	Mixed	2005–2011
136	Tekin 2012 (Tekin 2012)	2012	Turkey	Adults only	32.8 ± 15.3	Mixed	2007–2009
137	Tohmé 2004 (Tohmé 2004, Tohme 2004, Tohmé 2001)	2004	Lebanon	Mixed	38 ± 21 years	Mixed	1994–2003
138	Turan 2011 (Turan 2011)	2011	Turkey	Adults only	46.7 ± 18 years	Mixed	2003–2009
139	Ulu‐Kilic 2013 (Ulu‐Kilic 2013)	2012	Turkey	NG	NG	Mixed	2008–2011
140	Varikkodan 2024 (Varikkodan 2024)	2024	Qatar	Adults only	39.6 ± 15.05 years	Mixed	2015–2020
141	Yetkin 2005 (Yetkin 2005, Yetkin 2006a, Yetkin 2006b)	2006	Turkey	Adults only	NG	Mixed	1999–2004
142	Yilmaz 2004 (Yilmaz 2004)	2004	Turkey	NG	NG	Mixed	1986–2001
143	Zaks 1995 (Zaks 1995)	1995	Occupied Palestine	Mixed	25 years	Mixed	1972–1990
144	Zamani 2011 (Zamani 2011)	2011	Iran	Children only	NG	Mixed	1997–2005
145	Abbasi 2012 (Abbasi 2012, Asadipooya 2011)	2012	Iran	NG	NG	Mixed	NG
146	Alsubaie 2005 (Alsubaie 2005)	2005	Saudi Arabia	Mixed	NG	Mixed	2001–2002
147	Kayaaslan 2013 (Kayaaslan 2013)	2013	Turkey	Adults only	47 ± 17 years	Mixed	2005–2008
148	Kiel 1987 (Kiel 1987)	1987	Saudi Arabia	NG	32 years	Mixed	1983–1986
149	Akbayram 2011 (Akbayram 2011b)	2011	Turkey	Children only	NG	Mixed	2004‐2010
150	Kisacik 2011 (Kisacik 2011)	2011	Turkey	NG	32.2 ± 10.1 years	Mixed	NG
151	Sagi 2017 (Sagi 2017)	2017	Occupied Palestine	Adults only	39.2 ± 16.1 years	Mixed	2015–2016
152	Mousa 1986 (Mousa 1986)	1986	Kuwait	NG	NG	Mixed	1985
153	Kutlu 2009 (Kutlu 2009)	2009	Turkey	Adults only	43.08 ± 15.3 years	Mixed	2003–2004
154	Hasanjani Roushan 2009 (Hasanjani Roushan 2009)	2009	Iran	NG	NG	Male	1998–2008
155	Samra 1983 (Samra 1983)	1983	Occupied Palestine	Mixed	NG	Mixed	1955–1977
156	Feiz 1978 (Feiz 1978)	1978	Iran	Children only	Median: 9.7 years	Mixed	NG
157	Turunç 2008 (Turunç 2008)	2008	Turkey	Adults only	NG	Mixed	2001–2006
158	Naz 2009 (Naz 2009)	2009	Turkey	Adults only	46.93 ± 17.65 years	Mixed	2001–2006
159	Opawoye 1994 (Opawoye 1994)	1994	Saudi Arabia	Children only	NG	Mixed	1992–1993
160	Al Shaalan 2002 (Al Shaalan 2002)	2002	Saudi Arabia	Children only	5.8 years	Mixed	1984–1995
161	Memish 2001 (Memish 2001)	2001	Saudi Arabia	Adults only	NG	Mixed	1991–2000
162	Nabi 1992 (Nabi 1992)	1992	Saudi Arabia	Children only	NG	Mixed	NG
163	Gulsun 2011 (Gulsun 2011)	2011	Turkey	Adults only	28.07 years	Female	2003–2010
164	Kurdoglu 2010 (Kurdoglu 2010)	2010	Turkey	Adults only	26.79 ± 5.07 years	Female	2003–2008
165	Khan 2001 (Khan 2001)	2001	Saudi Arabia	Adults only	26.9 years	Female	1983–1995
166	Rahil 2014 (Rahil 2014)	2014	Qatar	Adults only	NG	Mixed	2000–2006
167	Qasim 2020 (Qasim 2020)	2020	Saudi Arabia	Children only	7.75 ± 3.28 years	Mixed	2015–2019
168	Suvak 2016 (Suvak 2016)	2016	Turkey	Adults only	33.6 years	Mixed	2013–2014
169	Türker 2014 (Türker 2014)	2014	Turkey	Adults only	NG	Mixed	1997–2012
170	Patel 1988 (Patel 1988)	1988	Saudi Arabia	Mixed	NG	Mixed	NG
171	Ertekin 2004 (Ertekin 2004)	2004	Turkey	Children only	7.1 ± 4.2 years	Mixed	1996–2001
172	Torkaman 2017 (Torkaman 2017)	2017	Iran	Mixed	41.1 ± 17.2 years	Mixed	2013–2014
173	Naz 2016 (Naz 2016)	2016	Turkey	Adults only	NG	Male	2001–2013
174	Uluğ 2011 (Uluğ 2011)	2011	Turkey	Children only	8.9 ± 2.8 years	Mixed	2007–2008
175	Yoldas 2015 (Yoldas 2015)	2015	Turkey	Children only	10 ± 3.8 years	Mixed	2000–2010
176	Buzgan 2010 (Buzgan 2010)	2010	Turkey	Mixed	33.7 ± 16.34 years	Mixed	1998–2007
177	Edathodu 2021 (Edathodu 2021)	2021	Saudi Arabia	Adults only	Median: 50 years	Mixed	2003–2018
178	Köse 2014 (Köse 2014)	2014	Turkey	Adults only	44.8 ± 18 years	Mixed	2004–2010
179	Fanni 2013 (Fanni 2013)	2013	Iran	Children only	6.88 years	Mixed	2002–2010
180	Kaman 2022 (Kaman 2022)	2022	Turkey	Children only	9.4 ± 4.7 years	Mixed	2005–2018
181	Keramat 2009 (Keramat 2009)	2009	Iran	Adults only	40.69 years	Mixed	2002–2006
182	Keramat 2018 (Keramat 2018)	2018	Iran	Adults only	NG	Mixed	2016–2017
183	Metin 2001 (Metin 2001)	2001	Turkey	Mixed	30.45 ± 15.08 years	Mixed	NG
184	Kaya 2018 (Kaya 2018)	2018	Turkey	Adults only	33.88 ± 14.96 years	Mixed	2009–2013
185	Shehabi 1990 (Shehabi 1990)	1990	Jordan	Mixed	27±17years	Mixed	1987
186	Aktug‐Demir 2014 (Aktug‐Demir 2014)	2014	Turkey	Adults only	43.1 ± 15.2 years	Mixed	2010–2012
187	Najafi 2018 (Najafi 2018, Najafi 2014)	2014	Iran	Mixed	41.6 ± 16.9 years	Mixed	2009–2014
188	Rahmanpour 2019 (Rahmanpour 2019)	2019	Iran	NG	39.89 ± 16.11 years	Mixed	2017–2018
189	Majzoobi 2018 (Majzoobi 2018)	2018	Iran	Adults only	NG	Mixed	2015–2016
190	Saltoglu 2002 (Saltoglu 2002)	2002	Turkey	Adults only	36.8 ± 11.3 years	Mixed	1997–2001
191	Pourbagher 2006 (Pourbagher 2006)	2006	Turkey	Mixed	NG	Mixed	2000–2004
192	Pourakbari 2019 (Pourakbari 2019)	2019	Iran	Children only	7.02 ± 3.5 years	Mixed	2011–2016
193	Firouzeh 2019 (Firouzeh 2019)	2019	Iran	Mixed	NG	Mixed	2013–2018
194	El‐Kafas 1999 (El‐Kafas 1999)	1999	Saudi Arabia	Children only	NG	Mixed	1996–1998
195	Mohammadbeigi 2021 (Mohammadbeigi 2021)	2021	Iran	Mixed	39.87 ± 19.93 years	Mixed	2015–2019
196	Hatami 2019 (Hatami 2019)	2019	Iran	Mixed	35.05 ± 17.35 years	Mixed	2012–2016
197	Hatami 2010 (Hatami 2010)	2010	Iran	Mixed	NG	Mixed	1988–2005
198	Ayatollahi 2004 (Ayatollahi 2004)	2004	Iran	Mixed	NG	Mixed	1995–2001
199	Sabbaghian 1974 (Sabbaghian 1974)	1974	Iran	Mixed	NG	Mixed	1967–1972
200	Vafaei 2019 (Vafaei 2019)	2019	Iran	Children only	80.5 months	Mixed	2007–2016
201	Biglarzadeh 2020 (Biglarzadeh 2020)	2020	Iran	NG	NG	Mixed	2013–2017
202	Kuruoglu 2023 (Kuruoglu 2023)	2023	Turkey	Adults only	46.4 ± 15.8 years	Mixed	2010–2022
203	Demir 2021 (Demir 2021)	2021	Turkey	Children only	122 ± 54 months	Mixed	2017–2019
204	Zilberman 2022 (Zilberman 2022)	2022	Occupied Palestine	Mixed	NG	Mixed	2017–2019
205	Bayazit 2002 (Bayazit 2002)	2002	Turkey	Adults only	40.5 years	Mixed	NG
206	Citak 2010 (Citak 2010)	2010	Turkey	Children only	NG	Mixed	2004–2009
207	Aypak 2015 (Aypak 2015)	2015	Turkey	Mixed	14.5 ± 3.3 years	Mixed	2010–2011
208	Gholami 2012 (Gholami 2012)	2012	Iran	NG	NG	Mixed	2009–2011
209	Shimol 2012 (Shimol 2012)	2012	Occupied Palestine	Mixed	NG	Mixed	2011
210	Mohamed 2018 (Mohamed 2018)	2018	Iraq	Adults only	NG	Mixed	2017–2018
211	Ibrahim 2021 (Ibrahim 2021)	2021	Saudi Arabia	Mixed	35.1 ± 21.2 years	Mixed	2015–2019
212	Hashemi 2018 (Hashemi 2018)	2018	Iran	NG	42.2 ± 18.1 years	Mixed	2016
213	Eskandari‐Nasab 2014 (Eskandari‐Nasab 2014)	2014	Iran	Mixed	31.24 ± 16.6 years	Mixed	NG
214	Kaygusuz 2005 (Kaygusuz 2005)	2005	Turkey	Mixed	31.62 ± 13.97 years	Mixed	1999–2002
215	Sayin‐Kutlu 2012 (Sayin‐Kutlu 2012)	2012	Turkey	Adults only	32 ± 6 years	Mixed	2010–2011
216	Sari 2008 (Sari 2008)	2008	Turkey	Adults only	NG	Mixed	1999–2005
217	Demiraslan 2009 (Demiraslan 2009)	2009	Turkey	Adults only	NG	Mixed	1997–2006
218	Guven 2013 (Guven 2013)	2013	Turkey	Adults only	NG	Mixed	2002–2005
219	Ranjbar 2009 (Ranjbar 2009)	2009	Iran	NG	NG	Mixed	2001–2005
220	Karabay 2004 (Karabay 2004)	2004	Turkey	Adults only	NG	Mixed	1999–2001
221	Karakurt 2023 (Karakurt 2023)	2023	Turkey	NG	NG	Mixed	2016–2018
222	Ertem 2004 (Ertem 2004)	2004	Turkey	Adults only	43 years	Mixed	1999–2002
223	Paul 2017 (Paul 2017)	2017	Saudi Arabia	Mixed	28.50 ± 13.65 years	Mixed	2014–2016
224	Karakukcu 2004 (Karakukcu 2004)	2004	Turkey	Children only	NG	Mixed	1996–2003
225	Olt 2015 (Olt 2015)	2015	Turkey	NG	41.7 ± 16.1 years	Mixed	NG
226	Sen 2019 (Sen 2019)	2019	Turkey	Adults only	45.4 ± 17.4 years	Mixed	2010–2016
227	Kara 2019 (Kara 2019)	2019	Turkey	Children only	10.9 ± 3.9 years	Mixed	2015
228	Aghaali 2015 (Aghaali 2015)	2015	Iran	Children only	NG	Mixed	2013
229	Ahmadinejad 2016 (Ahmadinejad 2016)	2016	Iran	Mixed	27.6 ± 20.6 years	Mixed	2012
230	Lubani 1989 (Lubani 1989)	1989	Kuwait	Mixed	NG	Mixed	NG
231	Salari 2003 (Salari 2003)	2003	Iran	Mixed	NG	Mixed	1993–1998
232	Cuzdan 2022 (Cuzdan 2022)	2022	Turkey	Adults only	46.58 ± 15.43 years	Mixed	2017–2020
233	Alim 2015 (Alim 2015)	2015	Turkey	Mixed	NG	Mixed	2011
234	Kahbazi 2012 (Kahbazi 2012)	2012	Iran	Children only	NG	Mixed	2011
235	Lifeso 1985 (Lifeso 1985)	1985	Saudi Arabia	NG	NG	Mixed	1976–1983
236	Kadanali 2005 (Kadanali 2005)	2005	Turkey	Adults only	33.7 ± 15.8 years	Mixed	2001–2004
237	Rezazadeh 2006 (Rezazadeh 2006)	2006	Iran	Mixed	40.8 ± 19 years	Mixed	2004–2005
238	Çiftdoğan 2017 (Çiftdoğan 2017)	2017	Turkey	Mixed	NG	Mixed	2012–2013
239	Najafi 2011 (Najafi 2011)	2011	Iran	NG	NG	Male	1997–2009
240	Ergun 2008 (Ergun 2008)	2008	Turkey	Adults only	NG	Mixed	2004–2005

Abbreviation: NG, not given.

Overall, the data of 61,171 individuals with brucellosis were synthesized in this systematic review. Among the studies that reported the sex of the individuals, 29,481 individuals were male while 15,671 of the individuals were female. The majority of individuals were studied only in hospital settings either as inpatients or outpatients (*N* = 185) while in nine additional studies, the subjects were from hospitals combined with another source including medical school (*N* = 1), health station (*N* = 1), patient family members (*N* = 3), and clinic (*N* = 4). Four studies were conducted solely in community settings. Among the studies that reported the method of diagnosis of Brucellosis, the majority used a combination of diagnostic methods such as bacterial cultures and/or clinical findings combined with serology techniques such as 2‐mercaptoethanol (2‐ME). Serology techniques were the predominant methods of diagnosis and were used in the eligibility criteria in 220 studies while bacterial isolation/culture procedures were used as an inclusion criterion in 173 studies. PCR was only used in two studies (Supporting Information: Table S2–1).

Among the included studies, the reported complications varied and different categorizations were detected. Some studies had used system‐level outcomes such as osteoarticular involvement, musculoskeletal involvement, and nervous system involvement while some studies measured organ‐level involvement such as orchitis, kidney involvement, and lung involvement while other studies studied more specific outcomes such as nephrotic syndrome, hydrocephalus, optic neuritis, and endocarditis. Some studies used vague outcomes such as “spondylitis or sacroillitis”, “paravertebral or psoas abscess”, “permanent aspermia or oligospermia”, “pneumonitis or bronchiolitis”, “focal involvement”, and “local abscess”. The full list of the 254 included outcomes is reported in Supporting Information: Table S3–1.

### 3.3 Excluded Studies

We excluded nine studies due to Non‐representativeness and high non‐response in full‐text screening (Table 2).

**Table 2:** Characteristics of excluded studies.

	Study ID	Reason for exclusion
1	Aydin 2005 (Aydin 2005)	Not representative
2	Rahmanian 2019 (Rahmanian 2019)	Not representative
3	Young 2014 (Young 2014)	High non‐response
4	Rathi 1993 (Rathi 1993)	Not representative
5	Dokuzoğuz 2005 (Dokuzoğuz 2005)	Not representative
6	Erdem 2016 (Erdem 2016)	Not representative
7	Eren 2006 (Eren 2006)	Not representative
8	Guenlue 2022 (Guenlue 2022)	Not representative
9	Mousa 1990 (Mousa 1990)	Not representative

### 3.4 Risk of Bias in Included Studies

Overall, the highest risk of bias in the included studies was related to inappropriateness of the sample frame (studies limiting their inclusion criteria to children or adults) and the inadequacy of their sample sizes in relation to the complications whose relative frequency they were to measure. In 51.66% of the included studies, the sample size was inadequate for at least half of the complications measured in the study. The risk of bias was summarized in Supporting Information: Table S4–1 per study while the overall risk of bias for each outcome was reported in Supporting Information: Tables S4–2 to S4‐255.

### 3.5 Effect Sizes

We were able to perform meta‐analysis for 152 outcomes while for the rest, only one study was included for each of them and meta‐analysis was not feasible. For those outcomes, we reported the point estimates of the single studies instead. In the main‐text, we focused on the outcomes with more clinical importance or with interesting findings. The pooled estimates of all meta‐analyses are reported in Supporting Information: Table S3–1. The funnel plots of the complications with at least 10 number of included studies are presented in Supporting Information: Figures S5–1 to S5‐40. The findings of sensitivity analysis are presented in Supporting Information: Figures S6–1 to S6‐105. The findings of subgroup analyses by country, age group, and publication year are reported in Supporting Information: S7 (Figures S7–1 to S7‐152), S8 (Figures S8–1 to S8‐126), and S9 (Figures S9–1 to S9‐152). The quality of evidence for each outcome is reported in Supporting Information: Table S10–1. The PRISMA checklist is reported in Supporting Information: S11. The pooled estimates of 10 selected complications are presented in Figure 2.

**Figure 2:**
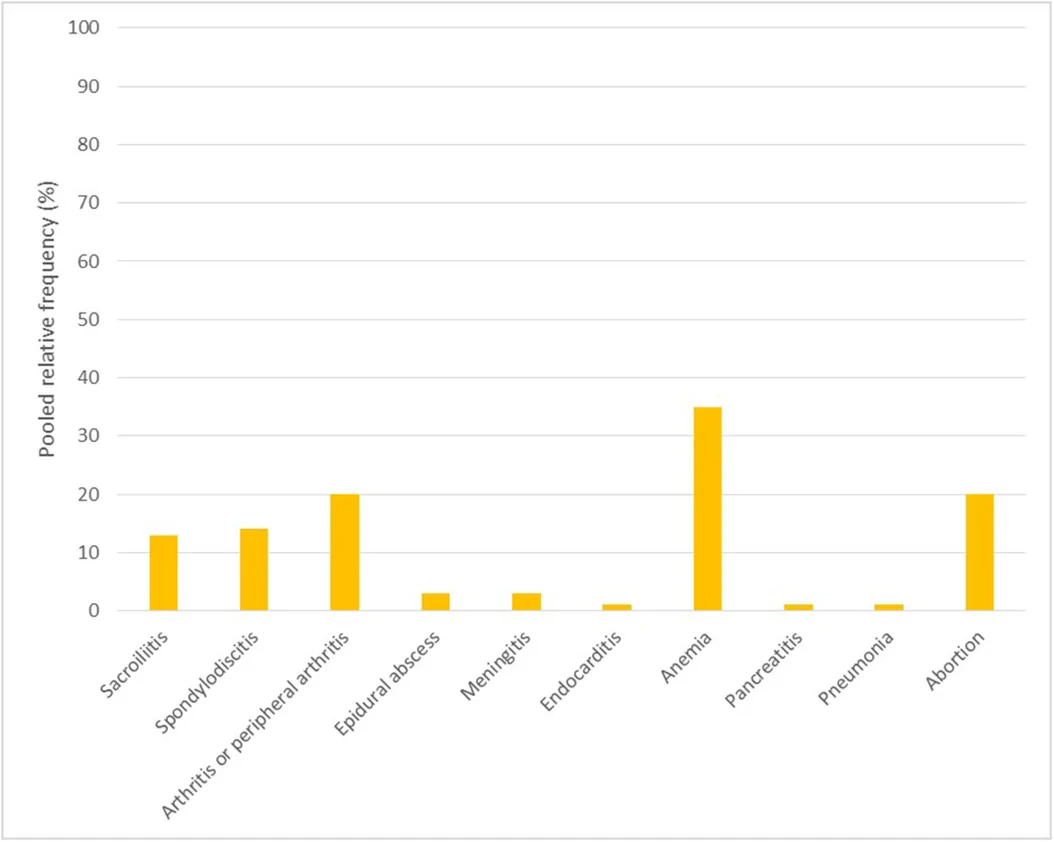
Pooled estimates of 10 selected complications of brucellosis in West Asia.

### 3.6 Complications of the Bones, Joints, Tendons, and Paravertebral or Periarticular Soft Tissue

The pooled estimates for arthritis or peripheral arthritis, spondylodiscitis, and sacroilitis were 20% (95%CI: 17%–23%, *I* ^2^ = 97.70%; very low certainty of evidence), 14% (95%CI: 11%–18%, *I* ^2^ = 92.05%; very low certainty of evidence), and 13% (95%CI: 11%–16%, *I* ^2^ = 96.56%; very low certainty of evidence), respectively. The funnel plots of musculoskeletal involvement, osteoarticular involvement, spondylitis, spondylodiscitis, spinal brucellosis, arthritis or peripheral arthritis, and osteomyelitis showed signs of small‐study effects. Egger's test was statistically significant for Arthritis or peripheral arthritis (*p* < 0.001). For the rest of the outcomes in this category, Egger's test was not statistically significant (*p* > 0.05). In sensitivity analysis with leave‐one‐out method, the pooled estimates for skeletal involvement and arthropathy were found to be not robust. The pooled estimates for the rest of the outcomes in this category were deemed to be robust.

In subgroup analysis by country, the pooled estimate of sacroilitis was higher in Turkey (15%, 95%CI: 11%–19%) and Iran (14%, 95%CI: 8%–21%). The highest pooled estimates of spondylitis (9%, 95%CI: 6%–13%) and spondylodiscitis (15%, 95%CI: 12%–18%) were observed in Turkey. The pooled estimate of spinal brucellosis was the highest in Saudi Arabia (14%, 95%CI: 3%–32%). The pooled estimate for arthritis or peripheral arthritis was the highest in Kuwait (41%, 95%CI: 26%–58%). The pooled estimate of Occupied Palestine for osteomyelitis (8%, 95%CI: 3%–16%) was the highest. The pooled estimate for bursitis in Turkey (2%, 95%CI: 1%–3%) was the highest.

In subgroup analysis by age, the pooled estimate of sacroilitis was 15% (95%CI: 11%–20%) in adults‐only studies vs 4% (95%CI: 2%–6%) in children‐only studies. The pooled estimate of Spondylitis in adults‐only studies was 11% (95%CI: 7%–14%) while in children‐only studies, the pooled estimate was 1% (95%CI: 0%–2%). However, the pooled relative frequency of Arthritis or peripheral arthritis was higher in children‐only studies (33%, 95%CI: 28%–39%) compared to adults‐only studies (11%, 95%CI: 8%–14%).

### 3.7 Complications of the Genito‐Urinary System

Among the sex‐specific complications of genito‐urinary system, the pooled estimates for epididymoorchitis was 7% (95%CI: 6%–9%, *I* ^2^ = 90.94%; very low certainty of evidence). The pooled estimates for urinary system complications (nephritis, nephrotic syndrome, renal failure, renal abscess, and kidney involvement) were less than 1% and they were rated as very low quality of evidence. The funnel plots of genito‐urinary system involvement, orchitis, and epididymoorchitis depicted signs of small‐study effects. Egger's test was statistically significant for orchitis (*p* = 0.004) and epididymoorchitis (*p* < 0.001). For the rest of the outcomes in this category, Egger's test was not statistically significant (*p* > 0.05). In sensitivity analysis with leave‐one‐out method, the pooled estimates for all the outcomes in this category were deemed to be robust.

In subgroup analysis by country, the pooled estimates of orchitis in male patients (very low certainty of evidence) in Iran (7%, 95%CI: 3%–11%) and Occupied Palestine (7%, 95%CI: 1%–16%) were higher compared to Turkey (4%, 95%CI: 2%–7%) while the pooled estimate of epididymoorchitis was the highest in Iran (9%, 95%CI: 7%–12%) and Kuwait (9%, 95%CI: 7%–12%). Prostatitis (very low certainty of evidence) was only reported in Turkey.

In subgroup analysis by age, the pooled estimate of orchitis in male patients was higher in adults‐only studies (5%, 95%CI: 2%–8%) compared to children‐only studies (1%, 95%CI: 0%–3%). Similarly, epididymoorchitis was more common in adults‐only studies (7%, 95%CI: 5%–9%) compared to children‐only studies (3%, 95%CI: 0%–7%).

In subgroup analysis based on publication year, for orchitis and epididymoorchitis, the pooled estimates for studies published between 2020 and 2024 were 0% (95%CI: 0%–1%, *I* ^2^ = 55.15%) and 3% (95%CI: 2%–5%, *I* ^2^ = 84.65%) and were lower than studies published between 2010 and 2019 (7%, 95%CI: 5%–9%, *I* ^2^ = 58.72% and 8%, 95%CI: 7%–10%, *I* ^2^ = 69.98%).

### 3.8 Complications of the Lymphatic System

In this category, the pooled estimate for lymphadenopathy was the highest (9%, 95%CI: 7%–10%, *I* ^2^ = 91.89%; very low certainty of evidence). The funnel plot of lymphadenopathy showed signs of small‐study effects. However, Egger's test was not statistically significant (*p* = 0.07). In sensitivity analysis with leave‐one‐out method, the pooled estimates for all the outcomes in this category were deemed robust.

In subgroup analysis by country, the highest pooled estimate for lymphadenopathy was reported in Occupied Palestine (16%, 95%CI: 11%–22%) while a single study in Yemen in 2001 reported the point estimate of 34% (95%CI: 28%–40%).

In subgroup analysis by age, the pooled estimate of lymphadenopathy was higher in children‐only studies (14%, 95%CI: 10%–19%) compared to adults‐only studies (6%, 95%CI: 5%–8%).

### 3.9 Complications of the Nervous System and Psychologic Disorders

In this category, the pooled estimates for meningitis, brain abscess, and cranial nerve involvement were 3% (95%CI: 2%–3%, *I* ^2^ = 80.26%; very low certainty of evidence), 1% (95%CI: 1%–3%, *I* ^2^ = 71.06%; very low certainty of evidence), and 2% (95%CI: 1%–3%, *I* ^2^ = 44.26%; very low certainty of evidence). Except for one outcome (deep sensory loss) for which meta‐analysis was not feasible, all the outcomes in this category were rated as very low certainty of evidence. The funnel plots of nervous system involvement, central nervous system involvement, neurobrucellosis, neuropsychiatric involvement, meningitis, cranial nerve involvement, hearing loss, and depression showed signs of small‐study effects. Egger's test was not statistically significant for any of the outcomes in this category (*p* > 0.05). In sensitivity analysis with leave‐one‐out method, the pooled estimate for psychiatric involvement was not robust. For the rest of the outcomes, the pooled estimates were deemed to be robust in this category.

In subgroup analysis by country, the pooled estimate for neurobrucellosis was higher in Turkey (4%, 95%CI: 3%–6%) and Iran (4%, 95%CI: 2%–6%) while in Occupied Palestine, its pooled estimate was less than 1% (95%CI: 0%–1%). Cranial nerve involvement was only reported in Turkey (2%, 95%CI: 1%–4%) and a single study in Kuwait (2%, 95%CI: 1%–4%). The pooled estimate for hearing loss in Turkey (2%, 95%CI: 0%–6%) was higher than Saudi Arabia (1%, 95%CI: 0%–3%). The pooled estimate for depression was the highest in Iran (8%, 95%CI: 0%–36%).

In subgroup analysis by age, the pooled estimate of central nervous system involvement was higher in adults‐only studies (5%, 95%CI: 2%–9%) compared to children‐only studies (less than 1%, 95%CI: 0%–2%). The pooled estimate of neurobrucellosis was higher in adults‐only studies (4%, 95%CI: 3%–6%) compared to children‐only studies (2%, 95%CI: 1%–3%) The pooled estimate of meningitis was higher in adults‐only studies (4%, 95%CI: 2%–5%) compared to children‐only studies (1%, 95%CI: 0%–4%).

In subgroup analysis based on publication year for meningitis, the pooled estimate of the subgroup of studies published between 2020 and 2024 (1%, 95%CI: 0%–2%, *I* ^2^ = 0%) was lower compared to studies published between 2010 and 2019 (3%, 95%CI: 2%–4%, *I* ^2^ = 72.73%). For cranial nerve involvement, the pooled estimate of the subgroup of studies published between 2010 and 2019 (4%, 95%CI: 2%–7%, *I* ^2^ not calculable) was higher compared to studies published between 2000 and 2009 (1%, 95%CI: 0%–2%, *I* ^2^ = 11.91%).

### 3.10 Complications of the Cardiovascular System

In this category, except for “perimyocarditis” and “aortitis and aortic rupture” for which meta‐analysis was not feasible, the outcomes were rated as very low certainty of evidence. The pooled estimate for anemia was 35% (95%CI: 31%–39%, *I* ^2^ = 96.39%; very low certainty of evidence) and the pooled estimate for endocarditis was 1% (95%CI: 0%–1%, *I* ^2^ = 39.61%; very low certainty of evidence). The funnel plots of endocarditis, hematologic involvement, leukopenia, leukocytosis, neutropenia, anemia, thrombocytopenia, thrombocytosis, and pancytopenia showed signs of small‐study effects. Egger's test was statistically significant for leukopenia (*p* = 0.01) and neutropenia (*p* = 0.02). For the rest of the outcomes in this category, Egger's test was not statistically significant (*p* > 0.05). In sensitivity analysis with leave‐one‐out method, the pooled estimates for all the outcomes in this category were deemed to be robust.

Capillary leak syndrome was only reported in Turkey (less than 1%, 95%CI: 0%–1%; very low certainty of evidence). In subgroup analysis by country, the pooled estimate for leukopenia was the highest in Saudi Arabia (23%, 95%CI: 14%–34%). However, the pooled estimate for leucocytosis was the highest in Iran (12%, 95%CI: 10%–15%) and Saudi Arabia (12%, 95%CI: 7%–18%). The pooled estimate for anemia was the highest in Saudi Arabia (41%, 95%CI: 33%–49%). The pooled estimates for thrombocytopenia (13%, 95%CI: 9%–16%) and thrombocytosis (6%, 95%CI: 5%–8%) were the highest in Turkey. The pooled estimate for pancytopenia in Occupied Palestine (12%, 95%CI: 1%–32%) was the highest. Hemophagocytosis was only reported in Turkey (2%, 95%CI: 1%–2%).

In subgroup analysis by age, the pooled estimate for endocarditis was higher in adults‐only studies (1%, 95%CI: 1%–1%) compared to children‐only studies (less than 1%, 95%CI: 0%–1%). The pooled estimates of leukopenia (19%, 95%CI: 14%–24% vs. 12%, 95%CI: 10%–15%), leucocytosis (12%, 95%CI: 9%–15% vs. 7%, 95%CI: 5%–9%), thrombocytosis (6%, 95%CI: 3%–9% vs. 5%, 95%CI: 2%–10%), and pancytopenia (7%, 95%CI: 5%–10% vs. 5%, 95%CI: 3%–6%) were higher in children‐only studies compared to adults‐only studies. However, the pooled estimates of lymphocytosis (54%, 95%CI: 40%–68% vs. 18%, 95%CI: 3%–41%) and thrombocytopenia (13%, 95%CI: 8%–19% vs. 9%, 95%CI: 7%–11%) were higher in adults‐only studies compared to children‐only studies.

### 3.11 Complications of the Skin

In this category, the highest pooled estimate was observed for cutaneous involvement (4%, 95%CI: 3%–6%, *I* ^2^ = 91.90%; very low certainty of evidence). The funnel plot of cutaneous involvement was deemed to be affected by small‐study effects. However, Egger's test was not statistically significant (*p* = 0.77). In sensitivity analysis with leave‐one‐out method, the pooled estimates for all the outcomes classified in this category were deemed to be robust.

In subgroup analysis by country, the pooled estimate for cutaneous involvement in Occupied Palestine (8%, 95%CI: 5%–11%) was the highest. The pooled estimate for petechiae and purpura (very low certainty of evidence) in Saudi Arabia (5%, 95%CI: 2%–10%) was higher compared to Turkey (2%, 95%CI: 0%–7%).

In subgroup analysis by age, the pooled estimate of cutaneous involvement was higher in children‐only studies (4%, 95%CI: 2%–8%) compared to adults‐only studies (3%, 95%CI: 1%–5%).

### 3.12 Complications of the Gastrointestinal and Hepatobiliary Systems

In this category, all the outcomes were rated as very low certainty of evidence. The pooled estimate for “gastrointestinal involvement” was 12% (95%CI: 6%–18%, *I* ^2^ = 96.58%; very low certainty of evidence). The funnel plots of “gastrointestinal involvement” and hepatitis showed signs of small‐study effects. However, Egger's test for “gastrointestinal involvement” (*p* = 0.77) and hepatitis (*p* = 0.75) was not statistically significant. In sensitivity analysis with leave‐one‐out method, the pooled estimates for all the outcomes classified in this category were deemed robust.

In subgroup analysis by country, the pooled estimate for “gastrointestinal involvement” was the highest in Kuwait (25%, 95%CI: 21%–39%). The pooled estimate for hepatitis was higher in Turkey (5%, 95%CI: 2%–9%) compared to Iran (less than 1%, 95%CI: 0%–2%).

In subgroup analysis by age, the pooled estimate of “gastrointestinal involvement” was higher in children‐only studies (13%, 95%CI: 2%–31%) compared to adults‐only studies (9%, 95%CI: 1%–22%). However, the pooled estimate of hepatitis was higher in adults‐only studies (5%, 95%CI: 2%–10%) compared to children‐only studies (1%, 95%CI: 0%–3%).

### 3.13 Complications of the Respiratory System

In this category, all the outcomes were rated as very low certainty of evidence. The pooled estimate for pulmonary involvement (5%, 95%CI: 2%–9%, *I* ^2^ = 87.56%; very low certainty of evidence) was the highest. The funnel plots of respiratory system involvement, pneumonia, and pleural effusion depicted signs of small‐study effects. However, Egger's test was not statistically significant for any of the outcomes in this category (*p* > 0.05). In sensitivity analysis with leave‐one‐out method, the pooled estimates for all the outcomes classified in this category were considered to be robust.

In subgroup analysis by country, the pooled estimate for pneumonia was the highest in Saudi Arabia (2%, 95%CI: 1%–4%). Bronchitis (1%, 95%CI: 0%–3%) and pleurisy (2%, 95%CI: 0%–4%) were only reported in Turkey.

In subgroup analysis by age, the pooled estimate of respiratory system involvement was higher in adults‐only studies (2%, 95%CI: 1%–5%) compared to children‐only studies (0%, 95%CI: 0%–2%). However, for pneumonia, the pooled estimate was higher in children‐only studies (2%, 95%CI: 1%–3%) compared to adults‐only studies (1%, 95%CI: 0%–3%).

### 3.14 Complication of the Eye

“Ocular involvement” (very low certainty of evidence) showed evidence of small‐studies effects and Egger's test was statistically significant (*p* = 0.003). In this category, the highest pooled estimates were observed for conjunctivitis (10%, 95%CI: 4%–18%, *I* ^2^ not calculable; very low certainty of evidence) and anterior uveitis (8%, 95%CI: 5%–12%, *I* ^2^ not calculable; very low certainty of evidence). In sensitivity analysis with leave‐one‐out method, the pooled estimates for all the outcomes classified in this category were deemed to be robust. Except for two studies in Kuwait in 1988 and in Saudi Arabia in 2005 that reported “ocular involvement”, all the included studies for the specific complications of the eye were conducted in Turkey and subgroup analysis by country was not feasible. Subgroup analysis to compare the pooled estimates of adults‐only and children‐only studies was not feasible for any of the complications of the eye.

### 3.15 Complications Related to Pregnancy

In this category, all the outcomes except for threatened abortion for which meta‐analysis was not feasible, were rated as very low certainty of evidence. The highest pooled estimate was observed for abortion (20%, 95%CI: 9%–34%, *I* ^2^ = 91.57%; very low certainty of evidence). The funnel plot of abortion showed signs of small‐study effects. However, Egger's test was not statistically significant (*p* = 0.24). In sensitivity analysis with leave‐one‐out method, the pooled estimates for all the outcomes classified in this category were deemed as robust.

In subgroup analysis by country, the pooled estimate for abortion (37%, 95%CI: 30%–45%) and Intrauterine fetal demise (5%, 95%CI: 2%–10%) were the highest in Saudi Arabia. The pooled estimate for preterm labor was the highest in Turkey (7%, 95%CI: 0%–22%).

Subgroup analysis to compare the pooled estimates of adults‐only and children‐only was not feasible for pregnancy complications.

### 3.16 Complications of the Soft Tissue Except Periarticular or Paravertebral Soft Tissue

Only one study reported “soft tissue involvement” (very low certainty of evidence). Meta‐analysis, sensitivity analysis, and subgroup analysis were not feasible for this outcome.

### 3.17 Complications of the Endocrine System Except Genital Glands

We included thyroiditis and thyroid abscess in this category. However, meta‐analysis, sensitivity analysis, and subgroup analysis were not feasible for these outcomes. The certainty of evidence for both of the outcomes were very low.

## 4 Discussion

This systematic review and meta‐analysis aimed to aggregate and synthesize all the available evidence for the relative frequency of the complications of brucellosis in West Asia/Middle East region.

To our knowledge, this is the most comprehensive study on the relative frequency of the complications of brucellosis particularly due to the high number of included studies and the variety of the included complications.

Based on our findings, the pooled estimate of the relative frequency of avascular necrosis of femoral head was less than 1%. Evidence suggests Brucella may act as an under‐recognized ischemic trigger causing osteonecrosis while its diagnosis is often delayed resulting in irreversible joint damage (Konarski et al. 2026).

Based on our findings, the pooled estimate for neurobrucellosis in the West Asia/Middle East region was 3%. However, we noticed that the definition of neurobrucellosis differed among the included studies. For instance, Buzgan defined neurobrucellosis as the central nervous system involvement (Buzgan et al. 2010). This is not recommended as we have shown that brucellosis can also involve the peripheral nervous system. In China, the pooled estimate for central nervous system dysfunction was 5% (95% CI: 3%–10%) (Zheng et al. 2018). This was higher than our pooled estimate for central nervous system involvement (3%). The pooled estimate for hearing loss in the West Asia/Middle East region was 2%. Hearing loss has been associated with a greater risk of relapse or sequalae in patients with neurobrucellosis (Fusetti et al. 2024).

Based on our findings, the pooled estimate of endocarditis was higher in adults‐only studies. This is consistent with the findings of Başaran et al. that reported endocarditis due to brucellosis was more common among younger male patients (Başaran et al. 2025).

We identified three types of infarctions: Putamen infarction, myocardial infarction, and splenic infarction. However, meta‐analysis was not possible due to the fact that only one study assessing each of the aforementioned outcomes was included. Due to the potential severity and fatality of tissue infarction, early diagnosis and prompt treatment are vital for these cases (Keyvanfar et al. 2025).

It is unknown how much of these complications are caused by B. melitensis and other Brucella species and whether the incidence rates of these complications differ based on the causative Brucella species. Therefore, it is suggested to study if the relative frequency of the complications caused by Brucella species is different.

Based on our findings, except for two studies in Kuwait in 1988 and in Saudi Arabia in 2005 reporting “ocular involvement”, all the included studies for the specific complications of the eye were conducted in Turkey. Therefore, we suggest that other countries in the region should also determine the ocular complications among their patient populations. Among ocular complications, conjunctivitis and anterior uveitis were the most common. This finding is consistent with the findings of Evlice et al. where it was reported that among the ocular involvement outcomes, the most common types were uveitis (52.2%) and conjunctivitis (17.6%) (Evlice et al. 2023).

In some complications, the pooled estimate for Kuwait and Saudi Arabia was higher. However, the studies of those subgroups were from the 20th century and early 2000s. Moreover, the findings of subgroup analysis by publication year suggest that orchitis, epididymoorchitis, and meningitis may have decreased over time and that time might influence the relative frequency of these complications. An alternative hypothesis could be the changes in the surveillance systems, diagnostic tools, and the extent of awareness of the complications of brucellosis among patient populations and healthcare workers in the region might have played a role in the influence of time.

Based on our findings, the relative frequency of different complications of brucellosis is different. Therefore, primary studies should take into account these differences in their sample size determination to reach the desired precision. Moreover, the investigators should report their diagnostic criteria for the complications of brucellosis. It is recommended that the criteria used for the determination of the presence of the brucellosis complications be explained in the primary studies.

We limited the number of included outcomes to 254. In future updates of this systematic review, we will increase this number to cover all complications of brucellosis. We performed analyses for all the codes keeping overlapping codes such as “orchitis” and “epididymoorchitis” so that the clinicians using any coding systems could use the findings of this review. However, we advocate the standardization of the reporting of the complications of brucellosis to achieve comparability between studies and across different regions.

Based on the differences of the definitions of neurobrucellosis among the included studies including the restriction of neurobrucellosis to the complications of the central nervous system, it is suspected that the complications of brucellosis are being missed and misclassified when using system‐ and organ‐level outcomes. Moreover, based on our findings, the relative frequencies of the complications of brucellosis are different. Therefore, it is recommended to not document the complications in system‐ or organ‐level formats.

Overall, the certainty of evidence for the majority of the outcomes was rated as very low mostly due the high risk of bias of the included studies. Therefore, these values should be interpreted cautiously as approximate summaries, not exact prevalence rates for all West Asian settings.

### 4.1 Limitations

We were unable to perform other subgroup analyses. We were able to include articles written in English or Persian which could result in missing eligible studies written in other languages. The generalizability of the findings could be affected by the lack of reporting of the complications in all of the countries, risk of bias, and low to very low certainty of evidence.

## 5 Conclusion

In summary, the pooled estimate for endocarditis was higher in adults‐only studies. With the exception of two studies in Kuwait in 1988 and in Saudi Arabia in 2005 reporting “ocular involvement”, all the included studies for the specific complications of the eye were conducted in Turkey. Future studies should take into account the difference in the relative frequency of different complications of brucellosis in their sample size determination and report the diagnostic criteria used for the determination of the complications of brucellosis. The definitions of the complications of brucellosis should be standardized in order to achieve comparability.

## Author Contributions

**Aliasghar Fakhri‐Demeshghieh:** conceptualization, methodology, investigation, formal analysis, writing – original draft. **Elnaz Rostamzaman:** investigation, writing – original draft. **Fatemeh Sayareh:** investigation, writing – original draft. **Amir Nikoukar:** investigation, writing – original draft. **Faranak Farahani:** investigation, writing – original draft. **Farniya Bakhtiary nia:** investigation, writing – original draft. **Helia Sepahvand:** investigation, writing – original draft. **Maryam Khaleghi:** investigation, writing – original draft. **Mohammadsaeed Azamian:** investigation, writing – original draft. **Mohammad Reza Shirzadi:** conceptualization, methodology, writing – review and editing. **Hesameddin Akbarein:** conceptualization, methodology, writing – review and editing. **Vahid Rahmanian:** conceptualization, methodology, investigation, writing – review and editing.

## Funding

The authors have nothing to report.

## Ethics Statement

The protocol of this systematic review was prospectively registered in International prospective register of systematic reviews (PROSPERO) with PROSPERO ID: CRD420251044071. The objectives, inclusion criteria, search strategy, and the number of included outcomes were amended. We used the applicable components of GRADE approach (risk of bias, inconsistency, and imprecision) to assess the certainty of evidence.

## Conflicts of Interest

The authors declare no conflict of interest.

## Supporting information


Supporting File 1

Supporting File 2

Supporting File 3

Supporting File 4

Supporting File 5

Supporting File 6

Supporting File 7

Supporting File 8

Supporting File 9

Supporting File 10

Supporting File 11

## Data Availability

The data and statistical codes used in this systematic review and meta‐analysis are available from the corresponding author upon request.
