# Supplementary material for: Frequency of Human Brucellosis Complications in West Asia: A Systematic Review and Meta‐Analysis
Source: Microbiologyopen. 2026 Sep 7;15(5):e70388. doi: 10.1002/mbo3.70388 (PMC13548425; doi:10.1002/mbo3.70388)
Supplement: Supplementary file 1 [file MBO3-15-e70388-s001.docx]

Table S1. Search strategy algorithms.

|  | Database (date of search) | Search algorithm |
| --- | --- | --- |
| 1 | PubMed (04 March 2024) | ((brucella) OR ("Brucella"[Mesh]) OR (brucellosis) OR ("Brucellosis"[Mesh]) OR (malta fever)) AND (((bahrain[Title/Abstract]) OR ("Bahrain"[Mesh])) OR ((iran[Title/Abstract]) OR ("Iran"[Mesh])) OR ((iraq[Title/Abstract]) OR ("Iraq"[Mesh])) OR ((jordan[Title/Abstract]) OR ("Jordan"[Mesh])) OR ((kuwait[Title/Abstract]) OR ("Kuwait"[Mesh])) OR ((lebanon[Title/Abstract]) OR ("Lebanon"[Mesh])) OR ((oman[Title/Abstract]) OR ("Oman"[Mesh])) OR ((israel[Title/Abstract]) OR ("Israel"[Mesh])) OR (palestine[Title/Abstract]) OR ((qatar[Title/Abstract]) OR ("Qatar"[Mesh])) OR ((saudi arabia[Title/Abstract]) OR ("Saudi Arabia"[Mesh])) OR ((syria[Title/Abstract]) OR ("Syria"[Mesh])) OR ((turkey[Title/Abstract]) OR ("Turkey"[Mesh])) OR ((united arab emirates[Title/Abstract]) OR ("United Arab Emirates"[Mesh])) OR ((yemen[Title/Abstract]) OR ("Yemen"[Mesh]))) |
| 2 | Embase (04 March 2024) | ('brucella'/exp OR 'brucella' OR 'brucellosis'/exp OR 'brucellosis' OR 'malta fever') AND ('bahrain'/exp OR 'bahrain' OR 'iran'/exp OR 'iran' OR 'iraq'/exp OR 'iraq' OR 'jordan'/exp OR 'jordan' OR 'kuwait'/exp OR 'kuwait' OR 'lebanon'/exp OR 'lebanon' OR 'oman'/exp OR 'oman' OR 'israel'/exp OR 'israel' OR 'palestine'/exp OR 'palestine' OR 'qatar'/exp OR 'qatar' OR 'saudi arabia'/exp OR 'saudi arabia' OR 'syrian arab republic'/exp OR 'syrian arab republic' OR 'turkey (republic)'/exp OR 'turkey (republic)' OR 'united arab emirates'/exp OR 'united arab emirates' OR 'yemen'/exp OR 'yemen') |
| 3 | Scopus (04 March 2024) | (TITLE-ABS-KEY(brucella) OR TITLE-ABS-KEY(brucellosis) OR TITLE-ABS-KEY(malta fever)) AND (TITLE-ABS-KEY(bahrain) OR TITLE-ABS-KEY(iran) OR TITLE-ABS-KEY(iraq) OR TITLE-ABS-KEY(jordan) OR TITLE-ABS-KEY(kuwait) OR TITLE-ABS-KEY(lebanon) OR TITLE-ABS-KEY(oman) OR TITLE-ABS-KEY(israel) OR TITLE-ABS-KEY(palestine) OR TITLE-ABS-KEY(qatar) OR TITLE-ABS-KEY(saudi arabia) OR TITLE-ABS-KEY(syria) OR TITLE-ABS-KEY(turkey) OR TITLE-ABS-KEY(united arab emirates) OR TITLE-ABS-KEY(yemen)) |
| 4 | Web of Science (04 March 2024) | (ALL=(brucella) OR ALL=(brucellosis) OR ALL=(malta fever)) AND (ALL=(bahrain) OR ALL=(iran) OR ALL=(iraq) OR ALL=(jordan) OR ALL=(kuwait) OR ALL=(lebanon) OR ALL=(oman) OR ALL=(israel) OR ALL=(palestine) OR ALL=(qatar) OR ALL=(saudi arabia) OR ALL=(syria) OR ALL=(turkey) OR ALL=(united arab emirates) OR ALL=(yemen)) |
| 5 | Proquest (04 March 2024) | noft(brucella OR brucellosis OR malta fever) AND noft(bahrain OR iran OR iraq OR jordan OR kuwait OR lebanon OR oman OR israel OR palestine OR qatar OR saudi arabia OR syria OR turkey OR united arab emirates OR yemen) |
| 6 | Google Scholar (2-4 March 2024) | allintitle: brucella bahrain OR iran OR iraq OR jordan OR kuwait OR lebanon OR oman OR israel OR palestine OR qatar OR saudi OR syria OR turkey OR emirates OR yemen |
|  |  | allintitle: brucellosis bahrain OR iran OR iraq OR jordan OR kuwait OR lebanon OR oman OR israel OR palestine OR qatar OR saudi OR syria OR turkey OR emirates OR yemen |
|  |  | allintitle: malta fever bahrain OR iran OR iraq OR jordan OR kuwait OR lebanon OR oman OR israel OR palestine OR qatar OR saudi OR syria OR turkey OR emirates OR yemen |
