# Supplementary material for: Frequency of Human Brucellosis Complications in West Asia: A Systematic Review and Meta‐Analysis
Source: Microbiologyopen. 2026 Sep 7;15(5):e70388. doi: 10.1002/mbo3.70388 (PMC13548425; doi:10.1002/mbo3.70388)
Supplement: Supplementary file 3 [file MBO3-15-e70388-s003.docx]

Figure S8-1. Musculoskeletal involvement.

Figure S8-2. Skeletal involvement.

Figure S8-3. Osteoarticular involvement.

Figure S8-4. Sacroilitis.

Figure S8-5. Sacroiliitis and sclerosis.

Figure S8-6. Sacroiliitis and Spondylodiscitis.

Figure S8-7. Spondylitis.

Figure S8-8. Spondylodiscitis.

Figure S8-9. Spondylodiscitis and arthritis.

Figure S8-10. Spondylitis and epiphysitis.

Figure S8-11. Spondylitis or spondylodiscitis.

Figure S8-12. Discitis.

Figure S8-13. Spinal brucellosis.

Figure S8-14. Spinal cord involvement (vertebral osteomyelitis).

Figure S8-15. Arthritis or peripheral arthritis.

Figure S8-16. Sternoclavicular arthritis.

Figure S8-17. Arthropathy.

Figure S8-18. Osteomyelitis.

Figure S8-19. Epidural abscess.

Figure S8-20. Vertebral abscess.

Figure S8-21. Paravertebral abscess.

Figure S8-22. Bursitis.

Figure S8-23. Synovitis.

Figure S8-24. Avascular necrosis of femoral head.

Figure S8-25. Genito-urinary system involvement.

Figure S8-26. Urinary system involvement.

Figure S8-27. Orchitis.

Figure S8-28. Epididymitis.

Figure S8-29. Epididymoorchitis.

Figure S8-30. Testis hydrocele.

Figure S8-31. Testicular abscess.

Figure S8-32. Prostatitis.

Figure S8-33. Nephritis.

Figure S8-34. Lymphadenopathy.

Figure S8-35. Lymphadenitis.

Figure S8-36. Lymphadenomegaly.

Figure S8-37. Adenopathy.

Figure S8-38. Parotitis.

Figure S8-39. Spleen abscess.

Figure S8-40. Nervous system involvement.

Figure S8-41. Central nervous system involvement.

Figure S8-42. Peripheral nervous system involvement.

Figure S8-43. Neurobrucellosis.

Figure S8-44. Neuropsychiatric involvement.

Figure S8-45. Psychiatric involvement.

Figure S8-46. Insomnia.

Figure S8-47. Meningitis.

Figure S8-48. Meningoencephalitis.

Figure S8-49. Meningitis or meningoencephalitis.

Figure S8-50. Meningeal irritation findings.

Figure S8-51. Meningovascular involvement.

Figure S8-52. Encephalitis.

Figure S8-53. Myelitis.

Figure S8-54. Subdural hematoma.

Figure S8-55. Sub-arachnoid haemorrhage.

Figure S8-56. Radiculopathy.

Figure S8-57. Polyradiculoneuritis.

Figure S8-58. Peripheral neuritis.

Figure S8-59. Peripheral neuropathy.

Figure S8-60. Brain atrophy.

Figure S8-61. Hydrocephalus.

Figure S8-62. Brain abscess.

Figure S8-63. Cranial nerve involvement.

Figure S8-64. Cranial nerve palsy.

Figure S8-65. Nervus Abducens involvement.

Figure S8-66. Bell’s palsy.

Figure S8-67. Hearing loss.

Figure S8-68. Tinnitus.

Figure S8-69. Papilledema.

Figure S8-70. Paralysis.

Figure S8-71. Ataxia.

Figure S8-72. Depression.

Figure S8-73. Cardiovascular system involvement.

Figure S8-74. Cardiac involvement.

Figure S8-75. Carditis.

Figure S8-76. Endocarditis.

Figure S8-77. Myocarditis.

Figure S8-78. Pericarditis.

Figure S8-79. Thrombophlebitis.

Figure S8-80. Capillary leak syndrome.

Figure S8-81. Hematologic involvement.

Figure S8-82. Leukopenia.

Figure S8-83. Leukocytosis.

Figure S8-84. Lymphopenia.

Figure S8-85. Lymphocytosis.

Figure S8-86. Neutropenia.

Figure S8-87. Neutrophilia.

Figure S8-88. Lymphomonocytosis.

Figure S8-89. Monocytosis.

Figure S8-90. Anemia.

Figure S8-91. Thrombocytopenia.

Figure S8-92. Thrombocytosis.

Figure S8-93. Pancytopenia.

Figure S8-94. Cytopenia.

Figure S8-95. Disseminated intravascular coagulation.

Figure S8-96. Cutaneous involvement.

Figure S8-97. Erythema Nodosum.

Figure S8-98. Petechiae.

Figure S8-99. Petechiae and purpura.

Figure S8-100. Petechial rash.

Figure S8-101. Purpuric rash.

Figure S8-102. Maculopapular eruptions.

Figure S8-103. Gastrointestinal involvement.

Figure S8-104. Hepatobiliary system involvement.

Figure S8-105. Hepatitis.

Figure S8-106. Liver involvement.

Figure S8-107. Liver abscess.

Figure S8-108. Ascites.

Figure S8-109. Cholecystitis.

Figure S8-110. Pancreatitis.

Figure S8-111. Peritonitis.

Figure S8-112. Respiratory system involvement.

Figure S8-113. Pulmonary involvement.

Figure S8-114. Lung involvement.

Figure S8-115. Penumonia.

Figure S8-116. Bronchitis.

Figure S8-117. Pleural effusion.

Figure S8-118. Pleurisy.

Figure S8-119. Ocular involvement.

Figure S8-120. Conjunctivitis.

Figure S8-121. Panuveitis.

Figure S8-122. Dacryoadenitis.

Figure S8-123. Episcleritis.

Figure S8-124. Choroiditis.

Figure S8-125. Abortion.

Figure S8-126. Focal involvement.
