# Supplementary material for: Frequency of Human Brucellosis Complications in West Asia: A Systematic Review and Meta‐Analysis
Source: Microbiologyopen. 2026 Sep 7;15(5):e70388. doi: 10.1002/mbo3.70388 (PMC13548425; doi:10.1002/mbo3.70388)
Supplement: Supplementary file 4 [file MBO3-15-e70388-s004.docx]

Table S10-1. Certainty of evidence (GRADE).

| Complication category | Complication | Risk of Bias | Inconsistency | Imprecision | Certainty of evidence |
| --- | --- | --- | --- | --- | --- |
| Bones, joints, tendons, and paravertebral or periarticular soft tissue | Musculoskeletal involvement | Downgrade | - | Downgrade | Very Low |
|  | Skeletal involvement | Downgrade | Downgrade | Downgrade | Very Low |
|  | Osteoarticular involvement | Downgrade | - | - | Very Low |
|  | Sacroiliitis | Downgrade | - | - | Very Low |
|  | Sacroiliitis and sclerosis | Downgrade | - | - | Very Low |
|  | Sacroiliitis and ankylosis | Downgrade | - | - | Very Low |
|  | Sacroiliitis and spondylodiscitis | Downgrade | - | - | Very Low |
|  | Sacroiliitis and spondylodiscitis and arthritis | Downgrade | - | - | Very Low |
|  | Spondylitis | Downgrade | - | - | Very Low |
|  | Spondylodiscitis | Downgrade | - | - | Very Low |
|  | Spondylodiscitis and arthritis | Downgrade | - | - | Very Low |
|  | Spondylitis and epiphysitis | Downgrade | Downgrade | Downgrade | Very Low |
|  | Spondylitis or sacroillitis | Downgrade | - | - | Very Low |
|  | Spondylitis or spondylodiscitis | Downgrade | Downgrade | - | Very Low |
|  | Discitis | Downgrade | Downgrade | Downgrade | Very Low |
|  | Lumbar abscess | Downgrade | - | - | Very Low |
|  | Subchondral erosion | Downgrade | - | - | Very Low |
|  | Iliac bone involvement | Downgrade | - | - | Very Low |
|  | Spinal brucellosis | Downgrade | - | Downgrade | Very Low |
|  | Extra-spinal skeleton lesions | Downgrade | - | - | Very Low |
|  | Spinal cord involvement (vertebral osteomyelitis) | Downgrade | - | Downgrade | Very Low |
|  | Arthritis or peripheral arthritis | Downgrade | - | - | Very Low |
|  | Arthritis with effusion | Downgrade | - | - | Very Low |
|  | Peripheral joint involvement | Downgrade | - | - | Very Low |
|  | Arthritis and sacroiliitis and spondylodiscitis and periostitis | Downgrade | - | - | Very Low |
|  | Arthritis and spondylodiscitis, and synovitis | Downgrade | - | - | Very Low |
|  | Sternoclavicular arthritis | Downgrade | - | - | Very Low |
|  | Arthropathy | Downgrade | Downgrade | Downgrade | Very Low |
|  | Pauciarticular joint involvement | Downgrade | - | - | Very Low |
|  | Osteomyelitis | Downgrade | - | - | Very Low |
|  | Long-bone osteomyelitis | Downgrade | - | - | Very Low |
|  | Joint effusion | Downgrade | - | - | Very Low |
|  | Interapophysial involvement | Downgrade | - | - | Very Low |
|  | Vertebral Osteomyelitis/discitis | - | - | - | Low |
|  | Epidural abscess | Downgrade | - | - | Very Low |
|  | Vertebral abscess | Downgrade | - | - | Very Low |
|  | Epidural abscess and Psoas muscle abscess | Downgrade | - | - | Very Low |
|  | Iliacus muscle abscess | Downgrade | - | - | Very Low |
|  | Paravertebral abscess | Downgrade | - | - | Very Low |
|  | Paravertebral or Psoas abscess | Downgrade | - | - | Very Low |
|  | Paravertebral or epidural abscess | Downgrade | - | - | Very Low |
|  | Paravertebral soft tissue inflammation | Downgrade | - | - | Very Low |
|  | Bone abscess | Downgrade | - | - | Very Low |
|  | Vertebral osteophyte formation | Downgrade | - | - | Very Low |
|  | Joint de-arrangement | - | - | - | Low |
|  | Periarticular soft tissue involvement | Downgrade | - | - | Very Low |
|  | Bursitis | Downgrade | - | - | Very Low |
|  | Tendinitis | - | - | - | Low |
|  | Tendinitis or bursitis | - | - | - | Low |
|  | Synovitis | Downgrade | - | - | Very Low |
|  | Enthesopathy | Downgrade | - | Downgrade | Very Low |
|  | Enthesitis | Downgrade | - | - | Very Low |
|  | Avascular necrosis of femoral head | Downgrade | - | - | Very Low |
| Genito-urinary system | Genito-urinary system involvement | Downgrade | - | - | Very Low |
|  | Urinary system involvement | Downgrade | - | - | Very Low |
|  | Scrotal involvement | Downgrade | - | - | Very Low |
|  | Orchitis | Downgrade | - | - | Very Low |
|  | Epididymitis | Downgrade | - | - | Very Low |
|  | Epididymoorchitis | Downgrade | - | - | Very Low |
|  | Testis hydrocele | Downgrade | - | - | Very Low |
|  | Testicular abscess | Downgrade | - | - | Very Low |
|  | Varicocele | Downgrade | - | - | Very Low |
|  | Testicular edema | Downgrade | - | - | Very Low |
|  | Prostatitis | Downgrade | - | - | Very Low |
|  | Nephritis | Downgrade | - | - | Very Low |
|  | Nephrotic syndrome | Downgrade | - | - | Very Low |
|  | Renal failure | Downgrade | - | - | Very Low |
|  | Renal abscess | Downgrade | - | - | Very Low |
|  | Kidney involvement | Downgrade | - | - | Very Low |
|  | Aspermia | Downgrade | - | Downgrade | Very Low |
|  | Oligospermia | Downgrade | - | Downgrade | Very Low |
|  | Permanent aspermia or oligospermia | Downgrade | - | Downgrade | Very Low |
| Lymphatic system | Lymphadenopathy | Downgrade | - | - | Very Low |
|  | Neck lymphadenopathy | Downgrade | - | Downgrade | Very Low |
|  | Lymphadenitis | Downgrade | - | - | Very Low |
|  | Lymphadenomegaly | Downgrade | - | - | Very Low |
|  | Adenopathy | Downgrade | Downgrade | Downgrade | Very Low |
|  | Parotitis | Downgrade | - | Downgrade | Very Low |
|  | Parotid abscess | Downgrade | - | - | Very Low |
|  | Spleen abscess | Downgrade | - | - | Very Low |
|  | Splenic infarct | Downgrade | - | - | Very Low |
| Nervous system and psychologic disorders | Nervous system involvement | Downgrade | - | - | Very Low |
|  | Central nervous system involvement | Downgrade | - | - | Very Low |
|  | Peripheral nervous system involvement | Downgrade | Downgrade | - | Very Low |
|  | Central nervous system and peripheral nervous system involvement | Downgrade | - | - | Very Low |
|  | Neurobrucellosis | Downgrade | - | - | Very Low |
|  | Toxic febrile neurobrucellosis | Downgrade | - | - | Very Low |
|  | Neuropsychiatric involvement | Downgrade | - | - | Very Low |
|  | Psychiatric involvement | Downgrade | Downgrade | Downgrade | Very Low |
|  | Insomnia | Downgrade | Downgrade | - | Very Low |
|  | Sleep disturbance | Downgrade | - | Downgrade | Very Low |
|  | Meningitis | Downgrade | - | - | Very Low |
|  | Meningoencephalitis | Downgrade | - | - | Very Low |
|  | Meningitis or meningoencephalitis | Downgrade | - | - | Very Low |
|  | Meningitis or encephalitis | Downgrade | - | - | Very Low |
|  | Meningeal irritation findings | Downgrade | - | - | Very Low |
|  | Meningomyelitis | Downgrade | - | - | Very Low |
|  | Mening ischemic stroke | Downgrade | - | - | Very Low |
|  | Meningovascular involvement | Downgrade | - | - | Very Low |
|  | Meningismus | Downgrade | - | - | Very Low |
|  | Cerebrovascular system involvement | Downgrade | - | - | Very Low |
|  | Encephalitis | Downgrade | - | - | Very Low |
|  | Encephalomyelitis | Downgrade | - | - | Very Low |
|  | Acute cerebellar syndrome | Downgrade | - | - | Very Low |
|  | Cerebellar syndrome and pseudotumor cerebri | Downgrade | - | - | Very Low |
|  | Myelitis | Downgrade | - | - | Very Low |
|  | Intra-cerebral haemorrhage | Downgrade | - | - | Very Low |
|  | Subdural hematoma | Downgrade | - | - | Very Low |
|  | Sub-arachnoid haemorrhage | Downgrade | - | - | Very Low |
|  | Putamen infarction | Downgrade | - | - | Very Low |
|  | Brain or spinal cord paranchymal dysfunction | Downgrade | - | - | Very Low |
|  | Polyneuropathy | Downgrade | Downgrade | - | Very Low |
|  | Radiculopathy | Downgrade | - | - | Very Low |
|  | Polyradiculopathy | Downgrade | - | - | Very Low |
|  | Polyradiculoneuritis | Downgrade | - | - | Very Low |
|  | Peripheral neuritis | Downgrade | - | - | Very Low |
|  | Peripheral neuropathy | Downgrade | Downgrade | - | Very Low |
|  | Axonal sensorimotor neuropathy | Downgrade | - | Downgrade | Very Low |
|  | Optic neuritis | Downgrade | - | - | Very Low |
|  | Superficial sensory loss | Downgrade | - | Downgrade | Very Low |
|  | Deep sensory loss | - | - | - | Low |
|  | Brain atrophy | Downgrade | - | - | Very Low |
|  | Hydrocephalus | Downgrade | - | - | Very Low |
|  | Brain abscess | Downgrade | - | - | Very Low |
|  | Brain demyelination | Downgrade | - | - | Very Low |
|  | Cranial nerve involvement | Downgrade | - | - | Very Low |
|  | Cranial nerve palsy | Downgrade | - | - | Very Low |
|  | Nervus Abducens involvement | Downgrade | - | - | Very Low |
|  | Facial nerve involvement | Downgrade | - | - | Very Low |
|  | Vestibulocochlear nerve involvement | Downgrade | - | - | Very Low |
|  | Facial paralysis | Downgrade | - | - | Very Low |
|  | Bell’s palsy | Downgrade | - | - | Very Low |
|  | Hearing loss | Downgrade | - | - | Very Low |
|  | Tinnitus | Downgrade | - | Downgrade | Very Low |
|  | Papilledema | Downgrade | - | - | Very Low |
|  | Paralysis | Downgrade | - | - | Very Low |
|  | Myelopathy | Downgrade | - | - | Very Low |
|  | Ataxia | Downgrade | - | - | Very Low |
|  | Depression | Downgrade | Downgrade | Downgrade | Very Low |
|  | Major depression | Downgrade | - | - | Very Low |
|  | Depression and/or irritability | Downgrade | - | - | Very Low |
|  | Focal neurological deficits | Downgrade | - | - | Very Low |
|  | Status epilepticus | Downgrade | - | - | Very Low |
|  | Neurological disorder | Downgrade | - | - | Very Low |
| Cardiovascular system | Cardiovascular system involvement | Downgrade | - | - | Very Low |
|  | Cardiac involvement | Downgrade | - | - | Very Low |
|  | Carditis | Downgrade | - | - | Very Low |
|  | Endocarditis | Downgrade | - | - | Very Low |
|  | Endocarditis or vegetation | Downgrade | - | - | Very Low |
|  | Myocarditis | Downgrade | - | - | Very Low |
|  | Myocardial infarction | Downgrade | - | - | Very Low |
|  | Pericarditis | Downgrade | - | - | Very Low |
|  | Perimyocarditis | - | - | - | Low |
|  | Aortitis and aortic rupture | - | - | - | Low |
|  | Aortic thrombus | Downgrade | - | - | Very Low |
|  | Thrombophlebitis | Downgrade | - | - | Very Low |
|  | Septic endophlebitis | Downgrade | - | Downgrade | Very Low |
|  | Endovascular infection | Downgrade | - | - | Very Low |
|  | Deep vein thrombosis | Downgrade | - | - | Very Low |
|  | Capillary leak syndrome | Downgrade | - | - | Very Low |
|  | Sepsis | Downgrade | - | - | Very Low |
|  | Hematologic involvement | Downgrade | - | Downgrade | Very Low |
|  | Leukopenia | Downgrade | - | - | Very Low |
|  | Leukocytosis | Downgrade | - | - | Very Low |
|  | Lymphopenia | Downgrade | - | - | Very Low |
|  | Lymphocytosis | Downgrade | - | Downgrade | Very Low |
|  | Neutropenia | Downgrade | Downgrade | - | Very Low |
|  | Neutrophilia | Downgrade | Downgrade | Downgrade | Very Low |
|  | Eosinophilia | Downgrade | - | - | Very Low |
|  | Lymphomonocytosis | Downgrade | Downgrade | Downgrade | Very Low |
|  | Monocytopenia | Downgrade | - | - | Very Low |
|  | Monocytosis | Downgrade | - | Downgrade | Very Low |
|  | Lymphohistiocytosis | Downgrade | - | - | Very Low |
|  | Anemia | Downgrade | - | - | Very Low |
|  | Hemolytic anemia | Downgrade | - | - | Very Low |
|  | Thrombocytopenia | Downgrade | Downgrade | - | Very Low |
|  | Thrombocytosis | Downgrade | - | - | Very Low |
|  | Pancytopenia | Downgrade | - | - | Very Low |
|  | Cytopenia | Downgrade | - | - | Very Low |
|  | Bicytopenia | Downgrade | - | - | Very Low |
|  | Hemophagocytosis | Downgrade | - | - | Very Low |
|  | Disseminated intravascular coagulation | Downgrade | - | - | Very Low |
|  | Myelodysplastic syndrome | Downgrade | - | - | Very Low |
| Skin | Cutaneous involvement | Downgrade | Downgrade | - | Very Low |
|  | Cellulitis | Downgrade | - | - | Very Low |
|  | Erythema Nodosum | Downgrade | - | - | Very Low |
|  | Erythema Nodosum–like lesions | - | - | - | Low |
|  | Petechiae | Downgrade | - | - | Very Low |
|  | Petechiae and purpura | Downgrade | - | - | Very Low |
|  | Petechial rash | Downgrade | - | - | Very Low |
|  | Purpuric rash | Downgrade | - | - | Very Low |
|  | Maculopapular eruptions | Downgrade | - | - | Very Low |
|  | Psoriasiform lesions | Downgrade | - | - | Very Low |
|  | Malar eruption | Downgrade | - | - | Very Low |
|  | Palmar eczema | - | - | - | Low |
|  | Palmar erythema | Downgrade | - | - | Very Low |
|  | Ecchymosis | Downgrade | - | - | Very Low |
|  | Diffuse rashes | - | - | - | Low |
|  | Pityriasis rosea | - | - | - | Low |
| Gastrointestinal and hepatobiliary systems | Gastrointestinal involvement | Downgrade | - | Downgrade | Very Low |
|  | Hepatobiliary system involvement | Downgrade | - | - | Very Low |
|  | Hepatitis | Downgrade | Downgrade | - | Very Low |
|  | Clinical hepatitis | Downgrade | - | - | Very Low |
|  | Icteric hepatitis | Downgrade | - | - | Very Low |
|  | Anicteric hepatitis | Downgrade | - | - | Very Low |
|  | Liver involvement | Downgrade | - | - | Very Low |
|  | Liver cirrhosis | Downgrade | - | - | Very Low |
|  | Liver abscess | Downgrade | - | - | Very Low |
|  | Ileitis | Downgrade | - | - | Very Low |
|  | Ascites | Downgrade | - | - | Very Low |
|  | Cholecystitis | Downgrade | - | - | Very Low |
|  | Pancreatitis | Downgrade | - | - | Very Low |
|  | Necrotizing pancreatitis | Downgrade | - | - | Very Low |
|  | Oropharyngeal involvement | Downgrade | - | - | Very Low |
|  | Pharyngitis | Downgrade | - | - | Very Low |
|  | Otolaryngology/head and neck involvement | Downgrade | - | Downgrade | Very Low |
|  | Appendicitis | Downgrade | - | - | Very Low |
|  | Peritonitis | Downgrade | - | - | Very Low |
| Respiratory system | Respiratory system involvement | Downgrade | Downgrade | - | Very Low |
|  | Pulmonary involvement | Downgrade | - | - | Very Low |
|  | Lung involvement | Downgrade | - | - | Very Low |
|  | Lung abscess | Downgrade | - | - | Very Low |
|  | Pneumonia | Downgrade | - | - | Very Low |
|  | Bronchitis | Downgrade | - | - | Very Low |
|  | Pneumonitis or bronchiolitis | Downgrade | - | - | Very Low |
|  | Pleural effusion | Downgrade | - | - | Very Low |
|  | Pleurisy | Downgrade | - | - | Very Low |
|  | Pleural involvement | Downgrade | - | - | Very Low |
|  | Emphysema | Downgrade | - | - | Very Low |
|  | Pneumothorax | Downgrade | - | - | Very Low |
| Eye | Ocular involvement | Downgrade | Downgrade | Downgrade | Very Low |
|  | Conjunctivitis | Downgrade | - | Downgrade | Very Low |
|  | Panuveitis | Downgrade | - | - | Very Low |
|  | Uveitis | Downgrade | - | - | Very Low |
|  | Anterior uveitis | Downgrade | Downgrade | - | Very Low |
|  | Posterior uveitis | - | - | - | Low |
|  | Dacryoadenitis | Downgrade | - | - | Very Low |
|  | Episcleritis | Downgrade | - | - | Very Low |
|  | Iridocyclitis | Downgrade | - | - | Very Low |
|  | Choroiditis | Downgrade | - | - | Very Low |
|  | Cystoid macular edema | Downgrade | - | - | Very Low |
|  | Retinitis | Downgrade | - | - | Very Low |
|  | Retinal haemorrhage | Downgrade | - | - | Very Low |
| Pregnancy | Abortion | Downgrade | - | Downgrade | Very Low |
|  | Threatened abortion | - | - | - | Low |
|  | Intrauterine fetal demise | Downgrade | - | - | Very Low |
|  | Preterm labour | Downgrade | - | Downgrade | Very Low |
|  | Infant low birth weight | Downgrade | - | Downgrade | Very Low |
| Soft tissue except periarticular or paravertebral soft tissue | Soft tissue involvement | Downgrade | - | - | Very Low |
| Endocrine system except genital glands | Thyroiditis | Downgrade | - | - | Very Low |
|  | Thyroid abscess | Downgrade | - | - | Very Low |
| Miscellaneous | Visceral abscess | Downgrade | - | - | Very Low |
|  | Local abscess | Downgrade | - | - | Very Low |
|  | Focal involvement | Downgrade | - | Downgrade | Very Low |
