# Supplementary material for: Frequency of Human Brucellosis Complications in West Asia: A Systematic Review and Meta‐Analysis
Source: Microbiologyopen. 2026 Sep 7;15(5):e70388. doi: 10.1002/mbo3.70388 (PMC13548425; doi:10.1002/mbo3.70388)
Supplement: Supplementary file 5 [file MBO3-15-e70388-s005.docx]

Dear Editor-in-chief of MicrobiologyOpen,

We appreciate the comments of reviewer(s) of the manuascript titled “Frequency of human brucellosis complications in West Asia: A systematic review and meta-analysis” with manuscript ID “7679705”. Below is the point-by-point reports of revisions made and responses to the comments of the reviewer(s):

|  | Comment | Response/revision report |
| --- | --- | --- |
| 1 | 1. Relocate Table 2 to the Supplementary Material The inclusion of a 240-row table (Table 2: Care setting and method of diagnosis) directly in the main manuscript severely disrupts the reading flow. Please move this table to the Supplementary files. In its place, provide a brief narrative paragraph in the main text summarizing the data (e.g., the proportion of studies conducted in hospital vs. community settings, and the predominant diagnostic methods used). | In the revision, Table 2 was moved to a new supplementary file (Supplementary file S2).  In the revision, in Page 15, paragraph 1, the number of studies performed in solely hospital settings were added. The number of studies conducted solely in community settings were added.  In the revision, in Page 15, Paragraph 1, it was added that the predominant method of diagnosis was serology techniques and the number of studies reporting the use of serologic methods, bacterial isolation/culture, and polymerase chain reaction (PCR) were added. |
| 2 | 2. Recommend Removal of Trim-and-Fill Adjustments for Prevalence Data In your rebuttal to Comment 4, you justified the retention of the trim-and-fill method by citing the JBI manual. However, applying the trim-and-fill method to single-arm prevalence proportions bounded near zero (0%) is mathematically invalid. Funnel plot asymmetry for rare events is a natural artifact of the boundary effect, not an indicator of publication bias. For example, using this method to impute 26 hypothetical "missing" studies for epididymoorchitis artificially drops the prevalence from 7% to 2% (Results, Page 29; Table S2-2), heavily distorting the epidemiological reality of the region. Please remove the trim-and-fill adjusted estimates entirely from the manuscript. | In the revision, Trim-and-Fill method was removed from Methods and its findings were removed from the manuscript and supplementary files. |
| 3 | 3. Correction of Statistical Justification Regarding Composite Outcomes While I accept your decision to retain the 254 distinct and overlapping outcomes to preserve clinical granularity, the statistical justification provided in your rebuttal is fundamentally incorrect. Combining rare outcomes (e.g., aggregating 1% and 2% prevalences into a 3% composite outcome) does not harmfully inflate variance by artificially pushing p closer to 50%; rather, aggregating sparse events increases statistical power and improves precision. While you do not need to change the 254 outcomes in the manuscript, this statistical misconception should be corrected in your methodological approach to meta-analysis. | This comment is well-appreciated. This mistake was fixed in our approach to meta-analysis. |
| 4 | 4. Inclusion of the Promised Cautious Phrasing In your rebuttal to Comment 5, you agreed to add specific cautious phrasing regarding the generalizability of the pooled estimates (“these values should be interpreted cautiously as approximate summaries...”). However, a review of the revised manuscript shows this phrasing was not actually included; only a generic sentence about generalizability was added to the Limitations paragraph. Please explicitly insert the promised cautionary phrasing into the first paragraph of the Discussion to appropriately temper the headline results given the high I2 values and high risk of bias. | In the revision, in Discussion, we added the missed sentence stating: “Therefore, these values should be interpreted cautiously as approximate summaries, not exact prevalence rates for all West Asian settings.” |
| 5 | 5. Addition of a Visual Summary for Key Complications Because you have opted to relegate the highly overlapping data to the supplementary tables while maintaining all 254 definitions, navigating the overall clinical picture remains challenging for the reader. To greatly improve the manuscript's readability, please add a single summary figure (e.g., a bar chart or summary forest plot) to the main text that visually presents the pooled estimates for only the top 10 most common or clinically significant complications. | In the revision, the bar chart representing the pooled estimates of ten selected complications of brucellosis in West Asia is provided in Figure 2. As there is not consensus on the most important complications of Brucellosis in the literature, the complications in the list were chosen by their severity, mortality, prevalence, and Mandell, Douglas, and Bennett's principles and practice of infectious diseases. |
