# Supplementary material for: Frequency of Human Brucellosis Complications in West Asia: A Systematic Review and Meta‐Analysis
Source: Microbiologyopen. 2026 Sep 7;15(5):e70388. doi: 10.1002/mbo3.70388 (PMC13548425; doi:10.1002/mbo3.70388)
Supplement: Supplementary file 6 [file MBO3-15-e70388-s006.docx]

Figure S6-1. Musculoskeletal involvement.

Figure S6-2. Skeletal involvement.

Figure S6-3. Osteoarticular involvement.

Figure S6-4. Sacroiliitis.

Figure S6-5. Spondylitis.

Figure S6-6. Spondylodiscitis.

Figure S6-7. Spondylitis and epiphysitis.

Figure S6-8. Discitis.

Figure S6-9. Spinal brucellosis.

Figure S6-10. Spinal cord involvement (vertebral osteomyelitis).

Figure S6-11. Arthritis or peripheral arthritis.

Figure S6-12. Sternoclavicular arthritis.

Figure S6-13. Arthropathy.

Figure S6-14. Osteomyelitis.

Figure S6-15. Epidural abscess.

Figure S6-16. Paravertebral abscess.

Figure S6-17. Periarticular soft tissue involvement.

Figure S6-18. Bursitis.

Figure S6-19. Avascular necrosis of femoral head.

Figure S6-20. Genito-urinary system involvement.

Figure S6-21. Urinary system involvement.

Figure S6-22. Orchitis.

Figure S6-23. Epididymoorchitis.

Figure S6-24. Testis hydrocele.

Figure S6-25. Testicular abscess.

Figure S6-26. Prostatitis.

Figure S6-27. Nephritis.

Figure S6-28. Lymphadenopathy.

Figure S6-29. Lymphadenitis.

Figure S6-30. Adenopathy.

Figure S6-31. Spleen abscess.

Figure S6-32. Nervous system involvement.

Figure S6-33. Central nervous system involvement.

Figure S6-34. Neurobrucellosis.

Figure S6-35. Neuropsychiatric involvement.

Figure S6-36. Psychiatric involvement.

Figure S6-37. Meningitis.

Figure S6-38. Meningoencephalitis.

Figure S6-39. Meningitis or meningoencephalitis.

Figure S6-40. Meningeal irritation findings.

Figure S6-41. Encephalitis.

Figure S6-42. Myelitis.

Figure S6-43. Sub-arachnoid haemorrhage.

Figure S6-44. Polyradiculoneuritis.

Figure S6-45. Peripheral neuropathy.

Figure S6-46. Hydrocephalus.

Figure S6-47. Brain abscess.

Figure S6-48. Cranial nerve involvement.

Figure S6-49. Cranial nerve palsy.

Figure S6-50. Nervus Abducens involvement.

Figure S6-51. Hearing loss.

Figure S6-52. Tinnitus.

Figure S6-53. Papilledema.

Figure S6-54. Paralysis.

Figure S6-55. Ataxia.

Figure S6-56. Depression.

Figure S6-57. Cardiovascular system involvement.

Figure S6-58. Cardiac involvement.

Figure S6-59. Carditis.

Figure S6-60. Endocarditis.

Figure S6-61. Myocarditis.

Figure S6-62. Thrombophlebitis.

Figure S6-63. Hematologic involvement.

Figure S6-64. Leukopenia.

Figure S6-65. Leukocytosis.

Figure S6-66. Lymphopenia.

Figure S6-67. Lymphocytosis.

Figure S6-68. Neutropenia.

Figure S6-69. Neutrophilia.

Figure S6-70. Eosinophilia.

Figure S6-71. Lymphomonocytosis.

Figure S6-72. Monocytosis.

Figure S6-73. Anemia.

Figure S6-74. Thrombocytopenia.

Figure S6-75. Thrombocytosis.

Figure S6-76. Pancytopenia.

Figure S6-77. Hemophagocytosis.

Figure S6-78. Disseminated intravascular coagulation.

Figure S6-79. Cutaneous involvement.

Figure S6-80. Erythema Nodosum.

Figure S6-81. Petechiae.

Figure S6-82. Petechiae and purpura.

Figure S6-83. Maculopapular eruptions.

Figure S6-84. Gastrointestinal involvement.

Figure S6-85. Hepatobiliary system involvement.

Figure S6-86. Hepatitis.

Figure S6-87. Liver involvement.

Figure S6-88. Liver abscess.

Figure S6-89. Ascites.

Figure S6-90. Cholecystitis.

Figure S6-91. Pancreatitis.

Figure S6-92. Peritonitis.

Figure S6-93. Respiratory system involvement.

Figure S6-94. Pulmonary involvement.

Figure S6-95. Pneumonia.

Figure S6-96. Pleural effusion.

Figure S6-97. Pleurisy.

Figure S6-98. Ocular involvement.

Figure S6-99. Conjunctivitis.

Figure S6-100. Choroiditis.

Figure S6-101. Abortion.

Figure S6-102. Intrauterine fetal demise.

Figure S6-103. Preterm labour.

Figure S6-104. Infant low birth weight.

Figure S6-105. Focal involvement.
