# Supplementary material for: Frequency of Human Brucellosis Complications in West Asia: A Systematic Review and Meta‐Analysis
Source: Microbiologyopen. 2026 Sep 7;15(5):e70388. doi: 10.1002/mbo3.70388 (PMC13548425; doi:10.1002/mbo3.70388)
Supplement: Supplementary file 7 [file MBO3-15-e70388-s007.docx]

Figure S7-1. Musculoskeletal involvement.

Figure S7-2. Skeletal involvement.

Figure S7-3. Osteoarticular Involvement.

Figure S7-4. Sacroiliitis.

Figure S7-5. Sacroiliitis and sclerosis.

Figure S7-6. Spondylitis.

Figure S7-7. Spondylodiscitis.

Figure S7-8. Sacroiliitis and spondylodiscitis.

Figure S7-9. Spondylodiscitis and arthritis.

Figure S7-10. Spondylitis and epiphysitis.

Figure S7-11. Spondylitis or spondylodiscitis.

Figure S7-12. Discitis.

Figure S7-13. Spinal brucellosis.

Figure S7-14. Spinal cord involvement (vertebral osteomyelitis).

Figure S7-15. Arthritis or peripheral arthritis.

Figure S7-16. Sternoclavicular arthritis.

Figure S7-17. Arthropathy.

Figure S7-18. Osteomyelitis.

Figure S7-19. Joint effusion.

Figure S7-20. Epidural abscess.

Figure S7-21. Vertebral abscess.

Figure S7-22. Paravertebral abscess.

Figure S7-23. Paravertebral or epidural abscess.

Figure S7-24. Periarticular soft tissue involvement.

Figure S7-25. Bursitis.

Figure S7-26. Tendinitis.

Figure S7-27. Synovitis.

Figure S7-28. Avascular necrosis of femoral head.

Figure S7-29. Genito-urinary system involvement.

Figure S7-30. Urinary system involvement.

Figure S7-31. Orchitis.

Figure S7-32. Epididymitis.

Figure S7-33. Epididymoorchitis.

Figure S7-34. Testis hydrocele.

Figure S7-35. Testicular abscess.

Figure S7-36. Testicular edema.

Figure S7-37. Prostatitis.

Figure S7-38. Nephritis.

Figure S7-39. Renal failure.

Figure S7-40. Lymphadenopathy.

Figure S7-41. Lymphadenitis.

Figure S7-42. Lymphadenomegaly.

Figure S7-43. Adenopathy.

Figure S7-44. Parotitis.

Figure S7-45. Spleen abscess.

Figure S7-46. Nervous system involvement.

Figure S7-47. Central nervous system involvement.

Figure S7-48. Peripheral nervous system involvement.

Figure S7-49. Neurobrucellosis.

Figure S7-50. Neuropsychiatric involvement.

Figure S7-51. Psychiatric involvement.

Figure S7-52. Insomnia.

Figure S7-53. Sleep disturbance.

Figure S7-54. Meningitis.

Figure S7-55. Meningoencephalitis.

Figure S7-56. Meningitis or meningoencephalitis.

Figure S7-57. Meningeal irritation findings.

Figure S7-58. Meningovascular involvement.

Figure S7-59. Encephalitis.

Figure S7-60. Myelitis.

Figure S7-61. Intra-cerebral haemorrhage.

Figure S7-62. Subdural hematoma.

Figure S7-63. Sub-arachnoid haemorrhage.

Figure S7-64. Polyneuropathy.

Figure S7-65. Radiculopathy.

Figure S7-66. Polyradiculopathy.

Figure S7-67. Polyradiculoneuritis.

Figure S7-68. Peripheral neuritis.

Figure S7-69. Peripheral neuropathy.

Figure S7-70. Brain atrophy.

Figure S7-71. Hydrocephalus.

Figure S7-72. Brain abscess.

Figure S7-73. Cranial nerve involvement.

Figure S7-74. Cranial nerve palsy.

Figure S7-75. Nervus Abducens involvement.

Figure S7-76. Vestibulocochlear nerve involvement.

Figure S7-77. Facial paralysis.

Figure S7-78. Bell’s palsy.

Figure S7-79. Hearing loss.

Figure S7-80. Tinnitus.

Figure S7-81. Papilledema.

Figure S7-82. Paralysis.

Figure S7-83. Myelopathy.

Figure S7-84. Ataxia.

Figure S7-85. Depression.

Figure S7-86. Status epilepticus.

Figure S7-87. Neurological disorder.

Figure S7-88. Cardiovascular system involvement.

Figure S7-89. Cardiac involvement.

Figure S7-90. Carditis.

Figure S7-91. Endocarditis.

Figure S7-92. Myocarditis.

Figure S7-93. Pericarditis.

Figure S7-94. Thrombophlebitis.

Figure S7-95. Deep vein thrombosis.

Figure S7-96. Capillary leak syndrome.

Figure S7-97. Hematologic involvement.

Figure S7-98. Leukopenia.

Figure S7-99. Leukocytosis.

Figure S7-100. Lymphopenia.

Figure S7-101. Lymphocytosis.

Figure S7-102. Neutropenia.

Figure S7-103. Neutrophilia.

Figure S7-104. Eosinophilia.

Figure S7-105. Lymphomonocytosis.

Figure S7-106. Monocytosis.

Figure S7-107. Anemia.

Figure S7-108. Thrombocytopenia.

Figure S7-109. Thrombocytosis.

Figure S7-110. Pancytopenia.

Figure S7-111. Cytopenia.

Figure S7-112. Hemophagocytosis.

Figure S7-113. Disseminated intravascular coagulation.

Figure S7-114. Cutaneous involvement.

Figure S7-115. Erythema Nodosum.

Figure S7-116. Petechiae.

Figure S7-117. Petechiae and purpura.

Figure S7-118. Petechial rash.

Figure S7-119. Purpuric rash.

Figure S7-120. Maculopapular eruptions.

Figure S7-121. Psoriasiform lesions.

Figure S7-122. Malar eruption.

Figure S7-123. Palmar erythema.

Figure S7-124. Gastrointestinal involvement.

Figure S7-125. Hepatobiliary system involvement.

Figure S7-126. Hepatitis.

Figure S7-127. Liver involvement.

Figure S7-128. Liver abscess.

Figure S7-129. Ascites.

Figure S7-130. Cholecystitis.

Figure S7-131. Pancreatitis.

Figure S7-132. Peritonitis.

Figure S7-133. Respiratory system involvement.

Figure S7-134. Pulmonary involvement.

Figure S7-135. Lung involvement.

Figure S7-136. Lung abscess.

Figure S7-137. Pneumonia.

Figure S7-138. Bronchitis.

Figure S7-139. Pleural effusion.

Figure S7-140. Pleurisy.

Figure S7-141. Ocular involvement.

Figure S7-142. Conjunctivitis.

Figure S7-143. Panuveitis.

Figure S7-144. Anterior uveitis.

Figure S7-145. Dacryoadenitis.

Figure S7-146. Episcleritis.

Figure S7-147. Choroiditis.

Figure S7-148. Abortion.

Figure S7-149. Intrauterine fetal demise.

Figure S7-150. Preterm labour.

Figure S7-151. Infant low birth weight.

Figure S7-152. Focal involvement.
