# Supplementary material for: Frequency of Human Brucellosis Complications in West Asia: A Systematic Review and Meta‐Analysis
Source: Microbiologyopen. 2026 Sep 7;15(5):e70388. doi: 10.1002/mbo3.70388 (PMC13548425; doi:10.1002/mbo3.70388)
Supplement: Supplementary file 8 [file MBO3-15-e70388-s008.docx]

Table S3-1. Summary of the pooled estimates and the point estimates of the complications of brucellosis in West Asia.

| Complication category | Complication | Pooled estimate/ point estimate | 95%CI | I-squared | Meta-analysis performed |
| --- | --- | --- | --- | --- | --- |
| Bones, joints, tendons, and paravertebral or periarticular soft tissue | Musculoskeletal involvement | 0.50 | 0.39-0.61 | 96.53% | Yes |
|  | Skeletal involvement | 0.47 | 0.32-0.62 | 96.16% | Yes |
|  | Osteoarticular involvement | 0.36 | 0.31-0.40 | 95.32% | Yes |
|  | Sacroiliitis | 0.13 | 0.11-0.16 | 96.56% | Yes |
|  | Sacroiliitis and sclerosis | 0.03 | 0.02 -0.05 | - | Yes |
|  | Sacroiliitis and ankylosis | 0.02 | 0.01-0.04 | - | Not feasible |
|  | Sacroiliitis and spondylodiscitis | 0.03 | 0.01-0.05 | - | Yes |
|  | Sacroiliitis and spondylodiscitis and arthritis | 0.01 | 0.00-0.04 | - | Not feasible |
|  | Spondylitis | 0.08 | 0.06-0.11 | 95.43% | Yes |
|  | Spondylodiscitis | 0.14 | 0.11-0.18 | 92.05% | Yes |
|  | Spondylodiscitis and arthritis | 0.02 | 0.01-0.04 | - | Yes |
|  | Spondylitis and epiphysitis | 0.07 | 0.00-0.30 | - | Yes |
|  | Spondylitis or sacroillitis | 0.08 | 0.06-0.12 | - | Not feasible |
|  | Spondylitis or spondylodiscitis | 0.16 | 0.13-0.20 | - | Yes |
|  | Discitis | 0.07 | 0.02-0.17 | 88.60% | Yes |
|  | Lumbar abscess | 0.07 | 0.04-0.11 | - | Not feasible |
|  | Subchondral erosion | 0.07 | 0.04-0.11 | - | Not feasible |
|  | Iliac bone involvement | 0.02 | 0.00-0.10 | - | Not feasible |
|  | Spinal brucellosis | 0.10 | 0.05-0.18 | 94.46% | Yes |
|  | Extra-spinal skeleton lesions | 0.18 | 0.14-0.23 | - | Not feasible |
|  | Spinal cord involvement (vertebral osteomyelitis) | 0.04 | 0.01-0.12 | - | Yes |
|  | Arthritis or peripheral arthritis | 0.20 | 0.17-0.23 | 97.70% | Yes |
|  | Arthritis with effusion | 0.08 | 0.06-0.11 | - | Not feasible |
|  | Peripheral joint involvement | 0.03 | 0.02-0.05 | - | Not feasible |
|  | Arthritis and sacroiliitis and spondylodiscitis and periostitis | 0.01 | 0.00-0.03 | - | Not feasible |
|  | Arthritis and spondylodiscitis, and synovitis | 0.01 | 0.00-0.03 | - | Not feasible |
|  | Sternoclavicular arthritis | 0.01 | 0.00-0.01 | 35.00% | Yes |
|  | Arthropathy | 0.39 | 0.24-0.56 | - | Yes |
|  | Pauciarticular joint involvement | 0.01 | 0.00-0.03 | - | Not feasible |
|  | Osteomyelitis | 0.02 | 0.01-0.03 | 85.70% | Yes |
|  | Long-bone osteomyelitis | 0.01 | 0.00-0.03 | - | Not feasible |
|  | Joint effusion | 0.10 | 0.08-0.12 | - | Yes |
|  | Interapophysial involvement | 0.03 | 0.01-0.06 | - | Not feasible |
|  | Vertebral osteomyelitis/discitis | 0.11 | 0.08-0.16 | - | Not feasible |
|  | Epidural abscess | 0.03 | 0.01-0.05 | 63.22% | Yes |
|  | Vertebral abscess | 0.04 | 0.02-0.05 | - | Yes |
|  | Epidural abscess and Psoas muscle abscess | <0.01 | 0.00-0.02 | - | Not feasible |
|  | Iliacus muscle abscess | <0.01 | 0.00-0.02 | - | Not feasible |
|  | Paravertebral abscess | 0.01 | 0.00-0.02 | 60.23% | Yes |
|  | Paravertebral or Psoas abscess | 0.02 | 0.01-0.06 | - | Not feasible |
|  | Paravertebral or epidural abscess | 0.04 | 0.03-0.06 | - | Yes |
|  | Paravertebral soft tissue inflammation | 0.02 | 0.00-0.05 | - | Not feasible |
|  | Bone abscess | 0.02 | 0.01-0.05 | - | Not feasible |
|  | Vertebral osteophyte formation | 0.03 | 0.01-0.06 | - | Not feasible |
|  | Joint de-arrangement | 0.04 | 0.02-0.07 | - | Not feasible |
|  | Periarticular soft tissue involvement | 0.02 | 0.01-0.04 | - | Yes |
|  | Bursitis | 0.01 | 0.01-0.02 | 69.90% | Yes |
|  | Tendinitis | 0.01 | 0.00-0.02 | - | Yes |
|  | Tendinitis or bursitis | 0.00 | 0.00-0.01 | - | Not feasible |
|  | Synovitis | 0.01 | 0.00-0.02 | - | Yes |
|  | Enthesopathy | 0.04 | 0.00-0.11 | - | Not feasible |
|  | Enthesitis | 0.01 | 0.00-0.02 | - | Not feasible |
|  | Avascular necrosis of femoral head | <0.01 | 0.00-0.01 | - | Yes |
| Genito-urinary system | Genito-urinary system involvement | 0.05 | 0.03-0.06 | 75.15% | Yes |
|  | Urinary system involvement | 0.02 | 0.00-0.05 | - | Yes |
|  | Scrotal involvement | 0.11 | 0.07-0.15 | - | Not feasible |
|  | Orchitis | 0.05 | 0.03-0.07 | 93.67% | Yes |
|  | Epididymitis | 0.01 | 0.00-0.01 | - | Yes |
|  | Epididymoorchitis | 0.07 | 0.06-0.09 | 90.94% | Yes |
|  | Testis hydrocele | 0.02 | 0.00-0.07 | 91.44% | Yes |
|  | Testicular abscess | 0.01 | 0.00-0.02 | 89.03% | Yes |
|  | Varicocele | <0.01 | 0.00-0.00 | - | Not feasible |
|  | Testicular edema | 0.04 | 0.02-0.07 | - | Yes |
|  | Prostatitis | 0.01 | 0.00-0.02 | 50.13% | Yes |
|  | Nephritis | <0.01 | 0.00-0.01 | 42.42% | Yes |
|  | Nephrotic syndrome | <0.01 | 0.00-0.01 | - | Not feasible |
|  | Renal failure | <0.01 | 0.00-0.00 | - | Yes |
|  | Renal abscess | <0.01 | 0.00-0.02 | - | Not feasible |
|  | Kidney involvement | <0.01 | 0.00-0.01 | - | Not feasible |
|  | Aspermia | 0.36 | 0.12-0.63 | - | Not feasible |
|  | Oligospermia | 0.57 | 0.30-0.82 | - | Not feasible |
|  | Permanent aspermia or oligospermia | 0.29 | 0.07-0.55 | - | Not feasible |
| Lymphatic system | Lymphadenopathy | 0.09 | 0.07-0.10 | 91.89% | Yes |
|  | Neck lymphadenopathy | 0.04 | 0.00-0.11 | - | Not feasible |
|  | Lymphadenitis | 0.06 | 0.02-0.12 | 57.36% | Yes |
|  | Lymphadenomegaly | 0.07 | 0.04-0.11 | - | Yes |
|  | Adenopathy | 0.07 | 0.00-0.22 | 99.01% | Yes |
|  | Parotitis | <0.01 | 0.00-0.01 | - | Yes |
|  | Parotid abscess | 0.01 | 0.00-0.03 | - | Not feasible |
|  | Spleen abscess | 0.01 | 0.00-0.02 | 61.98% | Yes |
|  | Splenic infarct | 0.01 | 0.00-0.04 | - | Not feasible |
| Nervous system and psychologic disorders | Nervous system involvement | 0.06 | 0.04-0.08 | 86.27% | Yes |
|  | Central nervous system involvement | 0.03 | 0.01-0.05 | 89.83% | Yes |
|  | Peripheral nervous system involvement | 0.02 | 0.01-0.04 | - | Yes |
|  | Central nervous system and peripheral nervous system involvement | 0.01 | 0.00-0.02 | - | Not feasible |
|  | Neurobrucellosis | 0.03 | 0.02-0.04 | 86.18% | Yes |
|  | Toxic febrile neurobrucellosis | <0.01 | 0.00-0.01 | - | Not feasible |
|  | Neuropsychiatric involvement | 0.04 | 0.02-0.07 | 83.11% | Yes |
|  | Psychiatric involvement | 0.09 | 0.01-0.23 | 97.23% | Yes |
|  | Insomnia | 0.13 | 0.10-0.16 | - | Yes |
|  | Sleep disturbance | 0.43 | 0.35-0.52 | - | Yes |
|  | Meningitis | 0.03 | 0.02-0.03 | 80.26% | Yes |
|  | Meningoencephalitis | 0.01 | 0.00-0.02 | 68.82% | Yes |
|  | Meningitis or meningoencephalitis | 0.04 | 0.03-0.05 | 0.00% | Yes |
|  | Meningitis or encephalitis | 0.02 | 0.01-0.03 | - | Not feasible |
|  | Meningeal irritation findings | 0.03 | 0.02-0.05 | - | Yes |
|  | Meningomyelitis | <0.01 | 0.00-0.01 | - | Not feasible |
|  | Mening ischemic stroke | 0.01 | 0.00-0.02 |  | Not feasible |
|  | Meningovascular involvement | 0.01 | 0.00-0.01 | - | Yes |
|  | Meningismus | <0.01 | 0.00-0.02 | - | Not feasible |
|  | Cerebrovascular system involvement | 0.04 | 0.01-0.11 | - | Not feasible |
|  | Encephalitis | 0.01 | 0.00-0.01 | 0.00% | Yes |
|  | Encephalomyelitis | 0.01 | 0.00-0.02 | - | Not feasible |
|  | Acute cerebellar syndrome | <0.01 | 0.00-0.01 | - | Not feasible |
|  | Cerebellar syndrome and pseudotumor cerebri | <0.01 | 0.00-0.01 | - | Not feasible |
|  | Myelitis | <0.01 | 0.00-0.01 | 33.08% | Yes |
|  | Intra-cerebral haemorrhage | <0.01 | 0.00-0.00 | - | Yes |
|  | Subdural hematoma | <0.01 | 0.00-0.01 | - | Yes |
|  | Sub-arachnoid haemorrhage | <0.01 | 0.00-0.01 | - | Yes |
|  | Putamen infarction | 0.01 | 0.00-0.02 | - | Not feasible |
|  | Brain or spinal cord paranchymal dysfunction | 0.01 | 0.00-0.02 | - | Not feasible |
|  | Polyneuropathy | 0.02 | 0.01-0.04 | - | Yes |
|  | Radiculopathy | <0.01 | 0.00-0.02 | - | Yes |
|  | Polyradiculopathy | <0.01 | 0.00-0.00 | - | Yes |
|  | Polyradiculoneuritis | <0.01 | 0.00-0.01 | 0.00% | Yes |
|  | Peripheral neuritis | <0.01 | 0.00-0.01 | - | Yes |
|  | Peripheral neuropathy | 0.02 | 0.00-0.07 | 91.06% | Yes |
|  | Axonal sensorimotor neuropathy | 0.32 | 0.18-0.47 | - | Not feasible |
|  | Optic neuritis | 0.01 | 0.00-0.02 | - | Not feasible |
|  | Superficial sensory loss | 0.11 | 0.04-0.20 | - | Not feasible |
|  | Deep sensory loss | 0.00 | 0.00-0.03 | - | Not feasible |
|  | Brain atrophy | 0.01 | 0.00-0.02 | - | Yes |
|  | Hydrocephalus | <0.01 | 0.00-0.01 | - | Yes |
|  | Brain abscess | 0.01 | 0.00-0.02 | 71.06% | Yes |
|  | Brain demyelination | <0.01 | 0.00-0.01 | - | Not feasible |
|  | Cranial nerve involvement | 0.02 | 0.01-0.03 | 44.26% | Yes |
|  | Cranial nerve palsy | 0.01 | 0.00-0.02 | - | Yes |
|  | Nervus Abducens involvement | 0.01 | 0.00-0.01 | 37.15% | Yes |
|  | Facial nerve involvement | 0.02 | 0.00-0.05 | - | Not feasible |
|  | Vestibulocochlear nerve involvement | 0.03 | 0.01-0.06 | - | Yes |
|  | Facial paralysis | 0.01 | 0.00-0.04 | - | Yes |
|  | Bell’s palsy | <0.01 | 0.00-0.01 | - | Yes |
|  | Hearing loss | 0.02 | 0.00-0.04 | 86.50% | Yes |
|  | Tinnitus | 0.10 | 0.00-0.39 | - | Yes |
|  | Papilledema | 0.01 | 0.00-0.02 | 72.58% | Yes |
|  | Paralysis | 0.01 | 0.00-0.02 | 79.69% | Yes |
|  | Myelopathy | <0.01 | 0.00-0.00 | - | Yes |
|  | Ataxia | <0.01 | 0.00-0.01 | 42.74% | Yes |
|  | Depression | 0.07 | 0.02-0.16 | 99.23% | Yes |
|  | Major depression | 0.01 | 0.00-0.03 | - | Not feasible |
|  | Depression and/or irritability | 0.10 | 0.06-0.16 | - | Not feasible |
|  | Focal neurological deficits | 0.04 | 0.02-0.07 | - | Not feasible |
|  | Status epilepticus | <0.01 | 0.00-0.00 | - | Yes |
|  | Neurological disorder | 0.04 | 0.03-0.06 | - | Yes |
| Cardiovascular system | Cardiovascular system involvement | 0.01 | 0.00-0.01 | 0.00% | Yes |
|  | Cardiac involvement | <0.01 | 0.00-0.01 | 0.00% | Yes |
|  | Carditis | 0.01 | 0.00-0.03 | - | Yes |
|  | Endocarditis | 0.01 | 0.00-0.01 | 39.61% | Yes |
|  | Endocarditis or vegetation | 0.01 | 0.00-0.03 | - | Not feasible |
|  | Myocarditis | <0.01 | 0.00-0.01 | 12.74% | Yes |
|  | Myocardial infarction | <0.01 | 0.00-0.01 | - | Not feasible |
|  | Pericarditis | 0.01 | 0.00-0.02 | - | Yes |
|  | Perimyocarditis | <0.01 | 0.00-0.02 | - | Not feasible |
|  | Aortitis and aortic rupture | <0.01 | 0.00-0.02 | - | Not feasible |
|  | Aortic thrombus | 0.01 | 0.00-0.02 | - | Not feasible |
|  | Thrombophlebitis | <0.01 | 0.00-0.01 | 22.66% | Yes |
|  | Septic endophlebitis | 0.14 | 0.00-0.52 | - | Not feasible |
|  | Endovascular infection | 0.00 | 0.00-0.01 | - | Not feasible |
|  | Deep vein thrombosis | <0.01 | 0.00-0.01 | - | Yes |
|  | Capillary leak syndrome | <0.01 | 0.00-0.01 | - | Yes |
|  | Sepsis | 0.01 | 0.00-0.03 | - | Not feasible |
|  | Hematologic involvement | 0.25 | 0.16-0.34 | 98.37% | Yes |
|  | Leukopenia | 0.14 | 0.12-0.16 | 92.85% | Yes |
|  | Leukocytosis | 0.09 | 0.08-0.11 | 81.44% | Yes |
|  | Lymphopenia | 0.09 | 0.04-0.14 | 93.27% | Yes |
|  | Lymphocytosis | 0.32 | 0.17-0.48 | 98.69% | Yes |
|  | Neutropenia | 0.13 | 0.08-0.18 | 91.61% | Yes |
|  | Neutrophilia | 0.19 | 0.02-0.46 | 98.77% | Yes |
|  | Eosinophilia | 0.04 | 0.01-0.10 | - | Yes |
|  | Lymphomonocytosis | 0.37 | 0.20-0.57 | 98.67% | Yes |
|  | Monocytopenia | 0.04 | 0.01-0.07 | - | Not feasible |
|  | Monocytosis | 0.18 | 0.09-0.29 | - | Yes |
|  | Lymphohistiocytosis | 0.01 | 0.01-0.03 | - | Not feasible |
|  | Anemia | 0.35 | 0.31-0.39 | 96.39% | Yes |
|  | Hemolytic anemia | <0.01 | 0.00-0.02 | - | Not feasible |
|  | Thrombocytopenia | 0.11 | 0.08-0.13 | 96.28% | Yes |
|  | Thrombocytosis | 0.05 | 0.03-0.07 | 77.46% | Yes |
|  | Pancytopenia | 0.05 | 0.04-0.06 | 80.76% | Yes |
|  | Cytopenia | 0.34 | 0.30-0.38 | - | Yes |
|  | Bicytopenia | 0.04 | 0.01-0.11 | - | Not feasible |
|  | Hemophagocytosis | 0.02 | 0.01-0.02 | 5.06% | Yes |
|  | Disseminated intravascular coagulation | <0.01 | 0.00-0.01 | 27.40% | Yes |
|  | Myelodysplastic syndrome | 0.01 | 0.00-0.04 | - | Not feasible |
| Skin | Cutaneous involvement | 0.04 | 0.03-0.06 | 91.90% | Yes |
|  | Cellulitis | <0.01 | 0.00-0.01 | - | Not feasible |
|  | Erythema Nodosum | <0.01 | 0.00-0.01 | 40.63% | Yes |
|  | Erythema Nodosum–like lesions | <0.01 | 0.00-0.04 | - | Not feasible |
|  | Petechiae | <0.01 | 0.00-0.01 | 51.93% | Yes |
|  | Petechiae and purpura | 0.03 | 0.00-0.07 | 76.65% | Yes |
|  | Petechial rash | 0.01 | 0.00-0.02 | - | Yes |
|  | Purpuric rash | 0.01 | 0.00-0.02 | - | Yes |
|  | Maculopapular eruptions | 0.01 | 0.00-0.01 | 51.41% | Yes |
|  | Psoriasiform lesions | 0.01 | 0.00-0.03 | - | Yes |
|  | Malar eruption | 0.01 | 0.00-0.03 | - | Yes |
|  | Palmar eczema | 0.01 | 0.00-0.03 | - | Not feasible |
|  | Palmar erythema | 0.01 | 0.00-0.03 | - | Yes |
|  | Ecchymosis | 0.01 | 0.00-0.02 | - | Not feasible |
|  | Diffuse rashes | <0.01 | 0.00-0.01 | - | Not feasible |
|  | Pityriasis rosea | <0.01 | 0.00-0.01 | - | Not feasible |
| Gastrointestinal and hepatobiliary systems | Gastrointestinal involvement | 0.12 | 0.06-0.18 | 96.58% | Yes |
|  | Hepatobiliary system involvement | 0.26 | 0.23-0.30 | - | Yes |
|  | Hepatitis | 0.04 | 0.02-0.08 | 95.66% | Yes |
|  | Clinical hepatitis | 0.01 | 0.00-0.03 | - | Not feasible |
|  | Icteric hepatitis | <0.01 | 0.00-0.01 | - | Not feasible |
|  | Anicteric hepatitis | 0.38 | 0.34-0.43 | - | Not feasible |
|  | Liver involvement | 0.02 | 0.01-0.04 | - | Yes |
|  | Liver cirrhosis | 0.01 | 0.00-0.04 | - | Not feasible |
|  | Liver abscess | <0.01 | 0.00-0.01 | 27.80% | Yes |
|  | Ileitis | 0.01 | 0.00-0.02 | - | Not feasible |
|  | Ascites | 0.02 | 0.00-0.05 | 85.24% | Yes |
|  | Cholecystitis | 0.01 | 0.00-0.03 | 78.45% | Yes |
|  | Pancreatitis | 0.01 | 0.00-0.05 | 94.04% | Yes |
|  | Necrotizing pancreatitis | 0.01 | 0.00-0.02 | - | Not feasible |
|  | Oropharyngeal involvement | 0.11 | 0.08-0.14 | - | Not feasible |
|  | Pharyngitis | 0.08 | 0.04-0.13 | - | Not feasible |
|  | Otolaryngology/head and neck involvement | 0.05 | 0.01-0.13 | - | Not feasible |
|  | Appendicitis | 0.01 | 0.00-0.03 | - | Not feasible |
|  | Peritonitis | <0.01 | 0.00-0.01 | 0.00% | Yes |
| Respiratory system | Respiratory system involvement | 0.03 | 0.01-0.07 | 94.11% | Yes |
|  | Pulmonary involvement | 0.05 | 0.02-0.09 | 87.56% | Yes |
|  | Lung involvement | 0.01 | 0.00-0.01 | - | Yes |
|  | Lung abscess | 0.01 | 0.00-0.03 | - | Yes |
|  | Pneumonia | 0.01 | 0.00-0.02 | 46.46% | Yes |
|  | Bronchitis | 0.01 | 0.00-0.03 | - | Yes |
|  | Pneumonitis or bronchiolitis | 0.04 | 0.02-0.05 | - | Not feasible |
|  | Pleural effusion | 0.01 | 0.00-0.02 | 64.94% | Yes |
|  | Pleurisy | 0.02 | 0.00-0.04 | 80.90% | Yes |
|  | Pleural involvement | 0.02 | 0.00-0.05 | - | Not feasible |
|  | Emphysema | 0.01 | 0.00-0.05 | - | Not feasible |
|  | Pneumothorax | 0.01 | 0.00-0.03 | - | Not feasible |
| Eye | Ocular involvement | 0.05 | 0.01-0.12 | 96.62% | Yes |
|  | Conjunctivitis | 0.10 | 0.04-0.18 | - | Yes |
|  | Panuveitis | 0.02 | 0.01-0.05 | - | Yes |
|  | Uveitis | 0.03 | 0.00-0.06 | - | Not feasible |
|  | Anterior uveitis | 0.08 | 0.05-0.12 | - | Yes |
|  | Posterior uveitis | 0.01 | 0.00-0.03 | - | Not feasible |
|  | Dacryoadenitis | 0.01 | 0.00-0.03 | - | Yes |
|  | Episcleritis | 0.02 | 0.01-0.04 | - | Yes |
|  | Iridocyclitis | 0.01 | 0.00-0.03 | - | Not feasible |
|  | Choroiditis | 0.03 | 0.00-0.10 | - | Yes |
|  | Cystoid macular edema | 0.02 | 0.00-0.05 | - | Not feasible |
|  | Retinitis | <0.01 | 0.00-0.00 | - | Not feasible |
|  | Retinal haemorrhage | 0.00 | 0.00-0.02 | - | Not feasible |
| Pregnancy | Abortion | 0.20 | 0.09-0.34 | 91.57% | Yes |
|  | Threatened abortion | 0.04 | 0.02-0.07 | - | Not feasible |
|  | Intrauterine fetal demise | 0.03 | 0.01-0.07 | 59.36% | Yes |
|  | Preterm labour | 0.06 | 0.02-0.13 | 82.95% | Yes |
|  | Infant low birth weight | 0.12 | 0.05-0.21 | - | Yes |
| Soft tissue except periarticular or paravertebral soft tissue | Soft tissue involvement | <0.01 | 0.00-0.01 | - | Not feasible |
| Endocrine system except genital glands | Thyroiditis | <0.01 | 0.00-0.00 | - | Not feasible |
|  | Thyroid abscess | 0.02 | 0.00-0.08 | - | Not feasible |
| Miscellaneous | Visceral abscess | 0.05 | 0.03-0.08 | - | Not feasible |
|  | Local abscess | 0.02 | 0.00-0.06 | - | Not feasible |
|  | Focal involvement | 0.32 | 0.24-0.40 | 94.32% | Yes |
