# Supplementary material for: Frequency of Human Brucellosis Complications in West Asia: A Systematic Review and Meta‐Analysis
Source: Microbiologyopen. 2026 Sep 7;15(5):e70388. doi: 10.1002/mbo3.70388 (PMC13548425; doi:10.1002/mbo3.70388)
Supplement: Supplementary file 9 [file MBO3-15-e70388-s009.docx]

Figure S5-1. Musculoskeletal involvement.

Figure S5-2. Osteoarticular involvement.

Figure S5-3. Sacroiliitis.

Figure S5-4. Spondylitis.

Figure S5-5. Spondylodiscitis.

Figure S5-6. Spinal brucellosis.

Figure S5-7. Arthritis or peripheral arthritis.

Figure S5-8. Osteomyelitis.

Figure S5-9. Bursitis.

Figure S5-10. Genito-urinary system involvement.

Figure S5-11. Orchitis.

Figure S5-12. Epididymoorchitis.

Figure S5-13. Lymphadenopathy.

Figure S5-14. Nervous system involvement.

Figure S5-15. Central nervous system involvement.

Figure S5-16. Neurobrucellosis.

Figure S5-17. Neuropsychiatric involvement.

Figure S5-18. Meningitis.

Figure S5-19. Cranial nerve involvement.

Figure S5-20. Hearing loss.

Figure S5-21. Depression.

Figure S5-22. Cardiovascular system involvement.

Figure S5-23. Endocarditis.

Figure S5-24. Hematologic involvement.

Figure S5-25. Leukopenia.

Figure S5-26. Leukocytosis.

Figure S5-27. Lymphocytosis.

Figure S5-28. Neutropenia.

Figure S5-29. Anemia.

Figure S5-30. Thrombocytopenia.

Figure S5-31. Thrombocytosis.

Figure S5-32. Pancytopenia.

Figure S5-33. Cutaneous involvement.

Figure S5-34. Gastrointestinal involvement.

Figure S5-35. Hepatitis.

Figure S5-36. Respiratory system involvement.

Figure S5-37. Pneumonia.

Figure S5-38. Pleural effusion.

Figure S5-39. Ocular involvement.

Figure S5-40. Abortion.
