# Supplementary material for: Frequency of Human Brucellosis Complications in West Asia: A Systematic Review and Meta‐Analysis
Source: Microbiologyopen. 2026 Sep 7;15(5):e70388. doi: 10.1002/mbo3.70388 (PMC13548425; doi:10.1002/mbo3.70388)
Supplement: Supplementary file 10 [file MBO3-15-e70388-s010.docx]

A) Study-based summary of risk of bias in the included studies:

Table S4-1. Study-based summary of risk of bias assessment of the included studies.

|  | Study ID | Q1 | Q2 | Q3 | Q4 | Q5 | Q6 | Q7 | Q8 | Q9 |
| --- | --- | --- | --- | --- | --- | --- | --- | --- | --- | --- |
| 1 | Abbasi 2020 | Yes | Yes | Yes | Yes | Yes | Not for all outcomes | Not for all outcomes | Yes | Yes |
| 2 | Abd Alrheam 2015 | Yes | Yes | Yes | Yes | Yes | Not for all outcomes | Not for all outcomes | Yes | Yes |
| 3 | Abdi-Liae 2007 | Yes | Yes | No | Yes | Yes | Not for all outcomes | Not for all outcomes | Yes | Yes |
| 4 | Afify 2013 | No | Yes | No | Yes | Yes | Yes | Yes | Yes | Yes |
| 5 | Akbayram 2011 | No | Yes | Yes | No | Yes | Yes | Yes | Yes | Yes |
| 6 | Akbayram 2015 | No | Yes | Yes | Yes | Yes | Yes | Yes | Yes | Yes |
| 7 | Akcali 2007 | Yes | Yes | Yes | Yes | Yes | Not for all outcomes | Not for all outcomes | Yes | Yes |
| 8 | Akdeniz 1998 | Yes | Yes | No | Yes | Yes | Not for all outcomes | Not for all outcomes | Yes | Yes |
| 9 | Akinci 2006 | Yes | Yes | No | No | Yes | Yes | Yes | Yes | Yes |
| 10 | Akkoc 2023 | No | Yes | No | Yes | Yes | Yes | Yes | Yes | Yes |
| 11 | Al Hashan 2017 | No | Yes | No | Yes | Yes | Not for all outcomes | Not for all outcomes | Yes | Yes |
| 12 | Al Nokhatha 2021 | No | Yes | No | Yes | Yes | Not for all outcomes | Not for all outcomes | Yes | Yes |
| 13 | Al Sawafi 2023 | No | Yes | No | Yes | Yes | Not for all outcomes | Not for all outcomes | Yes | Yes |
| 14 | Al Shamahy 2001 | Yes | Yes | No | No | Yes | Not for all outcomes | Not for all outcomes | Yes | Yes |
| 15 | Al-Dubooni 1986 | No | Yes | No | No | Yes | Not for all outcomes | Not for all outcomes | Yes | Yes |
| 16 | Al-Eissa 1993 | No | Unclear | No | Yes | Yes | Not for all outcomes | Not for all outcomes | Yes | Yes |
| 17 | Al-Eissa1990 | No | Yes | No | Yes | Yes | Not for all outcomes | Not for all outcomes | Yes | Yes |
| 18 | Al-Freihi 1986 | Yes | Yes | No | Yes | Yes | Not for all outcomes | Not for all outcomes | Yes | Yes |
| 19 | Al-Koubaisy 2011 | Yes | Yes | Yes | Yes | Yes | Not for all outcomes | Not for all outcomes | Yes | Yes |
| 20 | AlRashidi 2022 | No | No | No | Yes | No | Not for all outcomes | Not for all outcomes | Yes | Unclear |
| 21 | Artuk 2019 | Yes | Unclear | Yes | Yes | Yes | Not for all outcomes | Not for all outcomes | Yes | Yes |
| 22 | Aygen 2002 | No | Unclear | Yes | Yes | Yes | Not for all outcomes | Not for all outcomes | Yes | Yes |
| 23 | Aypak 2012 | Yes | Yes | No | Yes | Yes | Not for all outcomes | Not for all outcomes | Yes | Yes |
| 24 | Magableh 2007 | Yes | Yes | Yes | No | Yes | Not for all outcomes | Not for all outcomes | Yes | Yes |
| 25 | Baykan 2019 | Yes | Yes | No | Yes | Yes | Yes | Yes | Yes | Yes |
| 26 | Bayram 1997 | Yes | Yes | Yes | No | Yes | Yes | Yes | Yes | Yes |
| 27 | Beheshti 2001 | Yes | Yes | No | Yes | No | Not for all outcomes | Not for all outcomes | Yes | Unclear |
| 28 | Behzadnia 2018 | No | Yes | No | Yes | Yes | Not for all outcomes | Not for all outcomes | Yes | Yes |
| 29 | Benbir 2013 | Yes | Yes | No | Yes | Yes | Yes | Yes | Yes | Yes |
| 30 | Benjamin 1992 | No | Yes | Yes | Yes | Yes | Not for all outcomes | Not for all outcomes | Yes | Yes |
| 31 | Besharat 2010 | No | Unclear | No | No | Yes | Not for all outcomes | Not for all outcomes | Yes | Yes |
| 32 | Bodur 2003 | No | Yes | No | Yes | Yes | Yes | Yes | Yes | Yes |
| 33 | Bozdemir 2017 | No | Yes | No | Yes | No | Not for all outcomes | Not for all outcomes | Yes | Unclear |
| 34 | Bozgeyik 2014 | Yes | Yes | Yes | Yes | Yes | Yes | Yes | Yes | Yes |
| 35 | Bozgeyik 2008 | Yes | Yes | No | Yes | Yes | Yes | Yes | Yes | Yes |
| 36 | Bukharie 2009 | No | Yes | No | Yes | Yes | Not for all outcomes | Not for all outcomes | Yes | Yes |
| 37 | Bulut 2011 | No | Yes | Yes | Yes | Yes | Yes | Yes | Yes | Yes |
| 38 | Caksen 2002 | No | Unclear | No | Yes | Yes | Not for all outcomes | Not for all outcomes | Yes | Yes |
| 39 | Celen 2010 | Yes | Yes | No | Yes | Yes | Not for all outcomes | Not for all outcomes | Yes | Yes |
| 40 | Celik 2023 | No | Yes | Yes | No | Yes | Yes | Yes | Yes | Yes |
| 41 | Çelik 2023 | Yes | Yes | No | Yes | Yes | Yes | Yes | Yes | Yes |
| 42 | Çiftdogan 2020 | No | Unclear | Yes | Yes | Yes | Not for all outcomes | Not for all outcomes | Yes | Yes |
| 43 | Çirakli 2015 | No | Unclear | No | Yes | Yes | Not for all outcomes | Not for all outcomes | Yes | Yes |
| 44 | Conkar 2018 | No | Unclear | No | Yes | Yes | Not for all outcomes | Not for all outcomes | Yes | Yes |
| 45 | Coskun 2004 | Yes | Unclear | No | No | Yes | Not for all outcomes | Not for all outcomes | Yes | Yes |
| 46 | Coskun 2009 | No | Yes | Yes | Yes | Yes | Yes | Yes | Yes | Yes |
| 47 | Dabaja-Younis 2023 | Yes | Yes | Yes | Yes | Yes | Not for all outcomes | Not for all outcomes | Yes | Yes |
| 48 | Dal 2013 | Yes | Yes | Yes | Yes | Yes | Not for all outcomes | Not for all outcomes | Yes | Yes |
| 49 | Demir 2014 | No | Unclear | No | Yes | Yes | Yes | Yes | Yes | Yes |
| 50 | Demirtuerk 2008 | Yes | Unclear | Yes | No | Yes | Not for all outcomes | Not for all outcomes | Yes | Yes |
| 51 | Ebrahimpour 2017 | Yes | Yes | Yes | No | Yes | Yes | Yes | Yes | Yes |
| 52 | Eini 2012 | Yes | Yes | No | Yes | Yes | Not for all outcomes | Not for all outcomes | Yes | Yes |
| 53 | El Bayoumy 2014 | Yes | Yes | Yes | Yes | Yes | Not for all outcomes | Not for all outcomes | Yes | Yes |
| 54 | Çiğdem 2019 | No | Yes | Yes | No | Yes | Not for all outcomes | Not for all outcomes | Yes | Yes |
| 55 | El-Amin 2001 | No | Yes | Yes | Yes | No | Not for all outcomes | Not for all outcomes | Yes | Yes |
| 56 | El-Koumi 2013 | No | Yes | No | Yes | Yes | Not for all outcomes | Not for all outcomes | Yes | Yes |
| 57 | Elhassanien 2014 | No | Unclear | No | Yes | Unclear | Not for all outcomes | Not for all outcomes | Yes | Yes |
| 58 | Elshamy 2008 | Yes | Unclear | No | Yes | Yes | Yes | Yes | Yes | Unclear |
| 59 | Erat 2023 | No | Unclear | Yes | Yes | Unclear | Not for all outcomes | Not for all outcomes | Yes | Unclear |
| 60 | Eroglu 2020 | No | Unclear | No | Yes | Unclear | Not for all outcomes | Not for all outcomes | Yes | Unclear |
| 61 | Ertek 2006 | Unclear | Unclear | Yes | No | Unclear | Not for all outcomes | Not for all outcomes | Yes | Unclear |
| 62 | Esmailpour 2010 | Yes | Yes | Yes | No | Yes | Yes | Yes | Yes | Yes |
| 63 | Fallah 2005 | Yes | Yes | Yes | Yes | Unclear | Not for all outcomes | Not for all outcomes | Yes | Yes |
| 64 | Fruchtman 2020 | No | Yes | No | Yes | Yes | Not for all outcomes | Not for all outcomes | Yes | Yes |
| 65 | Geyik 2002 | Yes | Unclear | Yes | Yes | Unclear | Yes | Yes | Yes | Unclear |
| 66 | Golsha 2011 | Yes | Yes | No | Yes | Yes | Not for all outcomes | Not for all outcomes | Yes | Yes |
| 67 | Gonen 2014 | No | Unclear | Yes | Yes | Unclear | Not for all outcomes | Not for all outcomes | Yes | Unclear |
| 68 | Gottesman 1996 | No | Yes | No | Yes | No | Not for all outcomes | Not for all outcomes | Yes | Yes |
| 69 | Gozdas 2020 | Unclear | Yes | Yes | No | Yes | Yes | Yes | Yes | Yes |
| 70 | Gul 2014 | Unclear | Unclear | Yes | Yes | Unclear | Not for all outcomes | Not for all outcomes | Yes | Unclear |
| 71 | Guler 2014 | Yes | Yes | Yes | Yes | Yes | Not for all outcomes | Not for all outcomes | Yes | Yes |
| 72 | Güngör 2002 | Yes | Yes | Yes | Yes | Yes | Not for all outcomes | Not for all outcomes | Yes | Yes |
| 73 | Gür 2003 | Yes | Yes | Yes | Yes | Yes | Not for all outcomes | Not for all outcomes | Yes | Yes |
| 74 | Hadadi 2009 | Yes | Yes | Yes | Yes | Yes | Not for all outcomes | Not for all outcomes | Yes | Yes |
| 75 | Hamid Hashemi 2016 | Unclear | Unclear | No | No | Unclear | Not for all outcomes | Not for all outcomes | Yes | Unclear |
| 76 | Hasanjani Roushan 2016 a | Yes | Yes | Yes | Yes | Yes | Not for all outcomes | Not for all outcomes | Yes | Yes |
| 77 | Hasanjani Roushan 2004 | No | Yes | Yes | Yes | Yes | Not for all outcomes | Not for all outcomes | Yes | Yes |
| 78 | Hasanjani Roushan 2016 b | No | Yes | Yes | Yes | Yes | Not for all outcomes | Not for all outcomes | Yes | Yes |
| 79 | Hashemi 2007 | No | Yes | Yes | Yes | Yes | No | No | Yes | Yes |
| 80 | Hassan 2021 | No | Yes | No | Yes | Yes | Not for all outcomes | Not for all outcomes | Yes | Yes |
| 81 | Hatipoglu 2005 | Yes | Unclear | No | No | Yes | Yes | Yes | Yes | Yes |
| 82 | Heidari 2011 | Unclear | Unclear | No | Yes | Unclear | No | No | Yes | Unclear |
| 83 | Hizel 2007 | Yes | Yes | Yes | Yes | Yes | Not for all outcomes | Not for all outcomes | Yes | Yes |
| 84 | Inan 2019 | Yes | Yes | Yes | Yes | Yes | Yes | Yes | Yes | Yes |
| 85 | Issa 1999 | No | Unclear | No | No | Unclear | Not for all outcomes | Not for all outcomes | Yes | Unclear |
| 86 | Kaptan 2013 | No | No | No | Yes | Unclear | Yes | Yes | Yes | Unclear |
| 87 | Karaoglan 2008 | Yes | Yes | Yes | No | Yes | Yes | Yes | Yes | Yes |
| 88 | Karli 2015 | No | Unclear | No | No | Unclear | Yes | Yes | Yes | Unclear |
| 89 | Kayaaslan 2016 | No | Yes | Yes | Yes | Yes | Not for all outcomes | Not for all outcomes | Yes | Yes |
| 90 | Kazak 2016 | Yes | Unclear | Yes | Yes | Unclear | Not for all outcomes | Not for all outcomes | Yes | Unclear |
| 91 | Keyvanfar 2021 | Yes | Yes | Yes | Yes | Yes | Not for all outcomes | Not for all outcomes | Yes | Yes |
| 92 | Khateeb 1990 | Yes | Yes | Yes | Yes | No | No | No | Yes | Yes |
| 93 | Kokoglu 2006 | No | Yes | Yes | Yes | Yes | Not for all outcomes | Not for all outcomes | Yes | Yes |
| 94 | Kursun 2013 | No | Unclear | Yes | Yes | Unclear | Yes | Yes | Yes | Unclear |
| 95 | Kurtaran 2012 | No | Unclear | Yes | Yes | Unclear | Not for all outcomes | Not for all outcomes | Yes | No |
| 96 | Kutlu 2018 | Yes | Yes | Yes | Yes | Yes | Not for all outcomes | Not for all outcomes | Yes | Yes |
| 97 | Lazutkin 2018 | Yes | Yes | Yes | Yes | Unclear | Yes | Yes | Yes | Unclear |
| 98 | Madkour 1988 | Yes | Yes | Yes | No | Yes | Yes | Yes | Yes | Yes |
| 99 | Majzoobi 2023 | Yes | Unclear | No | Yes | Unclear | Yes | Yes | Yes | Unclear |
| 100 | Malik 1997 | No | Unclear | No | Yes | Unclear | Not for all outcomes | Not for all outcomes | Yes | Unclear |
| 101 | McLean 1992 | Yes | Unclear | Yes | No | Unclear | Yes | Yes | Yes | Unclear |
| 102 | Megged 2016 | Yes | Yes | Yes | Yes | Yes | Not for all outcomes | Not for all outcomes | Yes | Yes |
| 103 | Ulug 2011 | No | Unclear | Yes | Yes | Unclear | Not for all outcomes | Not for all outcomes | Yes | Unclear |
| 104 | Mermut 2011 | Yes | Yes | Yes | No | Yes | Not for all outcomes | Not for all outcomes | Yes | Yes |
| 105 | Mohamed 1986 | Yes | Unclear | Yes | No | Unclear | Not for all outcomes | Not for all outcomes | Yes | Unclear |
| 106 | Mohammad Saeed 2012 | No | No | Yes | Yes | No | Not for all outcomes | Not for all outcomes | Yes | Unclear |
| 107 | Mousa 1988 | Yes | Yes | Yes | Yes | Yes | Not for all outcomes | Not for all outcomes | Yes | Yes |
| 108 | Mugahi 2014 | Yes | Yes | Yes | Yes | Yes | Not for all outcomes | Not for all outcomes | Yes | Yes |
| 109 | Nabavi 2019 | Yes | Yes | Yes | Yes | Yes | Not for all outcomes | Not for all outcomes | Yes | Yes |
| 110 | Najari 2018 | Yes | Yes | No | Yes | Yes | Not for all outcomes | Not for all outcomes | Yes | Yes |
| 111 | Namiduru 2003 | No | Yes | Yes | Yes | Yes | Not for all outcomes | Not for all outcomes | Yes | Yes |
| 112 | Norton 1984 | Yes | Yes | No | Yes | Yes | Not for all outcomes | Not for all outcomes | Yes | Yes |
| 113 | Okur 2012 | No | Yes | No | Yes | Yes | Not for all outcomes | Not for all outcomes | Yes | Yes |
| 114 | Özdem 2022 | No | Unclear | No | Yes | Unclear | Not for all outcomes | Not for all outcomes | Yes | Unclear |
| 115 | Özen 2022 | No | Unclear | Yes | Yes | No | Yes | Yes | Yes | No |
| 116 | Parlak 2015 | No | Unclear | Yes | Yes | Unclear | Not for all outcomes | Not for all outcomes | Yes | Unclear |
| 117 | Rajapakse 1987 | Yes | Yes | No | No | Yes | Yes | Yes | Yes | Yes |
| 118 | Rashad 2019 | No | Yes | No | No | Yes | Not for all outcomes | Not for all outcomes | Yes | Yes |
| 119 | Roushan 2005 | No | Yes | Yes | Yes | Yes | Yes | Yes | Yes | Yes |
| 120 | Roushan 2011 | Yes | Yes | No | Yes | Yes | Not for all outcomes | Not for all outcomes | Yes | Yes |
| 121 | Salman 2022 | No | Yes | Yes | Yes | No | Not for all outcomes | Not for all outcomes | Yes | Yes |
| 122 | Sanivar 2017 | Yes | Unclear | No | Yes | Unclear | Yes | Yes | Yes | Unclear |
| 123 | Savas 2007 | Yes | Yes | Yes | Yes | Yes | Not for all outcomes | Not for all outcomes | Yes | Yes |
| 124 | Savion 2023 | No | Yes | No | Yes | No | Not for all outcomes | Not for all outcomes | Yes | Yes |
| 125 | Sayad 2019 | Yes | Unclear | Yes | Yes | Unclear | Not for all outcomes | Not for all outcomes | Yes | Unclear |
| 126 | Shahnaz 2007 | No | Yes | No | Yes | Yes | Not for all outcomes | Not for all outcomes | Yes | Yes |
| 127 | Shalev 1994 | No | Yes | No | No | Yes | Yes | Yes | Yes | Yes |
| 128 | Zia Sheikholeslami 2007 | Yes | Yes | No | No | Yes | Not for all outcomes | Not for all outcomes | Yes | Yes |
| 129 | Simsek 2011 | Yes | Unclear | No | No | Unclear | Yes | Yes | Yes | Unclear |
| 130 | Somily 2017 | Yes | Yes | No | Yes | Yes | Not for all outcomes | Not for all outcomes | Yes | Yes |
| 131 | Sungur 2009 | Yes | Unclear | No | No | Unclear | Yes | Yes | Yes | Unclear |
| 132 | Tanir 2009 | No | Unclear | Yes | Yes | Unclear | Not for all outcomes | Not for all outcomes | Yes | Unclear |
| 133 | Tasbakan 2003 | Yes | Unclear | No | Yes | Unclear | Not for all outcomes | Not for all outcomes | Yes | Unclear |
| 134 | Tasova 1999 | Yes | Yes | No | Yes | Yes | Not for all outcomes | Not for all outcomes | Yes | Yes |
| 135 | Teke 2015 | No | Yes | No | No | Yes | Yes | No | Yes | Yes |
| 136 | Tekin 2012 | Yes | Unclear | Yes | Yes | Unclear | Not for all outcomes | Not for all outcomes | Yes | Unclear |
| 137 | Tohmé 2004 | Yes | Unclear | No | Yes | Unclear | Not for all outcomes | Not for all outcomes | Yes | Unclear |
| 138 | Turan 2011 | No | Unclear | No | Yes | Unclear | Not for all outcomes | Not for all outcomes | Yes | Unclear |
| 139 | Ulu-Kilic 2013 | No | Unclear | No | Yes | Unclear | Not for all outcomes | Not for all outcomes | Yes | Unclear |
| 140 | Varikkodan 2024 | No | Yes | Yes | Yes | No | Not for all outcomes | Not for all outcomes | Yes | Yes |
| 141 | Yetkin 2005 | No | Unclear | Yes | Yes | Unclear | Not for all outcomes | Not for all outcomes | Yes | Unclear |
| 142 | Yilmaz 2004 | Unclear | Unclear | Yes | No | No | Yes | Yes | Yes | Unclear |
| 143 | Zaks 1995 | Yes | Unclear | No | Yes | Unclear | Not for all outcomes | Not for all outcomes | Yes | Unclear |
| 144 | Zamani 2011 | No | Yes | No | Yes | Yes | Not for all outcomes | Not for all outcomes | Yes | Yes |
| 145 | Abbasi 2012 | No | Yes | Yes | No | Yes | Yes | Yes | Yes | Yes |
| 146 | Alsubaie 2005 | Yes | No | Yes | Yes | Unclear | Not for all outcomes | Not for all outcomes | Yes | Unclear |
| 147 | Kayaaslan 2013 | Yes | Unclear | No | Yes | Unclear | Not for all outcomes | Not for all outcomes | Yes | Unclear |
| 148 | Kiel 1987 | Yes | Yes | Yes | Yes | Yes | Not for all outcomes | Not for all outcomes | Yes | Yes |
| 149 | Akbayram 2011 | No | Yes | Yes | No | Yes | Yes | Yes | Yes | Yes |
| 150 | Kisacik 2011 | Yes | Unclear | No | No | Unclear | Not for all outcomes | Not for all outcomes | Yes | Unclear |
| 151 | Sagi 2017 | No | Yes | No | Yes | No | Not for all outcomes | Not for all outcomes | Yes | Unclear |
| 152 | Mousa 1986 | Yes | Yes | Yes | No | Yes | Not for all outcomes | Not for all outcomes | Yes | Yes |
| 153 | Kutlu 2009 | Yes | Unclear | No | No | Unclear | Yes | Yes | Yes | Unclear |
| 154 | Hasanjani Roushan 2009 | Yes | Yes | Yes | No | Yes | Yes | Yes | Yes | Yes |
| 155 | Samra 1983 | Yes | Yes | No | Yes | Yes | Not for all outcomes | Not for all outcomes | Yes | Yes |
| 156 | Feiz 1978 | No | Yes | No | Yes | Yes | Not for all outcomes | Not for all outcomes | Yes | Yes |
| 157 | Turunç 2008 | No | Yes | No | Yes | No | Yes | Yes | Yes | Yes |
| 158 | Naz 2009 | Yes | Unclear | Yes | Yes | Unclear | Not for all outcomes | Not for all outcomes | Yes | Unclear |
| 159 | Opawoye 1994 | No | Yes | No | Yes | Yes | Not for all outcomes | Not for all outcomes | Yes | Yes |
| 160 | Al Shaalan 2002 | No | Yes | Yes | Yes | Yes | Not for all outcomes | Not for all outcomes | Yes | Yes |
| 161 | Memish 2001 | No | Yes | No | No | Yes | Not for all outcomes | Not for all outcomes | Yes | Yes |
| 162 | Nabi 1992 | No | Yes | No | Yes | Yes | Not for all outcomes | Not for all outcomes | Yes | Yes |
| 163 | Gulsun 2011 | Yes | Unclear | No | Yes | Unclear | Yes | Yes | Yes | Unclear |
| 164 | Kurdoglu 2010 | Yes | Yes | No | Yes | No | Yes | Yes | Yes | Unclear |
| 165 | Khan 2001 | Yes | Yes | No | Yes | Unclear | Yes | Yes | Yes | Unclear |
| 166 | Rahil 2014 | No | Yes | No | Yes | Yes | Not for all outcomes | Not for all outcomes | Yes | Yes |
| 167 | Qasim 2020 | No | Yes | Yes | Yes | Yes | Not for all outcomes | Not for all outcomes | Yes | Yes |
| 168 | Suvak 2016 | No | Unclear | Yes | Yes | No | Yes | Yes | Yes | Unclear |
| 169 | Türker 2014 | No | Unclear | Yes | Yes | Unclear | Not for all outcomes | Not for all outcomes | Yes | Unclear |
| 170 | Patel 1988 | No | Unclear | Yes | No | Unclear | Yes | Yes | Yes | Unclear |
| 171 | Ertekin 2004 | No | Unclear | No | Yes | Unclear | Not for all outcomes | Not for all outcomes | Yes | Unclear |
| 172 | Torkaman 2017 | Yes | Unclear | No | Yes | Unclear | Not for all outcomes | Not for all outcomes | Yes | Unclear |
| 173 | Naz 2016 | Yes | Unclear | No | Yes | Unclear | Not for all outcomes | Not for all outcomes | Yes | Unclear |
| 174 | Uluğ 2011 | No | Unclear | No | Yes | Unclear | Not for all outcomes | Not for all outcomes | Yes | Unclear |
| 175 | Yoldas 2015 | No | Unclear | No | Yes | Unclear | Not for all outcomes | Not for all outcomes | Yes | Unclear |
| 176 | Buzgan 2010 | Yes | Unclear | Yes | Yes | Unclear | Not for all outcomes | Not for all outcomes | Yes | Unclear |
| 177 | Edathodu 2021 | No | Yes | Yes | Yes | Yes | Not for all outcomes | Not for all outcomes | Yes | Yes |
| 178 | Köse 2014 | Yes | Unclear | No | Yes | Unclear | Not for all outcomes | Not for all outcomes | Yes | Unclear |
| 179 | Fanni 2013 | No | Yes | No | Yes | Yes | Not for all outcomes | Not for all outcomes | Yes | Yes |
| 180 | Kaman 2022 | No | Unclear | No | Yes | Unclear | Not for all outcomes | Not for all outcomes | Yes | Unclear |
| 181 | Keramat 2009 | No | No | Yes | Yes | No | Not for all outcomes | Not for all outcomes | Yes | Unclear |
| 182 | Keramat 2018 | No | Unclear | Yes | Yes | Unclear | Not for all outcomes | Not for all outcomes | Yes | Unclear |
| 183 | Metin 2001 | Yes | Unclear | No | No | Unclear | Not for all outcomes | Not for all outcomes | Yes | Unclear |
| 184 | Kaya 2018 | No | Yes | Yes | Yes | Yes | Not for all outcomes | Not for all outcomes | Yes | Yes |
| 185 | Shehabi 1990 | Yes | Yes | Yes | Yes | Yes | Not for all outcomes | Not for all outcomes | Yes | Yes |
| 186 | Aktug-Demir 2014 | No | Yes | Yes | Yes | Yes | Not for all outcomes | Not for all outcomes | Yes | Yes |
| 187 | Najafi 2018 | Yes | Yes | Yes | Yes | Yes | Not for all outcomes | Not for all outcomes | Yes | Yes |
| 188 | Rahmanpour 2019 | Yes | Unclear | No | Yes | Unclear | Not for all outcomes | Not for all outcomes | Yes | Unclear |
| 189 | Majzoobi 2018 | No | Unclear | No | Yes | Yes | Not for all outcomes | Not for all outcomes | Yes | Yes |
| 190 | Saltoglu 2002 | Yes | Unclear | No | Yes | Unclear | Yes | Yes | Yes | Unclear |
| 191 | Pourbagher 2006 | Yes | Unclear | Yes | Yes | Unclear | Not for all outcomes | Not for all outcomes | Yes | Unclear |
| 192 | Pourakbari 2019 | No | Yes | No | Yes | Yes | Not for all outcomes | Not for all outcomes | Yes | Yes |
| 193 | Firouzeh 2019 | Yes | Yes | Yes | Yes | Yes | Not for all outcomes | Not for all outcomes | Yes | Yes |
| 194 | El-Kafas 1999 | No | Yes | No | Yes | Yes | Not for all outcomes | Not for all outcomes | Yes | Yes |
| 195 | Mohammadbeigi 2021 | Yes | Yes | Yes | Yes | Yes | Not for all outcomes | Not for all outcomes | Yes | Yes |
| 196 | Hatami 2019 | Yes | Yes | Yes | Yes | Yes | Not for all outcomes | Not for all outcomes | Yes | Yes |
| 197 | Hatami 2010 | Yes | Yes | Yes | Yes | Yes | Yes | Yes | Yes | Yes |
| 198 | Ayatollahi 2004 | Yes | Yes | Yes | Yes | Yes | Not for all outcomes | Not for all outcomes | Yes | Yes |
| 199 | Sabbaghian 1974 | Yes | Unclear | Yes | Yes | Unclear | Not for all outcomes | Not for all outcomes | Yes | Unclear |
| 200 | Vafaei 2019 | No | Yes | No | Yes | No | Not for all outcomes | Not for all outcomes | Yes | Yes |
| 201 | Biglarzadeh 2020 | Yes | Yes | Yes | No | Yes | Not for all outcomes | Not for all outcomes | Yes | Yes |
| 202 | Kuruoglu 2023 | Yes | Yes | Yes | Yes | No | Not for all outcomes | Not for all outcomes | Yes | Yes |
| 203 | Demir 2021 | No | Unclear | Yes | Yes | Unclear | Not for all outcomes | Not for all outcomes | Yes | Unclear |
| 204 | Zilberman 2022 | Yes | Yes | No | Yes | Yes | Not for all outcomes | Not for all outcomes | Yes | Yes |
| 205 | Bayazit 2002 | Yes | Unclear | No | No | Unclear | Not for all outcomes | Not for all outcomes | Yes | Unclear |
| 206 | Citak 2010 | No | Yes | Yes | No | Yes | Yes | Yes | Yes | Yes |
| 207 | Aypak 2015 | No | Yes | No | Yes | Yes | Yes | Yes | Yes | Yes |
| 208 | Gholami 2012 | Yes | Unclear | No | Yes | Unclear | Not for all outcomes | Not for all outcomes | Yes | Unclear |
| 209 | Shimol 2012 | Yes | Yes | No | Yes | Yes | Not for all outcomes | Not for all outcomes | Yes | Yes |
| 210 | Mohamed 2018 | Unclear | Unclear | No | Yes | Unclear | Not for all outcomes | Not for all outcomes | Yes | Unclear |
| 211 | Ibrahim 2021 | Yes | Yes | Yes | Yes | Yes | Not for all outcomes | Not for all outcomes | Yes | Yes |
| 212 | Hashemi 2018 | Yes | Yes | No | Yes | Yes | Not for all outcomes | Not for all outcomes | Yes | Yes |
| 213 | Eskandari-Nasab 2014 | Yes | Unclear | Yes | No | Unclear | Not for all outcomes | Not for all outcomes | Yes | Unclear |
| 214 | Kaygusuz 2005 | Yes | Unclear | No | Yes | Unclear | Not for all outcomes | Not for all outcomes | Yes | Unclear |
| 215 | Sayin-Kutlu 2012 | No | Unclear | No | Yes | No | Not for all outcomes | Not for all outcomes | Yes | Yes |
| 216 | Sari 2008 | No | Unclear | Yes | No | Unclear | Not for all outcomes | Not for all outcomes | Yes | Unclear |
| 217 | Demiraslan 2009 | No | Yes | Yes | No | Yes | Yes | Yes | Yes | Yes |
| 218 | Guven 2013 | No | Unclear | No | No | Unclear | Not for all outcomes | Not for all outcomes | Yes | Unclear |
| 219 | Ranjbar 2009 | Yes | Unclear | Yes | No | Unclear | Not for all outcomes | Not for all outcomes | Yes | Unclear |
| 220 | Karabay 2004 | No | Unclear | No | Yes | Unclear | Not for all outcomes | Not for all outcomes | Yes | Unclear |
| 221 | Karakurt 2023 | Yes | Unclear | No | Yes | Unclear | Not for all outcomes | Not for all outcomes | Yes | Unclear |
| 222 | Ertem 2004 | Yes | Unclear | No | Yes | Unclear | Not for all outcomes | Not for all outcomes | Yes | Unclear |
| 223 | Paul 2017 | Yes | Yes | No | Yes | Yes | Not for all outcomes | Not for all outcomes | Yes | Yes |
| 224 | Karakukcu 2004 | No | Unclear | No | No | Unclear | Yes | Yes | Yes | Unclear |
| 225 | Olt 2015 | Yes | Unclear | No | No | Unclear | Not for all outcomes | Not for all outcomes | Yes | Unclear |
| 226 | Sen 2019 | No | Unclear | Yes | Yes | Unclear | Not for all outcomes | Not for all outcomes | Yes | Unclear |
| 227 | Kara 2019 | No | Unclear | No | Yes | Unclear | Not for all outcomes | Not for all outcomes | Yes | Unclear |
| 228 | Aghaali 2015 | No | Yes | No | Yes | Yes | Not for all outcomes | Not for all outcomes | Yes | Yes |
| 229 | Ahmadinejad 2016 | Yes | Yes | No | Yes | Yes | Not for all outcomes | Not for all outcomes | Yes | Yes |
| 230 | Lubani 1989 | Yes | Yes | Yes | No | Yes | Yes | Yes | Yes | Yes |
| 231 | Salari 2003 | Yes | Yes | Yes | Yes | Yes | Not for all outcomes | Not for all outcomes | Yes | Yes |
| 232 | Cuzdan 2022 | No | Yes | Yes | Yes | Yes | Not for all outcomes | Not for all outcomes | Yes | Yes |
| 233 | Alim 2015 | Yes | No | No | Yes | Yes | Not for all outcomes | Not for all outcomes | Yes | Yes |
| 234 | Kahbazi 2012 | No | Unclear | No | No | No | Not for all outcomes | Not for all outcomes | Yes | No |
| 235 | Lifeso 1985 | Yes | Yes | No | No | Yes | Not for all outcomes | Not for all outcomes | Yes | Yes |
| 236 | Kadanali 2005 | Yes | Unclear | No | Yes | Unclear | Not for all outcomes | Not for all outcomes | Yes | Unclear |
| 237 | Rezazadeh 2006 | Yes | Unclear | Yes | Yes | Unclear | Not for all outcomes | Not for all outcomes | Yes | Unclear |
| 238 | Çiftdoğan 2017 | Yes | Unclear | Yes | Yes | Unclear | Not for all outcomes | Not for all outcomes | Yes | Unclear |
| 239 | Najafi 2011 | Yes | Yes | Yes | No | Yes | Yes | Yes | Yes | Yes |
| 240 | Ergun 2008 | Yes | Unclear | No | Yes | Unclear | Yes | Yes | Yes | Unclear |

B) Per-outcome risk of bias assessment:

*Table S4-2. Musculoskeletal involvement.*

|  | Study ID | Overall risk of bias |
| --- | --- | --- |
| 1 | Al Nokhatha 2021 | High |
| 2 | Artuk 2019 | High |
| 3 | Bozgeyik 2014 | Low |
| 4 | Ertek 2006 | High |
| 5 | Geyik 2002 | High |
| 6 | Gul 2014 | High |
| 7 | Heidari 2011 | High |
| 8 | Kursun 2013 | High |
| 9 | Madkour 1988 | High |
| 10 | Turunç 2008 | High |
| 11 | Ahmadinejad 2016 | High |

*Table S4-3. Skeletal involvement.*

|  | Study ID | Overall risk of bias |
| --- | --- | --- |
| 1 | Ebrahimpour 2017 | High |
| 2 | Ertek 2006 | High |
| 3 | Gul 2014 | High |
| 4 | Ulug 2011 | High |

*Table S4-4. Osteoarticular involvement.*

|  | Study ID | Overall risk of bias |
| --- | --- | --- |
| 1 | Akkoc 2023 | High |
| 2 | Al-Eissa1990 | High |
| 3 | Aygen 2002 | High |
| 4 | Benjamin 1992 | High |
| 5 | Bukharie 2009 | High |
| 6 | Bulut 2011 | High |
| 7 | Çiftdogan 2020 | High |
| 8 | Dal 2013 | High |
| 9 | Demirtuerk 2008 | High |
| 10 | Eroglu 2020 | High |
| 11 | Ertek 2006 | High |
| 12 | Gonen 2014 | High |
| 13 | Gottesman 1996 | High |
| 14 | Guler 2014 | Low |
| 15 | Gür 2003 | High |
| 16 | Hadadi 2009 | High |
| 17 | Hamid Hashemi 2016 | High |
| 18 | Hashemi 2007 | High |
| 19 | Kayaaslan 2016 | High |
| 20 | Kazak 2016 | High |
| 21 | Keyvanfar 2021 | High |
| 22 | Kokoglu 2006 | High |
| 23 | Megged 2016 | High |
| 24 | Ulug 2011 | High |
| 25 | Mermut 2011 | High |
| 26 | Mousa 1988 | Low |
| 27 | Namiduru 2003 | High |
| 28 | Savas 2007 | High |
| 29 | Sayad 2019 | High |
| 30 | Tasova 1999 | High |
| 31 | Tohmé 2004 | High |
| 32 | Turan 2011 | High |
| 33 | Varikkodan 2024 | High |
| 34 | Yetkin 2005 | High |
| 35 | Kayaaslan 2013 | High |
| 36 | Naz 2009 | High |
| 37 | Türker 2014 | High |
| 38 | Uluğ 2011 | High |
| 39 | Buzgan 2010 | High |
| 40 | Edathodu 2021 | High |
| 41 | Pourbagher 2006 | High |
| 42 | Kuruoglu 2023 | High |
| 43 | Ertem 2004 | High |
| 44 | Paul 2017 | High |
| 45 | Sen 2019 | High |
| 46 | Kadanali 2005 | High |

*Table S4-5. Sacroiliitis.*

|  | Study ID | Overall risk of bias |
| --- | --- | --- |
| 1 | Akkoc 2023 | High |
| 2 | Al Nokhatha 2021 | High |
| 3 | Artuk 2019 | High |
| 4 | Aygen 2002 | High |
| 5 | Benjamin 1992 | High |
| 6 | Bozgeyik 2014 | Low |
| 7 | Çelik 2023 | High |
| 8 | Çiftdogan 2020 | High |
| 9 | Dabaja-Younis 2023 | Low |
| 10 | Demirtuerk 2008 | High |
| 11 | Ebrahimpour 2017 | High |
| 12 | Eroglu 2020 | High |
| 13 | Ertek 2006 | High |
| 14 | Geyik 2002 | High |
| 15 | Gonen 2014 | High |
| 16 | Gottesman 1996 | High |
| 17 | Gul 2014 | High |
| 18 | Guler 2014 | Low |
| 19 | Güngör 2002 | High |
| 20 | Gür 2003 | High |
| 21 | Hadadi 2009 | High |
| 22 | Hamid Hashemi 2016 | High |
| 23 | Hasanjani Roushan 2016 a | Low |
| 24 | Hasanjani Roushan 2004 | High |
| 25 | Hasanjani Roushan 2016 b | High |
| 26 | Hashemi 2007 | High |
| 27 | Hassan 2021 | High |
| 28 | Heidari 2011 | High |
| 29 | Hizel 2007 | High |
| 30 | Kayaaslan 2016 | High |
| 31 | Kazak 2016 | High |
| 32 | Keyvanfar 2021 | Low |
| 33 | Khateeb 1990 | High |
| 34 | Kokoglu 2006 | High |
| 35 | Kursun 2013 | High |
| 36 | Kurtaran 2012 | High |
| 37 | Kutlu 2018 | Low |
| 38 | Madkour 1988 | High |
| 39 | Ulug 2011 | High |
| 40 | Mermut 2011 | High |
| 41 | Mousa 1988 | Low |
| 42 | Najari 2018 | High |
| 43 | Namiduru 2003 | High |
| 44 | Özdem 2022 | High |
| 45 | Özen 2022 | High |
| 46 | Rajapakse 1987 | High |
| 47 | Roushan 2005 | High |
| 48 | Salman 2022 | High |
| 49 | Savas 2007 | High |
| 50 | Sayad 2019 | High |
| 51 | Tasova 1999 | High |
| 52 | Tekin 2012 | High |
| 53 | Turan 2011 | High |
| 54 | Ulu-Kilic 2013 | High |
| 55 | Kayaaslan 2013 | High |
| 56 | Kisacik 2011 | High |
| 57 | Naz 2009 | High |
| 58 | Al Shaalan 2002 | High |
| 59 | Türker 2014 | High |
| 60 | Torkaman 2017 | High |
| 61 | Uluğ 2011 | High |
| 62 | Yoldas 2015 | High |
| 63 | Buzgan 2010 | High |
| 64 | Köse 2014 | High |
| 65 | Keramat 2018 | High |
| 66 | Kaya 2018 | High |
| 67 | Aktug-Demir 2014 | High |
| 68 | Najafi 2018 | High |
| 69 | Saltoglu 2002 | High |
| 70 | Pourbagher 2006 | High |
| 71 | Ayatollahi 2004 | High |
| 72 | Demir 2021 | High |
| 73 | Gholami 2012 | High |
| 74 | Hashemi 2018 | High |
| 75 | Sayin-Kutlu 2012 | High |
| 76 | Karabay 2004 | High |
| 77 | Olt 2015 | High |
| 78 | Sen 2019 | High |
| 79 | Aghaali 2015 | High |
| 80 | Kadanali 2005 | High |
| 81 | Cuzdan 2022 | High |

*Table S4-6. Sacroiliitis and sclerosis.*

|  | Study ID | Overall risk of bias |
| --- | --- | --- |
| 1 | Geyik 2002 | High |
| 2 | Tasova 1999 | Low |

*Table S4-7. Sacroiliitis and ankylosis.*

|  | Study ID | Overall risk of bias |
| --- | --- | --- |
| 1 | Geyik 2002 | High |

*Table S4-8. Sacroiliitis and spondylodiscitis.*

|  | Study ID | Overall risk of bias |
| --- | --- | --- |
| 1 | Savas 2007 | High |
| 2 | Turan 2011 | High |

*Table S4-9. Sacroiliitis and spondylodiscitis and arthritis.*

|  | Study ID | Overall risk of bias |
| --- | --- | --- |
| 1 | Savas 2007 | High |

*Table S4-10. Spondylitis.*

|  | Study ID | Overall risk of bias |
| --- | --- | --- |
| 1 | Abbasi 2020 | High |
| 2 | Abdi-Liae 2007 | High |
| 3 | Akkoc 2023 | High |
| 4 | Al-Eissa1990 | High |
| 5 | Artuk 2019 | High |
| 6 | Aygen 2002 | High |
| 7 | Benjamin 1992 | High |
| 8 | Bodur 2003 | High |
| 9 | Çiftdogan 2020 | High |
| 10 | Ebrahimpour 2017 | High |
| 11 | Eini 2012 | High |
| 12 | Eroglu 2020 | High |
| 13 | Ertek 2006 | High |
| 14 | Geyik 2002 | High |
| 15 | Golsha 2011 | High |
| 16 | Gul 2014 | High |
| 17 | Güngör 2002 | Low |
| 18 | Gür 2003 | Low |
| 19 | Hadadi 2009 | High |
| 20 | Hasanjani Roushan 2016 a | Low |
| 21 | Hasanjani Roushan 2004 | High |
| 22 | Hasanjani Roushan 2016 b | High |
| 23 | Hashemi 2007 | High |
| 24 | Heidari 2011 | High |
| 25 | Kayaaslan 2016 | High |
| 26 | Keyvanfar 2021 | High |
| 27 | Kokoglu 2006 | High |
| 28 | Ulug 2011 | High |
| 29 | Mousa 1988 | Low |
| 30 | Namiduru 2003 | High |
| 31 | Norton 1984 | High |
| 32 | Roushan 2005 | High |
| 33 | Salman 2022 | High |
| 34 | Sayad 2019 | High |
| 35 | Tasova 1999 | Low |
| 36 | Tohmé 2004 | High |
| 37 | Yilmaz 2004 | High |
| 38 | Kayaaslan 2013 | High |
| 39 | Naz 2009 | High |
| 40 | Memish 2001 | High |
| 41 | Torkaman 2017 | High |
| 42 | Uluğ 2011 | High |
| 43 | Buzgan 2010 | High |
| 44 | Keramat 2018 | High |
| 45 | Aktug-Demir 2014 | High |
| 46 | Najafi 2018 | High |
| 47 | Majzoobi 2018 | High |
| 48 | Gholami 2012 | High |
| 49 | Hashemi 2018 | High |
| 50 | Eskandari-Nasab 2014 | High |
| 51 | Ertem 2004 | High |
| 52 | Cuzdan 2022 | High |
| 53 | Lifeso 1985 | High |
| 54 | Kadanali 2005 | High |

*Table S4-11. Spondylodiscitis.*

|  | Study ID | Overall risk of bias |
| --- | --- | --- |
| 1 | Akkoc 2023 | High |
| 2 | Bozgeyik 2014 | Low |
| 3 | Bozgeyik 2008 | High |
| 4 | Demir 2014 | High |
| 5 | Demirtuerk 2008 | High |
| 6 | El Bayoumy 2014 | Low |
| 7 | Geyik 2002 | High |
| 8 | Gonen 2014 | High |
| 9 | Guler 2014 | Low |
| 10 | Hassan 2021 | High |
| 11 | Kaptan 2013 | High |
| 12 | Kazak 2016 | High |
| 13 | Kursun 2013 | High |
| 14 | Kutlu 2018 | High |
| 15 | Mermut 2011 | High |
| 16 | Savas 2007 | High |
| 17 | Tasbakan 2003 | High |
| 18 | Turan 2011 | High |
| 19 | Ulu-Kilic 2013 | High |
| 20 | Türker 2014 | High |
| 21 | Köse 2014 | High |
| 22 | Keramat 2018 | High |
| 23 | Kaya 2018 | High |
| 24 | Pourbagher 2006 | High |
| 25 | Kuruoglu 2023 | High |
| 26 | Sayin-Kutlu 2012 | High |
| 27 | Sen 2019 | High |
| 28 | Cuzdan 2022 | High |

*Table S4-12. Spondylodiscitis and arthritis.*

|  | Study ID | Overall risk of bias |
| --- | --- | --- |
| 1 | Savas 2007 | High |
| 2 | Turan 2011 | High |

*Table S4-13. Spondylitis and epiphysitis.*

|  | Study ID | Overall risk of bias |
| --- | --- | --- |
| 1 | Tasova 1999 | Low |
| 2 | Firouzeh 2019 | High |
| 3 | Biglarzadeh 2020 | High |

*Table S4-14. Spondylitis or sacroillitis.*

|  | Study ID | Overall risk of bias |
| --- | --- | --- |
| 1 | Varikkodan 2024 | High |

*Table S4-15. Spondylitis or spondylodiscitis.*

|  | Study ID | Overall risk of bias |
| --- | --- | --- |
| 1 | Kurtaran 2012 | High |
|  | Namiduru 2003 | High |

*Table S4-16. Discitis.*

|  | Study ID | Overall risk of bias |
| --- | --- | --- |
| 1 | Bodur 2003 | High |
| 2 | Bozgeyik 2008 | High |
| 3 | Tanir 2009 | High |
| 4 | Naz 2009 | High |

*Table S4-17. Lumbar abscess.*

|  | Study ID | Overall risk of bias |
| --- | --- | --- |
| 1 | Sen 2019 | High |

*Table S4-18. Subchondral erosion.*

|  | Study ID | Overall risk of bias |
| --- | --- | --- |
| 1 | Geyik 2002 | High |

*Table S4-19. Iliac bone involvement.*

|  | Study ID | Overall risk of bias |
| --- | --- | --- |
| 1 | Çirakli 2015 | High |

*Table S4-20. Spinal brucellosis.*

|  | Study ID | Overall risk of bias |
| --- | --- | --- |
| 1 | Akkoc 2023 | High |
| 2 | Fallah 2005 | High |
| 3 | Madkour 1988 | High |
| 4 | Namiduru 2003 | High |
| 5 | Norton 1984 | High |
| 6 | Roushan 2005 | High |
| 7 | Ulu-Kilic 2013 | High |
| 8 | Varikkodan 2024 | High |
| 9 | Edathodu 2021 | High |
| 10 | Lifeso 1985 | High |

*Table S4-21. Extra-spinal skeleton lesions.*

|  | Study ID | Overall risk of bias |
| --- | --- | --- |
| 1 | Madkour 1988 | High |

*Table S4-22. Spinal cord involvement (vertebral osteomyelitis).*

|  | Study ID | Overall risk of bias |
| --- | --- | --- |
| 1 | Dabaja-Younis 2023 | Low |
| 2 | Mohammad Saeed 2012 | High |
| 3 | Rahil 2014 | High |

*Table S4-23. Arthritis or peripheral arthritis.*

|  | Study ID | Overall risk of bias |
| --- | --- | --- |
| 1 | Abbasi 2020 | High |
| 2 | Abdi-Liae 2007 | High |
| 3 | Afify 2013 | High |
| 4 | Akdeniz 1998 | High |
| 5 | Akkoc 2023 | High |
| 6 | Al Nokhatha 2021 | High |
| 7 | Al Sawafi 2023 | High |
| 8 | Al Shamahy 2001 | High |
| 9 | Al-Dubooni 1986 | High |
| 10 | Al-Eissa 1993 | High |
| 11 | Al-Eissa1990 | High |
| 12 | Al-Freihi 1986 | High |
| 13 | AlRashidi 2022 | High |
| 14 | Artuk 2019 | High |
| 15 | Aygen 2002 | High |
| 16 | Aypak 2012 | High |
| 17 | Beheshti 2001 | High |
| 18 | Behzadnia 2018 | High |
| 19 | Benjamin 1992 | High |
| 20 | Besharat 2010 | High |
| 21 | Bozdemir 2017 | High |
| 22 | Bozgeyik 2014 | Low |
| 23 | Caksen 2002 | High |
| 24 | Çiftdogan 2020 | High |
| 25 | Çirakli 2015 | High |
| 26 | Dabaja-Younis 2023 | Low |
| 27 | Ebrahimpour 2017 | High |
| 28 | Eini 2012 | High |
| 29 | El-Amin 2001 | High |
| 30 | Elhassanien 2014 | High |
| 31 | Erat 2023 | High |
| 32 | Eroglu 2020 | High |
| 33 | Ertek 2006 | High |
| 34 | Fruchtman 2020 | High |
| 35 | Geyik 2002 | High |
| 36 | Gonen 2014 | High |
| 37 | Gottesman 1996 | High |
| 38 | Gul 2014 | High |
| 39 | Guler 2014 | Low |
| 40 | Güngör 2002 | High |
| 41 | Gür 2003 | High |
| 42 | Hadadi 2009 | High |
| 43 | Hasanjani Roushan 2016 a | Low |
| 44 | Hasanjani Roushan 2004 | High |
| 45 | Hasanjani Roushan 2016 b | High |
| 46 | Hashemi 2007 | High |
| 47 | Hassan 2021 | High |
| 48 | Heidari 2011 | High |
| 49 | Hizel 2007 | High |
| 50 | Issa 1999 | High |
| 51 | Kayaaslan 2016 | High |
| 52 | Kazak 2016 | High |
| 53 | Khateeb 1990 | High |
| 54 | Kokoglu 2006 | High |
| 55 | Kursun 2013 | High |
| 56 | Kurtaran 2012 | High |
| 57 | Kutlu 2018 | Low |
| 58 | Malik 1997 | High |
| 59 | Ulug 2011 | High |
| 60 | Mermut 2011 | High |
| 61 | Mohammad Saeed 2012 | High |
| 62 | Mousa 1988 | Low |
| 63 | Mugahi 2014 | High |
| 64 | Najari 2018 | High |
| 65 | Namiduru 2003 | High |
| 66 | Norton 1984 | High |
| 67 | Okur 2012 | High |
| 68 | Özdem 2022 | High |
| 69 | Özen 2022 | High |
| 70 | Parlak 2015 | High |
| 71 | Roushan 2005 | High |
| 72 | Salman 2022 | High |
| 73 | Savas 2007 | High |
| 74 | Sayad 2019 | High |
| 75 | Shahnaz 2007 | High |
| 76 | Zia Sheikholeslami 2007 | High |
| 77 | Tanir 2009 | High |
| 78 | Tasova 1999 | Low |
| 79 | Turan 2011 | High |
| 80 | Ulu-Kilic 2013 | High |
| 81 | Varikkodan 2024 | High |
| 82 | Zaks 1995 | High |
| 83 | Zamani 2011 | High |
| 84 | Alsubaie 2005 | High |
| 85 | Kayaaslan 2013 | High |
| 86 | Kisacik 2011 | High |
| 87 | Samra 1983 | High |
| 88 | Feiz 1978 | High |
| 89 | Turunç 2008 | High |
| 90 | Naz 2009 | High |
| 91 | Al Shaalan 2002 | High |
| 92 | Türker 2014 | High |
| 93 | Ertekin 2004 | High |
| 94 | Torkaman 2017 | High |
| 95 | Uluğ 2011 | High |
| 96 | Yoldas 2015 | High |
| 97 | Buzgan 2010 | High |
| 98 | Edathodu 2021 | High |
| 99 | Fanni 2013 | High |
| 100 | Kaman 2022 | High |
| 101 | Keramat 2009 | High |
| 102 | Keramat 2018 | High |
| 103 | Kaya 2018 | High |
| 104 | Shehabi 1990 | High |
| 105 | Najafi 2018 | High |
| 106 | Majzoobi 2018 | High |
| 107 | Saltoglu 2002 | High |
| 108 | Pourakbari 2019 | High |
| 109 | Firouzeh 2019 | High |
| 110 | El-Kafas 1999 | High |
| 111 | Mohammadbeigi 2021 | High |
| 112 | Biglarzadeh 2020 | High |
| 113 | Demir 2021 | High |
| 114 | Eskandari-Nasab 2014 | High |
| 115 | Sen 2019 | High |
| 116 | Kara 2019 | High |
| 117 | Salari 2003 | High |
| 118 | Cuzdan 2022 | High |
| 119 | Kahbazi 2012 | High |
| 120 | Kadanali 2005 | High |
| 121 | Rezazadeh 2006 | High |
| 122 | Çiftdoğan 2017 | High |

*Table S4-24. Arthritis with effusion.*

|  | Study ID | Overall risk of bias |
| --- | --- | --- |
| 1 | Khateeb 1990 | High |

*Table S4-25. Peripheral joint involvement.*

|  | Study ID | Overall risk of bias |
| --- | --- | --- |
| 1 | Varikkodan 2024 | High |

*Table S4-26. Arthritis and sacroiliitis and spondylodiscitis and periostitis.*

|  | Study ID | Overall risk of bias |
| --- | --- | --- |
| 1 | Savas 2007 | High |

*Table S4-27. Arthritis and spondylodiscitis, and synovitis.*

|  | Study ID | Overall risk of bias |
| --- | --- | --- |
| 1 | Savas 2007 | High |

*Table S4-28. Sternoclavicular arthritis.*

|  | Study ID | Overall risk of bias |
| --- | --- | --- |
| 1 | Ebrahimpour 2017 | High |
| 2 | Geyik 2002 | High |
| 3 | Hasanjani Roushan 2004 | High |
| 4 | Madkour 1988 | High |
| 5 | Mousa 1988 | Low |
| 6 | Uluğ 2011 | High |

*Table S4-29. Arthropathy.*

|  | Study ID | Overall risk of bias |
| --- | --- | --- |
| 1 | El-Amin 2001 | High |
| 2 | Rashad 2019 | High |
| 3 | Lifeso 1985 | High |

*Table S4-30. Pauciarticular joint involvement.*

|  | Study ID | Overall risk of bias |
| --- | --- | --- |
| 1 | Çiftdogan 2020 | High |

*Table S4-31. Osteomyelitis.*

|  | Study ID | Overall risk of bias |
| --- | --- | --- |
| 1 | Al-Eissa1990 | High |
| 2 | AlRashidi 2022 | High |
| 3 | Çiftdogan 2020 | High |
| 4 | Çirakli 2015 | High |
| 5 | Dabaja-Younis 2023 | Low |
| 6 | Geyik 2002 | High |
| 7 | Gonen 2014 | High |
| 8 | Gottesman 1996 | High |
| 9 | Guler 2014 | Low |
| 10 | Gür 2003 | Low |
| 11 | Hasanjani Roushan 2016 a | Low |
| 12 | Keyvanfar 2021 | Low |
| 13 | Khateeb 1990 | High |
| 14 | Kokoglu 2006 | High |
| 15 | Mousa 1988 | Low |
| 16 | Salman 2022 | High |
| 17 | Savas 2007 | High |
| 18 | Savion 2023 | High |
| 19 | Kiel 1987 | High |
| 20 | Sagi 2017 | High |
| 21 | Naz 2009 | High |
| 22 | Yoldas 2015 | High |
| 23 | Najafi 2018 | High |
| 24 | Pourbagher 2006 | High |
| 25 | Lifeso 1985 | High |

*Table S4-32. Long-bone osteomyelitis.*

|  | Study ID | Overall risk of bias |
| --- | --- | --- |
| 1 | Madkour 1988 | High |

*Table S4-33. Joint effusion.*

|  | Study ID | Overall risk of bias |
| --- | --- | --- |
| 1 | Geyik 2002 | High |
| 2 | Khateeb 1990 | High |

*Table S4-34. Interapophysial involvement.*

|  | Study ID | Overall risk of bias |
| --- | --- | --- |
| 1 | Geyik 2002 | High |

*Table S4-35. Vertebral Osteomyelitis/discitis.*

|  | Study ID | Overall risk of bias |
| --- | --- | --- |
| 1 | Dabaja-Younis 2023 | Low |

*Table S4-36. Epidural abscess.*

|  | Study ID | Overall risk of bias |
| --- | --- | --- |
| 1 | Bodur 2003 | High |
| 2 | Demirtuerk 2008 | High |
| 3 | Turan 2011 | High |
| 4 | Biglarzadeh 2020 | High |

*Table S4-37. Vertebral abscess.*

|  | Study ID | Overall risk of bias |
| --- | --- | --- |
| 1 | Bozgeyik 2014 | Low |
| 2 | Türker 2014 | High |

*Table S4-38. Epidural abscess and Psoas muscle abscess.*

|  | Study ID | Overall risk of bias |
| --- | --- | --- |
| 1 | Turan 2011 | High |

*Table S4-39. Iliacus muscle abscess.*

|  | Study ID | Overall risk of bias |
| --- | --- | --- |
| 1 | Turan 2011 | High |

*Table S4-40. Paravertebral abscess.*

|  | Study ID | Overall risk of bias |
| --- | --- | --- |
| 1 | Bodur 2003 | High |
| 2 | Guler 2014 | Low |
| 3 | Kazak 2016 | High |
| 4 | Namiduru 2003 | High |
| 5 | Tasova 1999 | Low |
| 6 | Buzgan 2010 | High |
| 7 | Keramat 2018 | High |
| 8 | Kadanali 2005 | High |

*Table S4-41. Paravertebral or Psoas abscess.*

|  | Study ID | Overall risk of bias |
| --- | --- | --- |
| 1 | Hizel 2007 | High |

*Table S4-42. Paravertebral or epidural abscess.*

|  | Study ID | Overall risk of bias |
| --- | --- | --- |
| 1 | Geyik 2002 | High |
|  | Pourbagher 2006 | High |

*Table S4-43. Paravertebral soft tissue inflammation.*

|  | Study ID | Overall risk of bias |
| --- | --- | --- |
| 1 | Namiduru 2003 | High |

*Table S4-44. Bone abscess.*

|  | Study ID | Overall risk of bias |
| --- | --- | --- |
| 1 | El Bayoumy 2014 | High |

*Table S4-45. Vertebral osteophyte formation.*

|  | Study ID | Overall risk of bias |
| --- | --- | --- |
| 1 | Namiduru 2003 | High |

*Table S4-46. Joint de-arrangement.*

|  | Study ID | Overall risk of bias |
| --- | --- | --- |
| 1 | Bozgeyik 2014 | Low |

*Table S4-47. Periarticular soft tissue involvement.*

|  | Study ID | Overall risk of bias |
| --- | --- | --- |
| 1 | Bozgeyik 2014 | Low |
| 2 | Geyik 2002 | High |
| 3 | Gür 2003 | Low |

*Table S4-48. Bursitis.*

|  | Study ID | Overall risk of bias |
| --- | --- | --- |
| 1 | Bozgeyik 2014 | Low |
| 2 | Guler 2014 | Low |
| 3 | Hasanjani Roushan 2016 a | Low |
| 4 | Hasanjani Roushan 2004 | High |
| 5 | Kazak 2016 | High |
| 6 | Kutlu 2018 | Low |
| 7 | Mermut 2011 | High |
| 8 | Norton 1984 | High |
| 9 | Tasova 1999 | Low |
| 10 | Turan 2011 | High |
| 11 | Pourbagher 2006 | High |
| 12 | Paul 2017 | High |
| 13 | Sen 2019 | High |
| 14 | Cuzdan 2022 | High |

*Table S4-49. Tendinitis.*

|  | Study ID | Overall risk of bias |
| --- | --- | --- |
| 1 | Bozgeyik 2014 | Low |
| 2 | Guler 2014 | Low |

*Table S4-50. Tendinitis or bursitis.*

|  | Study ID | Overall risk of bias |
| --- | --- | --- |
| 1 | Mousa 1988 | Low |

*Table S4-51. Synovitis.*

|  | Study ID | Overall risk of bias |
| --- | --- | --- |
| 1 | Guler 2014 | Low |
| 2 | Mermut 2011 | High |

*Table S4-52. Enthesopathy.*

|  | Study ID | Overall risk of bias |
| --- | --- | --- |
| 1 | Heidari 2011 | High |

*Table S4-53. Enthesitis.*

|  | Study ID | Overall risk of bias |
| --- | --- | --- |
| 1 | Sen 2019 | High |

*Table S4-54. Avascular necrosis of femoral head.*

|  | Study ID | Overall risk of bias |
| --- | --- | --- |
| 1 | Benjamin 1992 | High |
| 2 | Çiftdogan 2020 | High |
| 3 | Pourbagher 2006 | High |

*Table S4-55. Genito-urinary system involvement.*

|  | Study ID | Overall risk of bias |
| --- | --- | --- |
| 1 | Artuk 2019 | High |
| 2 | Aygen 2002 | High |
| 3 | Benjamin 1992 | High |
| 4 | Bulut 2011 | High |
| 5 | Gür 2003 | Low |
| 6 | Hassan 2021 | High |
| 7 | Kursun 2013 | High |
| 8 | Kutlu 2018 | High |
| 9 | Ulug 2011 | High |
| 10 | Mermut 2011 | High |
| 11 | Yetkin 2005 | High |
| 12 | Turunç 2008 | High |
| 13 | Naz 2009 | High |
| 14 | Uluğ 2011 | High |
| 15 | Buzgan 2010 | High |
| 16 | Ertem 2004 | High |
| 17 | Sen 2019 | High |

*Table S4-56. Urinary system involvement.*

|  | Study ID | Overall risk of bias |
| --- | --- | --- |
| 1 | Khateeb 1990 | High |
| 2 | Mermut 2011 | High |
| 3 | Mousa 1988 | High |

*Table S4-57. Scrotal involvement.*

|  | Study ID | Overall risk of bias |
| --- | --- | --- |
| 1 | Bayram 1997 | High |

*Table S4-58. Orchitis.*

|  | Study ID | Overall risk of bias |
| --- | --- | --- |
| 1 | Abbasi 2020 | High |
| 2 | Akinci 2006 | High |
| 3 | Al-Dubooni 1986 | High |
| 4 | Bayram 1997 | High |
| 5 | Beheshti 2001 | High |
| 6 | Behzadnia 2018 | High |
| 7 | Celik 2023 | High |
| 8 | Ertek 2006 | High |
| 9 | Gozdas 2020 | High |
| 10 | Hashemi 2007 | High |
| 11 | Hizel 2007 | High |
| 12 | Kazak 2016 | High |
| 13 | Savas 2007 | High |
| 14 | Zia Sheikholeslami 2007 | High |
| 15 | Turan 2011 | High |
| 16 | Ulu-Kilic 2013 | High |
| 17 | Kayaaslan 2013 | High |
| 18 | Sagi 2017 | High |
| 19 | Hasanjani Roushan 2009 | High |
| 20 | Samra 1983 | High |
| 21 | Feiz 1978 | High |
| 22 | Türker 2014 | High |
| 23 | Torkaman 2017 | High |
| 24 | Naz 2016 | High |
| 25 | Najafi 2018 | High |
| 26 | Majzoobi 2018 | High |
| 27 | Sabbaghian 1974 | High |
| 28 | Demir 2021 | High |
| 29 | Hashemi 2018 | High |
| 30 | Karabay 2004 | High |
| 31 | Kadanali 2005 | High |
| 32 | Najafi 2011 | High |

*Table S4-59. Epididymitis.*

|  | Study ID | Overall risk of bias |
| --- | --- | --- |
| 1 | Akinci 2006 | High |
| 2 | Celik 2023 | High |

*Table S4-60. Epididymoorchitis.*

|  | Study ID | Overall risk of bias |
| --- | --- | --- |
| 1 | Abdi-Liae 2007 | High |
| 2 | Akinci 2006 | High |
| 3 | Al Nokhatha 2021 | High |
| 4 | Artuk 2019 | High |
| 5 | Aygen 2002 | High |
| 6 | Baykan 2019 | High |
| 7 | Bayram 1997 | High |
| 8 | Bukharie 2009 | High |
| 9 | Celen 2010 | High |
| 10 | Celik 2023 | High |
| 11 | Çirakli 2015 | High |
| 12 | Coskun 2009 | High |
| 13 | Dabaja-Younis 2023 | Low |
| 14 | Dal 2013 | High |
| 15 | Demirtuerk 2008 | High |
| 16 | Eini 2012 | High |
| 17 | Eroglu 2020 | High |
| 18 | Ertek 2006 | High |
| 19 | Fallah 2005 | High |
| 20 | Gonen 2014 | High |
| 21 | Gozdas 2020 | High |
| 22 | Gul 2014 | High |
| 23 | Guler 2014 | High |
| 24 | Gür 2003 | Low |
| 25 | Hadadi 2009 | High |
| 26 | Hasanjani Roushan 2016 a | Low |
| 27 | Hasanjani Roushan 2004 | High |
| 28 | Kayaaslan 2016 | High |
| 29 | Keyvanfar 2021 | High |
| 30 | Khateeb 1990 | High |
| 31 | Kokoglu 2006 | High |
| 32 | Kursun 2013 | High |
| 33 | Kurtaran 2012 | High |
| 34 | Kutlu 2018 | High |
| 35 | Ulug 2011 | High |
| 36 | Mohamed 1986 | High |
| 37 | Mousa 1988 | High |
| 38 | Najari 2018 | High |
| 39 | Namiduru 2003 | High |
| 40 | Özen 2022 | High |
| 41 | Savion 2023 | High |
| 42 | Tasbakan 2003 | High |
| 43 | Varikkodan 2024 | High |
| 44 | Yetkin 2005 | High |
| 45 | Kayaaslan 2013 | High |
| 46 | Hasanjani Roushan 2009 | High |
| 47 | Naz 2009 | High |
| 48 | Naz 2016 | High |
| 49 | Buzgan 2010 | High |
| 50 | Köse 2014 | High |
| 51 | Keramat 2009 | High |
| 52 | Keramat 2018 | High |
| 53 | Shehabi 1990 | High |
| 54 | Najafi 2018 | High |
| 55 | Sen 2019 | High |
| 56 | Alim 2015 | High |
| 57 | Najafi 2011 | High |

*Table S4-61. Testis hydrocele.*

|  | Study ID | Overall risk of bias |
| --- | --- | --- |
| 1 | Akinci 2006 | High |
| 2 | Celen 2010 | Low |
| 3 | Gozdas 2020 | High |
| 4 | Naz 2016 | High |

*Table S4-62. Testicular abscess.*

|  | Study ID | Overall risk of bias |
| --- | --- | --- |
| 1 | Akinci 2006 | High |
| 2 | Baykan 2019 | Low |
| 3 | Celik 2023 | High |
| 4 | Gozdas 2020 | High |
| 5 | Guler 2014 | High |
| 6 | Najafi 2011 | High |

*Table S4-63. Varicocele.*

|  | Study ID | Overall risk of bias |
| --- | --- | --- |
| 1 | Gozdas 2020 | High |

*Table S4-64. Testicular edema.*

|  | Study ID | Overall risk of bias |
| --- | --- | --- |
| 1 | Türker 2014 | High |
| 2 | Köse 2014 | High |

*Table S4-65. Prostatitis.*

|  | Study ID | Overall risk of bias |
| --- | --- | --- |
| 1 | Aygen 2002 | High |
| 2 | Gür 2003 | Low |
| 3 | Kursun 2013 | High |
| 4 | Kutlu 2018 | High |
| 5 | Buzgan 2010 | High |

*Table S4-66. Nephritis.*

|  | Study ID | Overall risk of bias |
| --- | --- | --- |
| 1 | Fallah 2005 | High |
| 2 | Gür 2003 | High |
| 3 | Khateeb 1990 | High |
| 4 | Mermut 2011 | High |
| 5 | Naz 2009 | High |
| 6 | Al Shaalan 2002 | High |
| 7 | Buzgan 2010 | High |

*Table S4-67. Nephrotic syndrome.*

|  | Study ID | Overall risk of bias |
| --- | --- | --- |
| 1 | Khateeb 1990 | High |

*Table S4-68. Renal failure.*

|  | Study ID | Overall risk of bias |
| --- | --- | --- |
| 1 | Khateeb 1990 | High |
| 2 | Buzgan 2010 | High |

*Table S4-69. Renal abscess.*

|  | Study ID | Overall risk of bias |
| --- | --- | --- |
| 1 | Gonen 2014 | High |

*Table S4-70. Kidney involvement.*

|  | Study ID | Overall risk of bias |
| --- | --- | --- |
| 1 | Buzgan 2010 | High |

*Table S4-71. Aspermia.*

|  | Study ID | Overall risk of bias |
| --- | --- | --- |
| 1 | Akinci 2006 | High |

*Table S4-72. Oligospermia.*

|  | Study ID | Overall risk of bias |
| --- | --- | --- |
| 1 | Akinci 2006 | High |

*Table S4-73. Permanent aspermia or oligospermia.*

|  | Study ID | Overall risk of bias |
| --- | --- | --- |
| 1 | Akinci 2006 | High |

*Table S4-74. Lymphadenopathy.*

|  | Study ID | Overall risk of bias |
| --- | --- | --- |
| 1 | Abd Alrheam 2015 | High |
| 2 | Abdi-Liae 2007 | High |
| 3 | Afify 2013 | High |
| 4 | Akdeniz 1998 | High |
| 5 | Al Sawafi 2023 | High |
| 6 | Al Shamahy 2001 | High |
| 7 | Al-Eissa 1993 | High |
| 8 | Al-Eissa1990 | High |
| 9 | Al-Freihi 1986 | High |
| 10 | Artuk 2019 | High |
| 11 | Aypak 2012 | High |
| 12 | Beheshti 2001 | High |
| 13 | Behzadnia 2018 | High |
| 14 | Benjamin 1992 | High |
| 15 | Bodur 2003 | High |
| 16 | Bozdemir 2017 | High |
| 17 | Bukharie 2009 | High |
| 18 | Caksen 2002 | High |
| 19 | Celen 2010 | High |
| 20 | Çiftdogan 2020 | High |
| 21 | Dabaja-Younis 2023 | High |
| 22 | El Bayoumy 2014 | High |
| 23 | El-Amin 2001 | High |
| 24 | El-Koumi 2013 | High |
| 25 | Ertek 2006 | High |
| 26 | Fallah 2005 | High |
| 27 | Fruchtman 2020 | High |
| 28 | Gonen 2014 | High |
| 29 | Gul 2014 | High |
| 30 | Guler 2014 | High |
| 31 | Gür 2003 | High |
| 32 | Kayaaslan 2016 | High |
| 33 | Kazak 2016 | High |
| 34 | Keyvanfar 2021 | High |
| 35 | Khateeb 1990 | High |
| 36 | Kutlu 2018 | High |
| 37 | Malik 1997 | High |
| 38 | Ulug 2011 | High |
| 39 | Mermut 2011 | High |
| 40 | Mohammad Saeed 2012 | High |
| 41 | Mousa 1988 | High |
| 42 | Mugahi 2014 | High |
| 43 | Namiduru 2003 | High |
| 44 | Özdem 2022 | High |
| 45 | Salman 2022 | High |
| 46 | Savas 2007 | High |
| 47 | Savion 2023 | High |
| 48 | Zia Sheikholeslami 2007 | High |
| 49 | Somily 2017 | High |
| 50 | Tasova 1999 | High |
| 51 | Tekin 2012 | High |
| 52 | Varikkodan 2024 | High |
| 53 | Kayaaslan 2013 | High |
| 54 | Samra 1983 | High |
| 55 | Naz 2009 | High |
| 56 | Rahil 2014 | High |
| 57 | Türker 2014 | High |
| 58 | Ertekin 2004 | High |
| 59 | Torkaman 2017 | High |
| 60 | Uluğ 2011 | High |
| 61 | Buzgan 2010 | High |
| 62 | Köse 2014 | High |
| 63 | Fanni 2013 | High |
| 64 | Kaman 2022 | High |
| 65 | Keramat 2009 | High |
| 66 | Kaya 2018 | High |
| 67 | Shehabi 1990 | High |
| 68 | Aktug-Demir 2014 | High |
| 69 | Najafi 2018 | High |
| 70 | El-Kafas 1999 | High |
| 71 | Zilberman 2022 | High |
| 72 | Mohamed 2018 | High |
| 73 | Paul 2017 | High |
| 74 | Kara 2019 | High |
| 75 | Salari 2003 | High |
| 76 | Alim 2015 | High |

*Table S4-75. Neck lymphadenopathy.*

|  | Study ID | Overall risk of bias |
| --- | --- | --- |
| 1 | Lazutkin 2018 | High |

*Table S4-76. Lymphadenitis.*

|  | Study ID | Overall risk of bias |
| --- | --- | --- |
| 1 | AlRashidi 2022 | High |
| 2 | Bukharie 2009 | High |
| 3 | Savion 2023 | High |
| 4 | Demir 2021 | High |

*Table S4-77. Lymphadenomegaly.*

|  | Study ID | Overall risk of bias |
| --- | --- | --- |
| 1 | Demirtuerk 2008 | High |
| 2 | Tasbakan 2003 | High |

*Table S4-78. Adenopathy.*

|  | Study ID | Overall risk of bias |
| --- | --- | --- |
| 1 | Al-Dubooni 1986 | High |
| 2 | El-Amin 2001 | High |
| 3 | Nabavi 2019 | High |
| 4 | Feiz 1978 | High |
| 5 | Hatami 2019 | High |

*Table S4-79. Parotitis.*

|  | Study ID | Overall risk of bias |
| --- | --- | --- |
| 1 | El-Amin 2001 | High |
| 2 | Samra 1983 | High |

*Table S4-80. Parotid abscess.*

|  | Study ID | Overall risk of bias |
| --- | --- | --- |
| 1 | Edathodu 2021 | High |

*Table S4-81. Spleen abscess.*

|  | Study ID | Overall risk of bias |
| --- | --- | --- |
| 1 | Dabaja-Younis 2023 | Low |
| 2 | Gonen 2014 | High |
| 3 | Gür 2003 | High |
| 4 | Özen 2022 | High |
| 5 | Salman 2022 | High |

*Table S4-82. Splenic infarct.*

|  | Study ID | Overall risk of bias |
| --- | --- | --- |
| 1 | Megged 2016 | High |

*Table S4-83. Nervous system involvement.*

|  | Study ID | Overall risk of bias |
| --- | --- | --- |
| 1 | Artuk 2019 | High |
| 2 | Aygen 2002 | High |
| 3 | Benbir 2013 | High |
| 4 | Bulut 2011 | High |
| 5 | Coskun 2004 | High |
| 6 | Ertek 2006 | High |
| 7 | Gul 2014 | High |
| 8 | Gür 2003 | High |
| 9 | Hasanjani Roushan 2004 | High |
| 10 | Ulug 2011 | High |
| 11 | Savion 2023 | High |
| 12 | Mousa 1988 | High |
| 13 | Ulu-Kilic 2013 | High |
| 14 | Turunç 2008 | High |
| 15 | Naz 2009 | High |

*Table S4-84. Central nervous system involvement.*

|  | Study ID | Overall risk of bias |
| --- | --- | --- |
| 1 | Çirakli 2015 | High |
| 2 | Dal 2013 | Low |
| 3 | Erat 2023 | High |
| 4 | Hasanjani Roushan 2016 a | Low |
| 5 | Khateeb 1990 | High |
| 6 | Mermut 2011 | High |
| 7 | Kutlu 2009 | High |
| 8 | Nabi 1992 | High |
| 9 | Buzgan 2010 | High |
| 10 | Edathodu 2021 | High |
| 11 | Paul 2017 | High |
| 12 | Lifeso 1985 | High |

*Table S4-85. Peripheral nervous system involvement.*

|  | Study ID | Overall risk of bias |
| --- | --- | --- |
| 1 | Khateeb 1990 | High |
| 2 | Kutlu 2009 | High |

*Table S4-86. Central nervous system and peripheral nervous system involvement.*

|  | Study ID | Overall risk of bias |
| --- | --- | --- |
| 1 | Khateeb 1990 | High |

*Table S4-87. Neurobrucellosis.*

|  | Study ID | Overall risk of bias |
| --- | --- | --- |
| 1 | Abdi-Liae 2007 | High |
| 2 | Al-Koubaisy 2011 | Low |
| 3 | Benjamin 1992 | High |
| 4 | Bodur 2003 | High |
| 5 | Bukharie 2009 | High |
| 6 | Bulut 2011 | High |
| 7 | Caksen 2002 | High |
| 8 | Dabaja-Younis 2023 | Low |
| 9 | Demirtuerk 2008 | High |
| 10 | El-Amin 2001 | High |
| 11 | Eroglu 2020 | High |
| 12 | Fallah 2005 | High |
| 13 | Hizel 2007 | High |
| 14 | Karaoglan 2008 | High |
| 15 | Kayaaslan 2016 | High |
| 16 | Kazak 2016 | High |
| 17 | Keyvanfar 2021 | Low |
| 18 | Khateeb 1990 | High |
| 19 | Kursun 2013 | High |
| 20 | Kurtaran 2012 | High |
| 21 | Kutlu 2018 | High |
| 22 | McLean 1992 | High |
| 23 | Megged 2016 | High |
| 24 | Özen 2022 | High |
| 25 | Salman 2022 | High |
| 26 | Tanir 2009 | High |
| 27 | Teke 2015 | High |
| 28 | Varikkodan 2024 | High |
| 29 | Yetkin 2005 | High |
| 30 | Abbasi 2012 | High |
| 31 | Kayaaslan 2013 | High |
| 32 | Turunç 2008 | High |
| 33 | Al Shaalan 2002 | High |
| 34 | Qasim 2020 | High |
| 35 | Yoldas 2015 | High |
| 36 | Buzgan 2010 | High |
| 37 | Edathodu 2021 | High |
| 38 | Köse 2014 | High |
| 39 | Keramat 2018 | High |
| 40 | Aktug-Demir 2014 | High |
| 41 | Najafi 2018 | High |
| 42 | Pourakbari 2019 | High |
| 43 | Vafaei 2019 | High |
| 44 | Biglarzadeh 2020 | High |
| 45 | Demir 2021 | High |
| 46 | Zilberman 2022 | High |
| 47 | Bayazit 2002 | High |
| 48 | Eskandari-Nasab 2014 | High |
| 49 | Sayin-Kutlu 2012 | High |
| 50 | Demiraslan 2009 | High |
| 51 | Guven 2013 | Low |
| 52 | Ranjbar 2009 | High |
| 53 | Ergun 2008 | High |

*Table S4-88. Toxic febrile neurobrucellosis.*

|  | Study ID | Overall risk of bias |
| --- | --- | --- |
| 1 | Buzgan 2010 | High |

*Table S4-89. Neuropsychiatric involvement.*

|  | Study ID | Overall risk of bias |
| --- | --- | --- |
| 1 | Hassan 2021 | High |
| 2 | Karaoglan 2008 | High |
| 3 | Malik 1997 | High |
| 4 | Mousa 1988 | High |
| 5 | Yetkin 2005 | High |
| 6 | Kiel 1987 | High |
| 7 | Uluğ 2011 | High |
| 8 | Ibrahim 2021 | High |
| 9 | Paul 2017 | High |
| 10 | Lifeso 1985 | High |
| 11 | Rezazadeh 2006 | High |

*Table S4-90. Psychiatric involvement.*

|  | Study ID | Overall risk of bias |
| --- | --- | --- |
| 1 | Khateeb 1990 | High |
| 2 | Mousa 1988 | High |
| 3 | Edathodu 2021 | High |
| 4 | Rahmanpour 2019 | High |

*Table S4-91. Insomnia.*

|  | Study ID | Overall risk of bias |
| --- | --- | --- |
| 1 | Khateeb 1990 | High |
| 2 | Malik 1997 | High |

*Table S4-92. Sleep disturbance.*

|  | Study ID | Overall risk of bias |
| --- | --- | --- |
| 1 | Zia Sheikholeslami 2007 | High |
| 2 | Rahmanpour 2019 | High |

*Table S4-93. Meningitis.*

|  | Study ID | Overall risk of bias |
| --- | --- | --- |
| 1 | Artuk 2019 | High |
| 2 | Aygen 2002 | High |
| 3 | Magableh 2007 | High |
| 4 | Bodur 2003 | High |
| 5 | Bukharie 2009 | High |
| 6 | Bulut 2011 | High |
| 7 | Celen 2010 | High |
| 8 | Demirtuerk 2008 | High |
| 9 | Eini 2012 | High |
| 10 | Ertek 2006 | High |
| 11 | Gonen 2014 | High |
| 12 | Gul 2014 | High |
| 13 | Guler 2014 | High |
| 14 | Gür 2003 | High |
| 15 | Hasanjani Roushan 2004 | High |
| 16 | Khateeb 1990 | High |
| 17 | Kokoglu 2006 | High |
| 18 | Kursun 2013 | High |
| 19 | McLean 1992 | High |
| 20 | Ulug 2011 | High |
| 21 | Mermut 2011 | High |
| 22 | Namiduru 2003 | High |
| 23 | Salman 2022 | High |
| 24 | Savion 2023 | High |
| 25 | Tanir 2009 | High |
| 26 | Teke 2015 | High |
| 27 | Yetkin 2005 | High |
| 28 | Mousa 1986 | High |
| 29 | Kutlu 2009 | High |
| 30 | Turunç 2008 | High |
| 31 | Türker 2014 | High |
| 32 | Torkaman 2017 | High |
| 33 | Shehabi 1990 | High |
| 34 | Hatami 2010 | High |
| 35 | Ayatollahi 2004 | High |
| 36 | Biglarzadeh 2020 | High |
| 37 | Demir 2021 | High |
| 38 | Eskandari-Nasab 2014 | High |
| 39 | Demiraslan 2009 | High |
| 40 | Ranjbar 2009 | High |

*Table S4-94. Meningoencephalitis.*

|  | Study ID | Overall risk of bias |
| --- | --- | --- |
| 1 | Aygen 2002 | High |
| 2 | Hadadi 2009 | High |
| 3 | Karaoglan 2008 | High |
| 4 | Tanir 2009 | High |
| 5 | Tasbakan 2003 | High |
| 6 | Teke 2015 | High |
| 7 | Demiraslan 2009 | High |
| 8 | Ranjbar 2009 | High |
| 9 | Sen 2019 | High |

*Table S4-95. Meningitis or meningoencephalitis.*

|  | Study ID | Overall risk of bias |
| --- | --- | --- |
| 1 | Kurtaran 2012 | High |
| 2 | Teke 2015 | High |
| 3 | Mousa 1986 | High |
| 4 | Buzgan 2010 | High |

*Table S4-96. Meningitis or encephalitis.*

|  | Study ID | Overall risk of bias |
| --- | --- | --- |
| 1 | Kaya 2018 | High |

*Table S4-97. Meningeal irritation findings.*

|  | Study ID | Overall risk of bias |
| --- | --- | --- |
| 1 | Kazak 2016 | High |
| 2 | Salman 2022 | High |
| 3 | Türker 2014 | High |

*Table S4-98. Meningomyelitis.*

|  | Study ID | Overall risk of bias |
| --- | --- | --- |
| 1 | Aygen 2002 | High |

*Table S4-99. Mening ischemic stroke.*

|  | Study ID | Overall risk of bias |
| --- | --- | --- |
| 1 | McLean 1992 | High |

*Table S4-100. Meningovascular involvement.*

|  | Study ID | Overall risk of bias |
| --- | --- | --- |
| 1 | Aygen 2002 | High |
| 2 | McLean 1992 | High |

*Table S4-101. Meningismus.*

|  | Study ID | Overall risk of bias |
| --- | --- | --- |
| 1 | Hashemi 2007 | High |

*Table S4-102. Cerebrovascular system involvement.*

|  | Study ID | Overall risk of bias |
| --- | --- | --- |
| 1 | Lifeso 1985 | High |

*Table S4-103. Encephalitis.*

|  | Study ID | Overall risk of bias |
| --- | --- | --- |
| 1 | Aygen 2002 | High |
| 2 | Benjamin 1992 | High |
| 3 | Gür 2003 | High |
| 4 | Karaoglan 2008 | High |
| 5 | Buzgan 2010 | High |

*Table S4-104. Encephalomyelitis.*

|  | Study ID | Overall risk of bias |
| --- | --- | --- |
| 1 | Kursun 2013 | High |

*Table S4-105. Acute cerebellar syndrome.*

|  | Study ID | Overall risk of bias |
| --- | --- | --- |
| 1 | Khateeb 1990 | High |

*Table S4-106. Cerebellar syndrome and pseudotumor cerebri.*

|  | Study ID | Overall risk of bias |
| --- | --- | --- |
| 1 | Khateeb 1990 | High |

*Table S4-107. Myelitis.*

|  | Study ID | Overall risk of bias |
| --- | --- | --- |
| 1 | Aygen 2002 | High |
| 2 | Karaoglan 2008 | High |
| 3 | Khateeb 1990 | High |
| 4 | Buzgan 2010 | High |
| 5 | Ranjbar 2009 | High |

*Table S4-108. Intra-cerebral haemorrhage.*

|  | Study ID | Overall risk of bias |
| --- | --- | --- |
| 1 | McLean 1992 | High |
| 2 | Ranjbar 2009 | High |

*Table S4-109. Subdural hematoma.*

|  | Study ID | Overall risk of bias |
| --- | --- | --- |
| 1 | McLean 1992 | High |
| 2 | Yetkin 2005 | High |

*Table S4-110. Sub-arachnoid haemorrhage.*

|  | Study ID | Overall risk of bias |
| --- | --- | --- |
| 1 | Artuk 2019 | High |
| 2 | McLean 1992 | High |
| 3 | Ranjbar 2009 | High |

*Table S4-111. Putamen infarction.*

|  | Study ID | Overall risk of bias |
| --- | --- | --- |
| 1 | Abbasi 2012 | High |

*Table S4-112. Brain or spinal cord paranchymal dysfunction.*

|  | Study ID | Overall risk of bias |
| --- | --- | --- |
| 1 | McLean 1992 | High |

*Table S4-113. Polyneuropathy.*

|  | Study ID | Overall risk of bias |
| --- | --- | --- |
| 1 | Benbir 2013 | High |
| 2 | Khateeb 1990 | High |

*Table S4-114. Radiculopathy.*

|  | Study ID | Overall risk of bias |
| --- | --- | --- |
| 1 | Hassan 2021 | High |
| 2 | McLean 1992 | High |

*Table S4-115. Polyradiculopathy.*

|  | Study ID | Overall risk of bias |
| --- | --- | --- |
| 1 | McLean 1992 | High |
| 2 | Ranjbar 2009 | High |

*Table S4-116. Polyradiculoneuritis.*

|  | Study ID | Overall risk of bias |
| --- | --- | --- |
| 1 | Aygen 2002 | High |
| 2 | Kursun 2013 | High |
| 3 | Yetkin 2005 | High |
| 4 | Buzgan 2010 | High |

*Table S4-117. Peripheral neuritis.*

|  | Study ID | Overall risk of bias |
| --- | --- | --- |
| 1 | Aygen 2002 | High |
| 2 | Magableh 2007 | High |

*Table S4-118. Peripheral neuropathy.*

|  | Study ID | Overall risk of bias |
| --- | --- | --- |
| 1 | Khateeb 1990 | High |
| 2 | McLean 1992 | High |
| 3 | Sanivar 2017 | High |

*Table S4-119. Axonal sensorimotor neuropathy.*

|  | Study ID | Overall risk of bias |
| --- | --- | --- |
| 1 | Kutlu 2009 | High |

*Table S4-120. Optic neuritis.*

|  | Study ID | Overall risk of bias |
| --- | --- | --- |
| 1 | Sen 2019 | High |

*Table S4-121. Superficial sensory loss.*

|  | Study ID | Overall risk of bias |
| --- | --- | --- |
| 1 | Benbir 2013 | High |

*Table S4-122. Deep sensory loss.*

|  | Study ID | Overall risk of bias |
| --- | --- | --- |
| 1 | Benbir 2013 | Low |

*Table S4-123. Brain atrophy.*

|  | Study ID | Overall risk of bias |
| --- | --- | --- |
| 1 | Benjamin 1992 | High |
| 2 | McLean 1992 | High |

*Table S4-124. Hydrocephalus.*

|  | Study ID | Overall risk of bias |
| --- | --- | --- |
| 1 | Karaoglan 2008 | High |
| 2 | McLean 1992 | High |
| 3 | Abbasi 2012 | High |

*Table S4-125. Brain abscess.*

|  | Study ID | Overall risk of bias |
| --- | --- | --- |
| 1 | Guler 2014 | High |
| 2 | Mermut 2011 | High |
| 3 | Buzgan 2010 | High |
| 4 | Kaya 2018 | High |
| 5 | Shehabi 1990 | High |

*Table S4-126. Brain demyelination.*

|  | Study ID | Overall risk of bias |
| --- | --- | --- |
| 1 | Karaoglan 2008 | High |

*Table S4-127. Cranial nerve involvement.*

|  | Study ID | Overall risk of bias |
| --- | --- | --- |
| 1 | Bodur 2003 | High |
| 2 | Karaoglan 2008 | High |
| 3 | Khateeb 1990 | High |
| 4 | Teke 2015 | High |
| 5 | Yetkin 2005 | High |
| 6 | Kayaaslan 2013 | High |
| 7 | Kutlu 2009 | High |
| 8 | Turunç 2008 | High |
| 9 | Guven 2013 | High |
| 10 | Ergun 2008 | High |

*Table S4-128. Cranial nerve palsy.*

|  | Study ID | Overall risk of bias |
| --- | --- | --- |
| 1 | Teke 2015 | High |
| 2 | Turunç 2008 | High |
| 3 | Karakurt 2023 | High |

*Table S4-129. Nervus Abducens involvement.*

|  | Study ID | Overall risk of bias |
| --- | --- | --- |
| 1 | Bodur 2003 | High |
| 2 | McLean 1992 | High |
| 3 | Teke 2015 | High |
| 4 | Yetkin 2005 | High |
| 5 | Kutlu 2009 | High |
| 6 | Turunç 2008 | High |
| 7 | Demiraslan 2009 | High |
| 8 | Guven 2013 | High |

*Table S4-130. Facial nerve involvement.*

|  | Study ID | Overall risk of bias |
| --- | --- | --- |
| 1 | Guven 2013 | High |

*Table S4-131. Vestibulocochlear nerve involvement.*

|  | Study ID | Overall risk of bias |
| --- | --- | --- |
| 1 | Bodur 2003 | High |
| 2 | Guven 2013 | High |

*Table S4-132. Facial paralysis.*

|  | Study ID | Overall risk of bias |
| --- | --- | --- |
| 1 | Bodur 2003 | High |
| 2 | Guven 2013 | High |

*Table S4-133. Bell’s palsy.*

|  | Study ID | Overall risk of bias |
| --- | --- | --- |
| 1 | Fruchtman 2020 | High |
| 2 | Hasanjani Roushan 2004 | High |

*Table S4-134. Hearing loss.*

|  | Study ID | Overall risk of bias |
| --- | --- | --- |
| 1 | Artuk 2019 | High |
| 2 | Bodur 2003 | High |
| 3 | Coskun 2004 | High |
| 4 | Guler 2014 | High |
| 5 | McLean 1992 | High |
| 6 | Teke 2015 | High |
| 7 | Edathodu 2021 | High |
| 8 | Bayazit 2002 | High |
| 9 | Kaygusuz 2005 | High |
| 10 | Guven 2013 | High |
| 11 | Ranjbar 2009 | High |

*Table S4-135. Tinnitus.*

|  | Study ID | Overall risk of bias |
| --- | --- | --- |
| 1 | Edathodu 2021 | High |
| 2 | Kaygusuz 2005 | High |
| 3 | Ergun 2008 | High |

*Table S4-136. Papilledema.*

|  | Study ID | Overall risk of bias |
| --- | --- | --- |
| 1 | Karaoglan 2008 | High |
| 2 | Khateeb 1990 | High |
| 3 | McLean 1992 | High |
| 4 | Sungur 2009 | High |
| 5 | Yetkin 2005 | High |
| 6 | Kutlu 2009 | High |
| 7 | Ranjbar 2009 | High |
| 8 | Karakurt 2023 | High |
| 9 | Ergun 2008 | High |

*Table S4-137. Paralysis.*

|  | Study ID | Overall risk of bias |
| --- | --- | --- |
| 1 | Khateeb 1990 | High |
| 2 | McLean 1992 | High |
| 3 | Mohammad Saeed 2012 | High |
| 4 | Ranjbar 2009 | High |

*Table S4-138. Myelopathy.*

|  | Study ID | Overall risk of bias |
| --- | --- | --- |
| 1 | McLean 1992 | High |
| 2 | Ranjbar 2009 | High |

*Table S4-139. Ataxia.*

|  | Study ID | Overall risk of bias |
| --- | --- | --- |
| 1 | El-Amin 2001 | High |
| 2 | McLean 1992 | High |
| 3 | Teke 2015 | High |
| 4 | Al Shaalan 2002 | High |
| 5 | Guven 2013 | High |
| 6 | Ranjbar 2009 | High |
| 7 | Ergun 2008 | High |

*Table S4-140. Depression.*

|  | Study ID | Overall risk of bias |
| --- | --- | --- |
| 1 | Celen 2010 | High |
| 2 | El Bayoumy 2014 | High |
| 3 | Gür 2003 | High |
| 4 | Karaoglan 2008 | High |
| 5 | Kazak 2016 | High |
| 6 | Khateeb 1990 | High |
| 7 | Ulug 2011 | High |
| 8 | Nabavi 2019 | High |
| 9 | Savas 2007 | High |
| 10 | Naz 2009 | High |
| 11 | Kaya 2018 | High |
| 12 | Pourbagher 2006 | High |
| 13 | Hatami 2019 | High |
| 14 | Ranjbar 2009 | High |
| 15 | Rezazadeh 2006 | High |

*Table S4-141. Major depression.*

|  | Study ID | Overall risk of bias |
| --- | --- | --- |
| 1 | Mermut 2011 | High |

*Table S4-142. Depression and/or irritability.*

|  | Study ID | Overall risk of bias |
| --- | --- | --- |
| 1 | Mohamed 1986 | High |

*Table S4-143. Focal neurological deficits.*

|  | Study ID | Overall risk of bias |
| --- | --- | --- |
| 1 | Abbasi 2012 | High |

*Table S4-144. Status epilepticus.*

|  | Study ID | Overall risk of bias |
| --- | --- | --- |
| 1 | Megged 2016 | High |
| 2 | Buzgan 2010 | High |

*Table S4-145. Neurological disorder.*

|  | Study ID | Overall risk of bias |
| --- | --- | --- |
| 1 | Kayaaslan 2016 | High |
| 2 | Kayaaslan 2013 | High |

*Table S4-146. Cardiovascular system involvement.*

|  | Study ID | Overall risk of bias |
| --- | --- | --- |
| 1 | Artuk 2019 | High |
| 2 | Bulut 2011 | High |
| 3 | Çirakli 2015 | High |
| 4 | Gul 2014 | High |
| 5 | Khateeb 1990 | High |
| 6 | Ulug 2011 | High |
| 7 | Mermut 2011 | High |
| 8 | Mousa 1988 | High |
| 9 | Uluğ 2011 | High |
| 10 | Buzgan 2010 | High |
| 11 | Sen 2019 | High |

*Table S4-147. Cardiac involvement.*

|  | Study ID | Overall risk of bias |
| --- | --- | --- |
| 1 | Hassan 2021 | High |
| 2 | Lazutkin 2018 | High |
| 3 | Savion 2023 | High |
| 4 | Varikkodan 2024 | High |
| 5 | Nabi 1992 | High |

*Table S4-148. Carditis.*

|  | Study ID | Overall risk of bias |
| --- | --- | --- |
| 1 | Magableh 2007 | High |
| 2 | Celen 2010 | High |
| 3 | Gür 2003 | Low |

*Table S4-149. Endocarditis.*

|  | Study ID | Overall risk of bias |
| --- | --- | --- |
| 1 | Abdi-Liae 2007 | High |
| 2 | Al Hashan 2017 | High |
| 3 | Al Nokhatha 2021 | High |
| 4 | Al-Freihi 1986 | High |
| 5 | Artuk 2019 | High |
| 6 | Aygen 2002 | High |
| 7 | Benjamin 1992 | High |
| 8 | Bukharie 2009 | High |
| 9 | Dabaja-Younis 2023 | Low |
| 10 | Ertek 2006 | High |
| 11 | Esmailpour 2010 | High |
| 12 | Fallah 2005 | High |
| 13 | Gonen 2014 | High |
| 14 | Guler 2014 | High |
| 15 | Hadadi 2009 | High |
| 16 | Hasanjani Roushan 2004 | High |
| 17 | Hizel 2007 | High |
| 18 | Kazak 2016 | High |
| 19 | Keyvanfar 2021 | Low |
| 20 | Khateeb 1990 | High |
| 21 | Kokoglu 2006 | High |
| 22 | Kursun 2013 | High |
| 23 | Kutlu 2018 | Low |
| 24 | Lazutkin 2018 | High |
| 25 | Megged 2016 | High |
| 26 | Mermut 2011 | High |
| 27 | Mohamed 1986 | High |
| 28 | Mousa 1988 | High |
| 29 | Najari 2018 | High |
| 30 | Savion 2023 | High |
| 31 | Varikkodan 2024 | High |
| 32 | Türker 2014 | High |
| 33 | Buzgan 2010 | High |
| 34 | Köse 2014 | High |
| 35 | Kaya 2018 | High |
| 36 | Najafi 2018 | High |
| 37 | Ayatollahi 2004 | High |
| 38 | Biglarzadeh 2020 | High |
| 39 | Demir 2021 | High |
| 40 | Zilberman 2022 | High |
| 41 | Eskandari-Nasab 2014 | High |
| 42 | Sen 2019 | High |

*Table S4-150. Endocarditis or vegetation.*

|  | Study ID | Overall risk of bias |
| --- | --- | --- |
| 1 | Edathodu 2021 | High |

*Table S4-151. Myocarditis.*

|  | Study ID | Overall risk of bias |
| --- | --- | --- |
| 1 | Al-Eissa1990 | High |
| 2 | Khateeb 1990 | High |
| 3 | Lazutkin 2018 | High |
| 4 | Al Shaalan 2002 | High |

*Table S4-152. Myocardial infarction.*

|  | Study ID | Overall risk of bias |
| --- | --- | --- |
| 1 | Khateeb 1990 | High |

*Table S4-153. Pericarditis.*

|  | Study ID | Overall risk of bias |
| --- | --- | --- |
| 1 | Khateeb 1990 | High |
| 2 | Kaya 2018 | High |

*Table S4-154. Perimyocarditis.*

|  | Study ID | Overall risk of bias |
| --- | --- | --- |
| 1 | Dabaja-Younis 2023 | Low |

*Table S4-155. Aortitis and aortic rupture.*

|  | Study ID | Overall risk of bias |
| --- | --- | --- |
| 1 | Dabaja-Younis 2023 | Low |

*Table S4-156. Aortic thrombus.*

|  | Study ID | Overall risk of bias |
| --- | --- | --- |
| 1 | Sen 2019 | High |

*Table S4-157. Thrombophlebitis.*

|  | Study ID | Overall risk of bias |
| --- | --- | --- |
| 1 | Artuk 2019 | High |
| 2 | Ertek 2006 | High |
| 3 | Gul 2014 | High |
| 4 | Buzgan 2010 | High |
| 5 | Najafi 2018 | High |

*Table S4-158. Septic endophlebitis.*

|  | Study ID | Overall risk of bias |
| --- | --- | --- |
| 1 | Memish 2001 | High |

*Table S4-159. Endovascular infection.*

|  | Study ID | Overall risk of bias |
| --- | --- | --- |
| 1 | Eroglu 2020 | High |

*Table S4-160. Deep vein thrombosis.*

|  | Study ID | Overall risk of bias |
| --- | --- | --- |
| 1 | Mermut 2011 | High |
| 2 | Keramat 2018 | High |

*Table S4-161. Capillary leak syndrome.*

|  | Study ID | Overall risk of bias |
| --- | --- | --- |
| 1 | Artuk 2019 | High |
| 2 | Gul 2014 | High |

*Table S4-162. Sepsis.*

|  | Study ID | Overall risk of bias |
| --- | --- | --- |
|  | Gür 2003 | High |

*Table S4-163. Hematologic involvement.*

|  | Study ID | Overall risk of bias |
| --- | --- | --- |
| 1 | Akbayram 2015 | High |
| 2 | Artuk 2019 | High |
| 3 | Bulut 2011 | High |
| 4 | Dal 2013 | High |
| 5 | Ertek 2006 | High |
| 6 | Fruchtman 2020 | High |
| 7 | Gul 2014 | High |
| 8 | Guler 2014 | High |
| 9 | Gür 2003 | High |
| 10 | Khateeb 1990 | High |
| 11 | Ulug 2011 | High |
| 12 | Mermut 2011 | High |
| 13 | Shalev 1994 | High |
| 14 | Naz 2009 | High |
| 15 | Uluğ 2011 | High |
| 16 | Buzgan 2010 | High |
| 17 | Kaman 2022 | High |
| 18 | Kuruoglu 2023 | High |
| 19 | Citak 2010 | High |
| 20 | Sen 2019 | High |
| 21 | Rezazadeh 2006 | High |

*Table S4-164. Leukopenia.*

|  | Study ID | Overall risk of bias |
| --- | --- | --- |
| 1 | Abdi-Liae 2007 | High |
| 2 | Afify 2013 | High |
| 3 | Akbayram 2015 | High |
| 4 | Akdeniz 1998 | High |
| 5 | Al Shamahy 2001 | High |
| 6 | Al-Eissa 1993 | High |
| 7 | Al-Eissa1990 | High |
| 8 | Artuk 2019 | High |
| 9 | Aygen 2002 | High |
| 10 | Behzadnia 2018 | High |
| 11 | Besharat 2010 | High |
| 12 | Bukharie 2009 | High |
| 13 | Bulut 2011 | High |
| 14 | Caksen 2002 | High |
| 15 | Celen 2010 | High |
| 16 | Çiftdogan 2020 | High |
| 17 | Çirakli 2015 | High |
| 18 | Demirtuerk 2008 | High |
| 19 | Eini 2012 | High |
| 20 | El-Amin 2001 | High |
| 21 | El-Koumi 2013 | High |
| 22 | Eroglu 2020 | High |
| 23 | Fruchtman 2020 | High |
| 24 | Gonen 2014 | High |
| 25 | Guler 2014 | High |
| 26 | Gür 2003 | High |
| 27 | Hadadi 2009 | High |
| 28 | Hasanjani Roushan 2004 | High |
| 29 | Issa 1999 | High |
| 30 | Kayaaslan 2016 | High |
| 31 | Kokoglu 2006 | High |
| 32 | Kutlu 2018 | High |
| 33 | Ulug 2011 | High |
| 34 | Mermut 2011 | High |
| 35 | Mousa 1988 | High |
| 36 | Mugahi 2014 | High |
| 37 | Najari 2018 | High |
| 38 | Namiduru 2003 | High |
| 39 | Okur 2012 | High |
| 40 | Özdem 2022 | High |
| 41 | Özen 2022 | High |
| 42 | Parlak 2015 | High |
| 43 | Rashad 2019 | High |
| 44 | Roushan 2005 | High |
| 45 | Salman 2022 | High |
| 46 | Shahnaz 2007 | High |
| 47 | Somily 2017 | High |
| 48 | Tanir 2009 | High |
| 49 | Ulu-Kilic 2013 | High |
| 50 | Varikkodan 2024 | High |
| 51 | Kayaaslan 2013 | High |
| 52 | Samra 1983 | High |
| 53 | Turunç 2008 | High |
| 54 | Naz 2009 | High |
| 55 | Al Shaalan 2002 | High |
| 56 | Rahil 2014 | High |
| 57 | Qasim 2020 | High |
| 58 | Türker 2014 | High |
| 59 | Naz 2016 | High |
| 60 | Uluğ 2011 | High |
| 61 | Yoldas 2015 | High |
| 62 | Buzgan 2010 | High |
| 63 | Köse 2014 | High |
| 64 | Fanni 2013 | High |
| 65 | Kaman 2022 | High |
| 66 | Kaya 2018 | High |
| 67 | Aktug-Demir 2014 | High |
| 68 | Najafi 2018 | Low |
| 69 | Pourakbari 2019 | High |
| 70 | El-Kafas 1999 | High |
| 71 | Aypak 2015 | High |
| 72 | Gholami 2012 | High |
| 73 | Shimol 2012 | High |
| 74 | Hashemi 2018 | High |
| 75 | Karakurt 2023 | High |
| 76 | Paul 2017 | High |
| 77 | Sen 2019 | High |

*Table S4-165. Leukocytosis.*

|  | Study ID | Overall risk of bias |
| --- | --- | --- |
| 1 | Abdi-Liae 2007 | High |
| 2 | Afify 2013 | High |
| 3 | Akbayram 2015 | High |
| 4 | Akdeniz 1998 | High |
| 5 | Al Nokhatha 2021 | High |
| 6 | Al Shamahy 2001 | High |
| 7 | Al-Eissa 1993 | High |
| 8 | Al-Freihi 1986 | High |
| 9 | Artuk 2019 | High |
| 10 | Aygen 2002 | High |
| 11 | Aypak 2012 | High |
| 12 | Behzadnia 2018 | High |
| 13 | Besharat 2010 | High |
| 14 | Bukharie 2009 | High |
| 15 | Caksen 2002 | High |
| 16 | Celen 2010 | High |
| 17 | Çiftdogan 2020 | High |
| 18 | Conkar 2018 | High |
| 19 | Demirtuerk 2008 | High |
| 20 | Eini 2012 | High |
| 21 | El-Koumi 2013 | High |
| 22 | Eroglu 2020 | High |
| 23 | Gonen 2014 | High |
| 24 | Guler 2014 | High |
| 25 | Gür 2003 | High |
| 26 | Hadadi 2009 | High |
| 27 | Hasanjani Roushan 2004 | High |
| 28 | Hassan 2021 | High |
| 29 | Kayaaslan 2016 | High |
| 30 | Kokoglu 2006 | High |
| 31 | Kutlu 2018 | High |
| 32 | Ulug 2011 | High |
| 33 | Mermut 2011 | High |
| 34 | Mousa 1988 | High |
| 35 | Mugahi 2014 | High |
| 36 | Najari 2018 | High |
| 37 | Namiduru 2003 | High |
| 38 | Parlak 2015 | High |
| 39 | Roushan 2005 | High |
| 40 | Shahnaz 2007 | High |
| 41 | Somily 2017 | High |
| 42 | Tanir 2009 | High |
| 43 | Varikkodan 2024 | High |
| 44 | Kayaaslan 2013 | High |
| 45 | Naz 2009 | High |
| 46 | Al Shaalan 2002 | High |
| 47 | Rahil 2014 | High |
| 48 | Qasim 2020 | High |
| 49 | Naz 2016 | High |
| 50 | Uluğ 2011 | High |
| 51 | Yoldas 2015 | High |
| 52 | Buzgan 2010 | High |
| 53 | Köse 2014 | High |
| 54 | Fanni 2013 | High |
| 55 | Aktug-Demir 2014 | High |
| 56 | Najafi 2018 | Low |
| 57 | Pourakbari 2019 | High |
| 58 | Gholami 2012 | High |
| 59 | Shimol 2012 | High |

*Table S4-166. Lymphopenia.*

|  | Study ID | Overall risk of bias |
| --- | --- | --- |
| 1 | Akbayram 2015 | High |
| 2 | Al-Eissa 1993 | High |
| 3 | Al-Eissa1990 | High |
| 4 | Mousa 1988 | High |
| 5 | Özen 2022 | High |
| 6 | Parlak 2015 | High |
| 7 | Somily 2017 | High |
| 8 | Kaman 2022 | High |

*Table S4-167. Lymphocytosis.*

|  | Study ID | Overall risk of bias |
| --- | --- | --- |
| 1 | Afify 2013 | High |
| 2 | Al Shamahy 2001 | High |
| 3 | Al-Eissa 1993 | High |
| 4 | Aygen 2002 | High |
| 5 | Besharat 2010 | High |
| 6 | Bukharie 2009 | High |
| 7 | Eini 2012 | High |
| 8 | El-Koumi 2013 | High |
| 9 | Mousa 1988 | High |
| 10 | Savas 2007 | High |
| 11 | Somily 2017 | High |
| 12 | Tasova 1999 | High |
| 13 | Turunç 2008 | High |
| 14 | Rahil 2014 | High |
| 15 | Pourakbari 2019 | High |
| 16 | Paul 2017 | High |

*Table S4-168. Neutropenia.*

|  | Study ID | Overall risk of bias |
| --- | --- | --- |
| 1 | Akbayram 2015 | High |
| 2 | Al-Eissa 1993 | High |
| 3 | Al-Eissa1990 | High |
| 4 | Al-Freihi 1986 | High |
| 5 | Fruchtman 2020 | High |
| 6 | Mousa 1988 | High |
| 7 | Özen 2022 | High |
| 8 | Parlak 2015 | High |
| 9 | Somily 2017 | High |
| 10 | Kaman 2022 | High |

*Table S4-169. Neutrophilia.*

|  | Study ID | Overall risk of bias |
| --- | --- | --- |
| 1 | Al-Eissa 1993 | High |
| 2 | Eini 2012 | High |
| 3 | Mousa 1988 | High |
| 4 | Somily 2017 | High |
| 5 | Rahil 2014 | High |

*Table S4-170. Eosinophilia.*

|  | Study ID | Overall risk of bias |
| --- | --- | --- |
| 1 | Akbayram 2015 | High |
| 2 | Özen 2022 | High |
| 3 | Parlak 2015 | High |

*Table S4-171. Lymphomonocytosis.*

|  | Study ID | Overall risk of bias |
| --- | --- | --- |
| 1 | Akdeniz 1998 | High |
| 2 | Hadadi 2009 | High |
| 3 | Kokoglu 2006 | High |
| 4 | Mermut 2011 | High |
| 5 | Namiduru 2003 | High |
| 6 | Buzgan 2010 | High |

*Table S4-172. Monocytopenia.*

|  | Study ID | Overall risk of bias |
| --- | --- | --- |
| 1 | Somily 2017 | High |

*Table S4-173. Monocytosis.*

|  | Study ID | Overall risk of bias |
| --- | --- | --- |
| 1 | Özen 2022 | High |
| 2 | Somily 2017 | High |
| 3 | Al Shaalan 2002 | High |

*Table S4-174. Lymphohistiocytosis.*

|  | Study ID | Overall risk of bias |
| --- | --- | --- |
| 1 | Akbayram 2015 | High |

*Table S4-175. Anemia.*

|  | Study ID | Overall risk of bias |
| --- | --- | --- |
| 1 | Abdi-Liae 2007 | High |
| 2 | Afify 2013 | High |
| 3 | Akbayram 2015 | High |
| 4 | Akdeniz 1998 | High |
| 5 | Al Nokhatha 2021 | High |
| 6 | Al Sawafi 2023 | High |
| 7 | Al Shamahy 2001 | High |
| 8 | Al-Dubooni 1986 | High |
| 9 | Al-Eissa 1993 | High |
| 10 | Al-Eissa1990 | High |
| 11 | Al-Freihi 1986 | High |
| 12 | Artuk 2019 | High |
| 13 | Aygen 2002 | High |
| 14 | Aypak 2012 | High |
| 15 | Behzadnia 2018 | High |
| 16 | Besharat 2010 | High |
| 17 | Bukharie 2009 | High |
| 18 | Bulut 2011 | High |
| 19 | Caksen 2002 | High |
| 20 | Çirakli 2015 | High |
| 21 | Dabaja-Younis 2023 | High |
| 22 | Demirtuerk 2008 | High |
| 23 | Eini 2012 | High |
| 24 | El Bayoumy 2014 | High |
| 25 | El-Koumi 2013 | High |
| 26 | Eroglu 2020 | High |
| 27 | Fallah 2005 | High |
| 28 | Fruchtman 2020 | High |
| 29 | Golsha 2011 | High |
| 30 | Gonen 2014 | High |
| 31 | Gul 2014 | High |
| 32 | Guler 2014 | High |
| 33 | Gür 2003 | High |
| 34 | Hadadi 2009 | High |
| 35 | Hasanjani Roushan 2004 | High |
| 36 | Hashemi 2007 | High |
| 37 | Issa 1999 | High |
| 38 | Kayaaslan 2016 | High |
| 39 | Keyvanfar 2021 | High |
| 40 | Khateeb 1990 | High |
| 41 | Kokoglu 2006 | High |
| 42 | Kutlu 2018 | High |
| 43 | Ulug 2011 | High |
| 44 | Mermut 2011 | High |
| 45 | Mugahi 2014 | High |
| 46 | Najari 2018 | High |
| 47 | Namiduru 2003 | High |
| 48 | Okur 2012 | High |
| 49 | Özdem 2022 | High |
| 50 | Özen 2022 | High |
| 51 | Parlak 2015 | High |
| 52 | Roushan 2005 | High |
| 53 | Salman 2022 | High |
| 54 | Shahnaz 2007 | High |
| 55 | Somily 2017 | High |
| 56 | Tanir 2009 | High |
| 57 | Tasova 1999 | High |
| 58 | Turan 2011 | High |
| 59 | Ulu-Kilic 2013 | High |
| 60 | Varikkodan 2024 | High |
| 61 | Kayaaslan 2013 | High |
| 62 | Feiz 1978 | High |
| 63 | Turunç 2008 | High |
| 64 | Naz 2009 | High |
| 65 | Opawoye 1994 | High |
| 66 | Al Shaalan 2002 | High |
| 67 | Nabi 1992 | High |
| 68 | Rahil 2014 | High |
| 69 | Türker 2014 | High |
| 70 | Ertekin 2004 | High |
| 71 | Torkaman 2017 | High |
| 72 | Naz 2016 | High |
| 73 | Uluğ 2011 | High |
| 74 | Yoldas 2015 | High |
| 75 | Buzgan 2010 | High |
| 76 | Köse 2014 | High |
| 77 | Fanni 2013 | High |
| 78 | Kaman 2022 | High |
| 79 | Kaya 2018 | High |
| 80 | Aktug-Demir 2014 | High |
| 81 | Najafi 2018 | High |
| 82 | Pourbagher 2006 | High |
| 83 | Pourakbari 2019 | High |
| 84 | El-Kafas 1999 | High |
| 85 | Sabbaghian 1974 | High |
| 86 | Kuruoglu 2023 | High |
| 87 | Aypak 2015 | High |
| 88 | Gholami 2012 | High |
| 89 | Shimol 2012 | High |
| 90 | Karakurt 2023 | High |
| 91 | Paul 2017 | High |
| 92 | Sen 2019 | High |
| 93 | Ahmadinejad 2016 | High |
| 94 | Kadanali 2005 | High |

*Table S4-176. Hemolytic anemia.*

|  | Study ID | Overall risk of bias |
| --- | --- | --- |
| 1 | Gür 2003 | High |

*Table S4-177. Thrombocytopenia.*

|  | Study ID | Overall risk of bias |
| --- | --- | --- |
| 1 | Abdi-Liae 2007 | High |
| 2 | Akbayram 2015 | High |
| 3 | Akdeniz 1998 | High |
| 4 | Al Shamahy 2001 | High |
| 5 | Al-Eissa 1993 | High |
| 6 | Al-Eissa1990 | High |
| 7 | Artuk 2019 | High |
| 8 | Aygen 2002 | High |
| 9 | Bukharie 2009 | High |
| 10 | Bulut 2011 | High |
| 11 | Celen 2010 | High |
| 12 | Çiftdogan 2020 | High |
| 13 | Çirakli 2015 | High |
| 14 | Dabaja-Younis 2023 | High |
| 15 | Demirtuerk 2008 | High |
| 16 | El-Amin 2001 | High |
| 17 | Eroglu 2020 | High |
| 18 | Ertek 2006 | High |
| 19 | Fallah 2005 | High |
| 20 | Fruchtman 2020 | High |
| 21 | Gonen 2014 | High |
| 22 | Gul 2014 | High |
| 23 | Guler 2014 | High |
| 24 | Gür 2003 | High |
| 25 | Hadadi 2009 | High |
| 26 | Hasanjani Roushan 2004 | High |
| 27 | Issa 1999 | High |
| 28 | Kayaaslan 2016 | High |
| 29 | Kokoglu 2006 | High |
| 30 | Kutlu 2018 | High |
| 31 | Ulug 2011 | High |
| 32 | Mermut 2011 | High |
| 33 | Mousa 1988 | High |
| 34 | Mugahi 2014 | High |
| 35 | Najari 2018 | High |
| 36 | Namiduru 2003 | High |
| 37 | Okur 2012 | High |
| 38 | Özdem 2022 | High |
| 39 | Özen 2022 | High |
| 40 | Parlak 2015 | High |
| 41 | Roushan 2005 | High |
| 42 | Salman 2022 | High |
| 43 | Shahnaz 2007 | High |
| 44 | Somily 2017 | High |
| 45 | Tanir 2009 | High |
| 46 | Ulu-Kilic 2013 | High |
| 47 | Varikkodan 2024 | High |
| 48 | Kayaaslan 2013 | High |
| 49 | Akbayram 2011 | High |
| 50 | Turunç 2008 | High |
| 51 | Naz 2009 | High |
| 52 | Al Shaalan 2002 | High |
| 53 | Rahil 2014 | High |
| 54 | Qasim 2020 | High |
| 55 | Türker 2014 | High |
| 56 | Ertekin 2004 | High |
| 57 | Naz 2016 | High |
| 58 | Uluğ 2011 | High |
| 59 | Yoldas 2015 | High |
| 60 | Buzgan 2010 | High |
| 61 | Köse 2014 | High |
| 62 | Fanni 2013 | High |
| 63 | Kaman 2022 | High |
| 64 | Kaya 2018 | High |
| 65 | Aktug-Demir 2014 | High |
| 66 | Najafi 2018 | Low |
| 67 | Pourakbari 2019 | High |
| 68 | El-Kafas 1999 | High |
| 69 | Kuruoglu 2023 | High |
| 70 | Citak 2010 | High |
| 71 | Aypak 2015 | High |
| 72 | Karakurt 2023 | High |
| 73 | Paul 2017 | High |
| 74 | Sen 2019 | High |

*Table S4-178. Thrombocytosis.*

|  | Study ID | Overall risk of bias |
| --- | --- | --- |
| 1 | Abdi-Liae 2007 | High |
| 2 | Akbayram 2015 | High |
| 3 | Guler 2014 | High |
| 4 | Hasanjani Roushan 2004 | High |
| 5 | Kutlu 2018 | High |
| 6 | Najari 2018 | High |
| 7 | Somily 2017 | High |
| 8 | Tanir 2009 | High |
| 9 | Varikkodan 2024 | High |
| 10 | Naz 2016 | High |
| 11 | Yoldas 2015 | High |
| 12 | Fanni 2013 | High |
| 13 | Pourakbari 2019 | High |
| 14 | Aypak 2015 | High |

*Table S4-179. Pancytopenia.*

|  | Study ID | Overall risk of bias |
| --- | --- | --- |
| 1 | Abdi-Liae 2007 | High |
| 2 | Afify 2013 | High |
| 3 | Akbayram 2011 | High |
| 4 | Akbayram 2015 | High |
| 5 | Akdeniz 1998 | High |
| 6 | Al-Eissa 1993 | High |
| 7 | Al-Eissa1990 | High |
| 8 | Artuk 2019 | High |
| 9 | Besharat 2010 | High |
| 10 | Bukharie 2009 | High |
| 11 | Çirakli 2015 | High |
| 12 | El-Koumi 2013 | High |
| 13 | Ertek 2006 | High |
| 14 | Fruchtman 2020 | High |
| 15 | Gonen 2014 | High |
| 16 | Guler 2014 | High |
| 17 | Gür 2003 | High |
| 18 | Hassan 2021 | High |
| 19 | Issa 1999 | High |
| 20 | Karli 2015 | High |
| 21 | Kayaaslan 2016 | High |
| 22 | Khateeb 1990 | High |
| 23 | Ulug 2011 | High |
| 24 | Mermut 2011 | High |
| 25 | Mousa 1988 | High |
| 26 | Namiduru 2003 | High |
| 27 | Okur 2012 | High |
| 28 | Parlak 2015 | High |
| 29 | Salman 2022 | High |
| 30 | Savion 2023 | High |
| 31 | Shalev 1994 | High |
| 32 | Somily 2017 | High |
| 33 | Naz 2009 | High |
| 34 | Türker 2014 | High |
| 35 | Ertekin 2004 | High |
| 36 | Uluğ 2011 | High |
| 37 | Yoldas 2015 | High |
| 38 | Buzgan 2010 | High |
| 39 | Fanni 2013 | High |
| 40 | Kaman 2022 | High |
| 41 | Kaya 2018 | High |
| 42 | Pourakbari 2019 | High |
| 43 | El-Kafas 1999 | High |
| 44 | Citak 2010 | High |
| 45 | Aypak 2015 | High |
| 46 | Sari 2008 | High |
| 47 | Paul 2017 | High |
| 48 | Karakukcu 2004 | High |
| 49 | Sen 2019 | High |

*Table S4-180. Cytopenia.*

|  | Study ID | Overall risk of bias |
| --- | --- | --- |
| 1 | Özen 2022 | High |
| 2 | Kaya 2018 | High |

*Table S4-181. Bicytopenia.*

|  | Study ID | Overall risk of bias |
| --- | --- | --- |
| 1 | Aypak 2015 | High |

*Table S4-182. Hemophagocytosis.*

|  | Study ID | Overall risk of bias |
| --- | --- | --- |
| 1 | Akbayram 2015 | High |
| 2 | Kaman 2022 | High |
| 3 | Demir 2021 | High |
| 4 | Citak 2010 | High |

*Table S4-183. Disseminated intravascular coagulation.*

|  | Study ID | Overall risk of bias |
| --- | --- | --- |
| 1 | Akdeniz 1998 | High |
| 2 | Al-Eissa 1993 | High |
| 3 | Al-Freihi 1986 | High |
| 4 | Khateeb 1990 | High |
| 5 | Mermut 2011 | High |
| 6 | Buzgan 2010 | High |

*Table S4-184. Myelodysplastic syndrome.*

|  | Study ID | Overall risk of bias |
| --- | --- | --- |
| 1 | Al Shaalan 2002 | High |

*Table S4-185. Cutaneous involvement.*

|  | Study ID | Overall risk of bias |
| --- | --- | --- |
| 1 | Akcali 2007 | Low |
| 2 | Al Hashan 2017 | High |
| 3 | Al Sawafi 2023 | High |
| 4 | Al Shamahy 2001 | High |
| 5 | Al-Dubooni 1986 | High |
| 6 | AlRashidi 2022 | High |
| 7 | Artuk 2019 | High |
| 8 | Aygen 2002 | High |
| 9 | Magableh 2007 | High |
| 10 | Behzadnia 2018 | High |
| 11 | Benjamin 1992 | High |
| 12 | Celen 2010 | High |
| 13 | Çirakli 2015 | High |
| 14 | Demirtuerk 2008 | High |
| 15 | El Bayoumy 2014 | High |
| 16 | Ertek 2006 | High |
| 17 | Fruchtman 2020 | High |
| 18 | Gottesman 1996 | High |
| 19 | Gul 2014 | High |
| 20 | Guler 2014 | High |
| 21 | Gür 2003 | High |
| 22 | Hasanjani Roushan 2016 a | High |
| 23 | Hizel 2007 | High |
| 24 | Kayaaslan 2016 | High |
| 25 | Kazak 2016 | High |
| 26 | Keyvanfar 2021 | High |
| 27 | Kokoglu 2006 | High |
| 28 | Kutlu 2018 | High |
| 29 | Ulug 2011 | High |
| 30 | Mermut 2011 | High |
| 31 | Mousa 1988 | High |
| 32 | Özen 2022 | High |
| 33 | Savion 2023 | High |
| 34 | Kayaaslan 2013 | High |
| 35 | Turunç 2008 | High |
| 36 | Naz 2009 | High |
| 37 | Al Shaalan 2002 | High |
| 38 | Qasim 2020 | High |
| 39 | Türker 2014 | High |
| 40 | Ertekin 2004 | High |
| 41 | Uluğ 2011 | High |
| 42 | Buzgan 2010 | High |
| 43 | Fanni 2013 | High |
| 44 | Keramat 2009 | High |
| 45 | Metin 2001 | High |
| 46 | Kaya 2018 | High |
| 47 | Najafi 2018 | High |
| 48 | Pourbagher 2006 | High |
| 49 | Vafaei 2019 | High |
| 50 | Shimol 2012 | High |
| 51 | Eskandari-Nasab 2014 | High |
| 52 | Kaygusuz 2005 | High |

*Table S4-186. Cellulitis.*

|  | Study ID | Overall risk of bias |
| --- | --- | --- |
| 1 | Hasanjani Roushan 2016 a | High |

*Table S4-187. Erythema Nodosum.*

|  | Study ID | Overall risk of bias |
| --- | --- | --- |
| 1 | Guler 2014 | High |
| 2 | Hadadi 2009 | High |
| 3 | Hasanjani Roushan 2004 | High |
| 4 | Buzgan 2010 | High |
| 5 | Metin 2001 | High |

*Table S4-188. Erythema Nodosum–like lesions.*

|  | Study ID | Overall risk of bias |
| --- | --- | --- |
| 1 | Akcali 2007 | Low |

*Table S4-189. Petechiae.*

|  | Study ID | Overall risk of bias |
| --- | --- | --- |
| 1 | Akdeniz 1998 | Low |
| 2 | Al-Eissa 1993 | High |
| 3 | Salman 2022 | High |
| 4 | Buzgan 2010 | High |

*Table S4-190. Petechiae and purpura.*

|  | Study ID | Overall risk of bias |
| --- | --- | --- |
| 1 | Afify 2013 | High |
| 2 | Çirakli 2015 | High |
| 3 | Çiğdem 2019 | High |
| 4 | El-Koumi 2013 | High |
| 5 | Buzgan 2010 | High |

*Table S4-191. Petechial rash.*

|  | Study ID | Overall risk of bias |
| --- | --- | --- |
| 1 | Keyvanfar 2021 | Low |
| 2 | Mermut 2011 | High |

*Table S4-192. Purpuric rash.*

|  | Study ID | Overall risk of bias |
| --- | --- | --- |
| 1 | Abdi-Liae 2007 | Low |
| 2 | Benjamin 1992 | High |

*Table S4-193. Maculopapular eruptions.*

|  | Study ID | Overall risk of bias |
| --- | --- | --- |
| 1 | Akcali 2007 | Low |
| 2 | AlRashidi 2022 | High |
| 3 | Artuk 2019 | High |
| 4 | Aygen 2002 | High |
| 5 | Gul 2014 | High |
| 6 | Guler 2014 | Low |
| 7 | Kayaaslan 2013 | High |
| 8 | Buzgan 2010 | High |

*Table S4-194. Psoriasiform lesions.*

|  | Study ID | Overall risk of bias |
| --- | --- | --- |
| 1 | Akcali 2007 | Low |
| 2 | Metin 2001 | High |

*Table S4-195. Malar eruption.*

|  | Study ID | Overall risk of bias |
| --- | --- | --- |
| 1 | Akcali 2007 | Low |
| 2 | Metin 2001 | High |

*Table S4-196. Palmar eczema.*

|  | Study ID | Overall risk of bias |
| --- | --- | --- |
| 1 | Akcali 2007 | Low |

*Table S4-197. Palmar erythema.*

|  | Study ID | Overall risk of bias |
| --- | --- | --- |
| 1 | Akcali 2007 | Low |
| 2 | Metin 2001 | High |

*Table S4-198. Ecchymosis.*

|  | Study ID | Overall risk of bias |
| --- | --- | --- |
| 1 | Parlak 2015 | High |

*Table S4-199. Diffuse rashes.*

|  | Study ID | Overall risk of bias |
| --- | --- | --- |
| 1 | Aypak 2012 | Low |

*Table S4-200. Pityriasis rosea.*

|  | Study ID | Overall risk of bias |
| --- | --- | --- |
| 1 | Guler 2014 | Low |

*Table S4-201. Gastrointestinal involvement.*

|  | Study ID | Overall risk of bias |
| --- | --- | --- |
| 1 | Artuk 2019 | High |
| 2 | Elhassanien 2014 | High |
| 3 | Ertek 2006 | High |
| 4 | Fallah 2005 | High |
| 5 | Fruchtman 2020 | High |
| 6 | Gul 2014 | High |
| 7 | Guler 2014 | High |
| 8 | Gür 2003 | High |
| 9 | Hasanjani Roushan 2016 a | High |
| 10 | Keyvanfar 2021 | High |
| 11 | Ulug 2011 | High |
| 12 | Mermut 2011 | High |
| 13 | Mousa 1988 | High |
| 14 | Naz 2009 | High |
| 15 | Uluğ 2011 | High |
| 16 | Sen 2019 | High |

*Table S4-202. Hepatobiliary system involvement.*

|  | Study ID | Overall risk of bias |
| --- | --- | --- |
| 1 | Guler 2014 | Low |
| 2 | Mermut 2011 | High |
| 3 | Ertem 2004 | High |

*Table S4-203. Hepatitis.*

|  | Study ID | Overall risk of bias |
| --- | --- | --- |
| 1 | Al Nokhatha 2021 | High |
| 2 | Artuk 2019 | High |
| 3 | Aygen 2002 | High |
| 4 | Çirakli 2015 | High |
| 5 | Demirtuerk 2008 | High |
| 6 | Ertek 2006 | High |
| 7 | Gul 2014 | High |
| 8 | Gür 2003 | Low |
| 9 | Kayaaslan 2016 | High |
| 10 | Kokoglu 2006 | High |
| 11 | Ulug 2011 | High |
| 12 | Namiduru 2003 | High |
| 13 | Tasbakan 2003 | High |
| 14 | Kayaaslan 2013 | High |
| 15 | Naz 2009 | High |
| 16 | Al Shaalan 2002 | High |
| 17 | Yoldas 2015 | High |
| 18 | Keramat 2018 | High |
| 19 | Kaya 2018 | High |
| 20 | Najafi 2018 | Low |

*Table S4-204. Clinical hepatitis.*

|  | Study ID | Overall risk of bias |
| --- | --- | --- |
| 1 | Khateeb 1990 | High |

*Table S4-205. Icteric hepatitis.*

|  | Study ID | Overall risk of bias |
| --- | --- | --- |
| 1 | Hasanjani Roushan 2004 | High |

*Table S4-206. Anicteric hepatitis.*

|  | Study ID | Overall risk of bias |
| --- | --- | --- |
| 1 | Khateeb 1990 | High |

*Table S4-207. Liver involvement.*

|  | Study ID | Overall risk of bias |
| --- | --- | --- |
| 1 | Dal 2013 | Low |
| 2 | Buzgan 2010 | High |
| 3 | Rezazadeh 2006 | High |

*Table S4-208. Liver cirrhosis.*

|  | Study ID | Overall risk of bias |
| --- | --- | --- |
| 1 | Shehabi 1990 | High |

*Table S4-209. Liver abscess.*

|  | Study ID | Overall risk of bias |
| --- | --- | --- |
| 1 | Ertek 2006 | High |
| 2 | Gür 2003 | High |
| 3 | Edathodu 2021 | High |
| 4 | Shehabi 1990 | High |

*Table S4-210. Ileitis.*

|  | Study ID | Overall risk of bias |
| --- | --- | --- |
| 1 | Gür 2003 | High |

*Table S4-211. Ascites.*

|  | Study ID | Overall risk of bias |
| --- | --- | --- |
| 1 | Bukharie 2009 | High |
| 2 | Gür 2003 | High |
| 3 | Kaya 2018 | High |
| 4 | Sen 2019 | High |

*Table S4-212. Cholecystitis*

|  | Study ID | Overall risk of bias |
| --- | --- | --- |
| 1 | El-Amin 2001 | High |
| 2 | Ertek 2006 | High |
| 3 | Keyvanfar 2021 | High |
| 4 | Mohamed 1986 | High |
| 5 | Naz 2009 | High |

*Table S4-213. Pancreatitis.*

|  | Study ID | Overall risk of bias |
| --- | --- | --- |
| 1 | Ertek 2006 | High |
| 2 | Keyvanfar 2021 | High |
| 3 | Suvak 2016 | High |
| 4 | Buzgan 2010 | High |

*Table S4-214. Necrotizing pancreatitis.*

|  | Study ID | Overall risk of bias |
| --- | --- | --- |
| 1 | Suvak 2016 | High |

*Table S4-215. Oropharyngeal involvement.*

|  | Study ID | Overall risk of bias |
| --- | --- | --- |
| 1 | Mousa 1988 | High |

*Table S4-216. Pharyngitis.*

|  | Study ID | Overall risk of bias |
| --- | --- | --- |
| 1 | Fallah 2005 | High |

*Table S4-217. Otolaryngology/head and neck involvement*

|  | Study ID | Overall risk of bias |
| --- | --- | --- |
| 1 | Lazutkin 2018 | High |

*Table S4-218. Appendicitis.*

|  | Study ID | Overall risk of bias |
| --- | --- | --- |
| 1 | Fallah 2005 | High |

*Table S4-219. Peritonitis.*

|  | Study ID | Overall risk of bias |
| --- | --- | --- |
| 1 | Aygen 2002 | High |
| 2 | Bukharie 2009 | High |
| 3 | El-Amin 2001 | High |
| 4 | Ertek 2006 | High |
| 5 | Fallah 2005 | High |
| 6 | Buzgan 2010 | High |

*Table S4-220. Respiratory system involvement.*

|  | Study ID | Overall risk of bias |
| --- | --- | --- |
| 1 | Bulut 2011 | High |
| 2 | Çirakli 2015 | High |
| 3 | Gür 2003 | Low |
| 4 | Hasanjani Roushan 2016 a | High |
| 5 | Hassan 2021 | High |
| 6 | Khateeb 1990 | High |
| 7 | Ulug 2011 | High |
| 8 | Mousa 1988 | Low |
| 9 | Naz 2009 | High |
| 10 | Uluğ 2011 | High |

*Table S4-221. Pulmonary involvement.*

|  | Study ID | Overall risk of bias |
| --- | --- | --- |
| 1 | Hatipoglu 2005 | High |
| 2 | Kokoglu 2006 | High |
| 3 | Namiduru 2003 | High |
| 4 | Simsek 2011 | High |
| 5 | Varikkodan 2024 | High |
| 6 | Türker 2014 | High |
| 7 | Torkaman 2017 | High |

*Table S4-222. Lung involvement.*

|  | Study ID | Overall risk of bias |
| --- | --- | --- |
| 1 | Kutlu 2018 | High |
| 2 | Lubani 1989 | High |

*Table S4-223. Lung abscess.*

|  | Study ID | Overall risk of bias |
| --- | --- | --- |
| 1 | Namiduru 2003 | High |
| 2 | Edathodu 2021 | High |

*Table S4-224. Pneumonia.*

|  | Study ID | Overall risk of bias |
| --- | --- | --- |
| 1 | Aygen 2002 | High |
| 2 | Benjamin 1992 | High |
| 3 | Erat 2023 | High |
| 4 | Fallah 2005 | High |
| 5 | Gonen 2014 | High |
| 6 | Gür 2003 | Low |
| 7 | Hatipoglu 2005 | High |
| 8 | Khateeb 1990 | High |
| 9 | Ulug 2011 | High |
| 10 | Mousa 1988 | Low |
| 11 | Zia Sheikholeslami 2007 | High |
| 12 | Al Shaalan 2002 | High |
| 13 | Türker 2014 | High |
| 14 | Yoldas 2015 | High |

*Table S4-225. Bronchitis.*

|  | Study ID | Overall risk of bias |
| --- | --- | --- |
| 1 | Gür 2003 | Low |
| 2 | Naz 2009 | High |

*Table S4-226. Pneumonitis or bronchiolitis.*

|  | Study ID | Overall risk of bias |
| --- | --- | --- |
| 1 | Kaya 2018 | High |

*Table S4-227. Pleural effusion.*

|  | Study ID | Overall risk of bias |
| --- | --- | --- |
| 1 | Celen 2010 | High |
| 2 | Gür 2003 | Low |
| 3 | Hatipoglu 2005 | High |
| 4 | Khateeb 1990 | High |
| 5 | Mousa 1988 | Low |
| 6 | Norton 1984 | High |
| 7 | Simsek 2011 | High |
| 8 | Shehabi 1990 | High |
| 9 | Lubani 1989 | High |
| 10 | Lifeso 1985 | High |

*Table S4-228. Pleurisy.*

|  | Study ID | Overall risk of bias |
| --- | --- | --- |
| 1 | Kokoglu 2006 | High |
| 2 | Namiduru 2003 | High |
| 3 | Buzgan 2010 | High |
| 4 | Kaya 2018 | High |

*Table S4-229. Pleural involvement.*

|  | Study ID | Overall risk of bias |
| --- | --- | --- |
| 1 | Tasbakan 2003 | High |

*Table S4-230. Emphysema.*

|  | Study ID | Overall risk of bias |
| --- | --- | --- |
| 1 | Simsek 2011 | High |

*Table S4-231. Pneumothorax.*

|  | Study ID | Overall risk of bias |
| --- | --- | --- |
| 1 | Patel 1988 | High |

*Table S4-232. Ocular involvement.*

|  | Study ID | Overall risk of bias |
| --- | --- | --- |
| 1 | Bulut 2011 | High |
| 2 | Fallah 2005 | High |
| 3 | Güngör 2002 | High |
| 4 | Gür 2003 | High |
| 5 | Mousa 1988 | High |
| 6 | Namiduru 2003 | High |
| 7 | Savas 2007 | High |
| 8 | Sungur 2009 | High |
| 9 | Karakurt 2023 | High |
| 10 | Sen 2019 | High |

*Table S4-233. Conjunctivitis.*

|  | Study ID | Overall risk of bias |
| --- | --- | --- |
| 1 | Güngör 2002 | High |
| 2 | Namiduru 2003 | High |
| 3 | Karakurt 2023 | High |

*Table S4-234. Panuveitis.*

|  | Study ID | Overall risk of bias |
| --- | --- | --- |
| 1 | Sungur 2009 | High |
| 2 | Karakurt 2023 | High |

*Table S4-235. Uveitis.*

|  | Study ID | Overall risk of bias |
| --- | --- | --- |
| 1 | Namiduru 2003 | High |

*Table S4-236. Anterior uveitis.*

|  | Study ID | Overall risk of bias |
| --- | --- | --- |
| 1 | Güngör 2002 | Low |
| 2 | Karakurt 2023 | High |

*Table S4-237. Posterior uveitis.*

|  | Study ID | Overall risk of bias |
| --- | --- | --- |
| 1 | Güngör 2002 | Low |

*Table S4-238. Dacryoadenitis.*

|  | Study ID | Overall risk of bias |
| --- | --- | --- |
| 1 | Güngör 2002 | Low |
| 2 | Namiduru 2003 | High |

*Table S4-239. Episcleritis.*

|  | Study ID | Overall risk of bias |
| --- | --- | --- |
| 1 | Güngör 2002 | Low |
| 2 | Namiduru 2003 | High |

*Table S4-240. Iridocyclitis.*

|  | Study ID | Overall risk of bias |
| --- | --- | --- |
| 1 | Savas 2007 | High |

*Table S4-241. Choroiditis.*

|  | Study ID | Overall risk of bias |
| --- | --- | --- |
| 1 | Güngör 2002 | Low |
| 2 | Sungur 2009 | High |
| 3 | Karakurt 2023 | High |

*Table S4-242. Cystoid macular edema.*

|  | Study ID | Overall risk of bias |
| --- | --- | --- |
| 1 | Sungur 2009 | High |

*Table S4-243. Retinitis.*

|  | Study ID | Overall risk of bias |
| --- | --- | --- |
| 1 | Buzgan 2010 | High |

*Table S4-244. Retinal haemorrhage.*

|  | Study ID | Overall risk of bias |
| --- | --- | --- |
| 1 | Karakurt 2023 | High |

*Table S4-245. Abortion.*

|  | Study ID | Overall risk of bias |
| --- | --- | --- |
| 1 | Elshamy 2008 | High |
| 2 | Inan 2019 | Low |
| 3 | Khateeb 1990 | High |
| 4 | Majzoobi 2023 | High |
| 5 | Mousa 1988 | High |
| 6 | Roushan 2011 | High |
| 7 | Gulsun 2011 | High |
| 8 | Kurdoglu 2010 | High |
| 9 | Khan 2001 | High |
| 10 | Buzgan 2010 | High |

*Table S4-246. Threatened abortion.*

|  | Study ID | Overall risk of bias |
| --- | --- | --- |
| 1 | Inan 2019 | Low |

*Table S4-247. Intrauterine fetal demise.*

|  | Study ID | Overall risk of bias |
| --- | --- | --- |
| 1 | Elshamy 2008 | High |
| 2 | Inan 2019 | Low |
| 3 | Majzoobi 2023 | High |
| 4 | Kurdoglu 2010 | High |
| 5 | Khan 2001 | High |

*Table S4-248. Preterm labour.*

|  | Study ID | Overall risk of bias |
| --- | --- | --- |
| 1 | Elshamy 2008 | High |
| 2 | Inan 2019 | Low |
| 3 | Majzoobi 2023 | High |
| 4 | Roushan 2011 | High |
| 5 | Gulsun 2011 | High |
| 6 | Kurdoglu 2010 | High |
| 7 | Khan 2001 | High |

*Table S4-249. Infant low birth weight.*

|  | Study ID | Overall risk of bias |
| --- | --- | --- |
| 1 | Inan 2019 | Low |
| 2 | Majzoobi 2023 | High |
| 3 | Gulsun 2011 | High |

*Table S4-250. Soft tissue involvement.*

|  | Study ID | Overall risk of bias |
| --- | --- | --- |
| 1 | Buzgan 2010 | High |

*Table S4-251. Thyroiditis.*

|  | Study ID | Overall risk of bias |
| --- | --- | --- |
| 1 | Buzgan 2010 | High |

*Table S4-252. Thyroid abscess.*

|  | Study ID | Overall risk of bias |
| --- | --- | --- |
| 1 | Lazutkin 2018 | High |

*Table S4-253. Visceral abscess.*

|  | Study ID | Overall risk of bias |
| --- | --- | --- |
| 1 | Kurtaran 2012 | High |

*Table S4-254. Local abscess.*

|  | Study ID | Overall risk of bias |
| --- | --- | --- |
| 1 | Keyvanfar 2021 | High |

*Table S4-255. Focal involvement.*

|  | Study ID | Overall risk of bias |
| --- | --- | --- |
| 1 | Dabaja-Younis 2023 | High |
| 2 | Dal 2013 | High |
| 3 | Kazak 2016 | High |
| 4 | Kursun 2013 | High |
| 5 | Özen 2022 | High |
| 6 | Varikkodan 2024 | High |
| 7 | Buzgan 2010 | High |
| 8 | Aktug-Demir 2014 | High |
| 9 | Najafi 2018 | High |
