# Supplementary material for: Frequency of Human Brucellosis Complications in West Asia: A Systematic Review and Meta‐Analysis
Source: Microbiologyopen. 2026 Sep 7;15(5):e70388. doi: 10.1002/mbo3.70388 (PMC13548425; doi:10.1002/mbo3.70388)
Supplement: Supplementary file 11 [file MBO3-15-e70388-s011.docx]

Figure S9-1. Musculoskeletal involvement.

Figure S9-2. Skeletal involvement.

Figure S9-3. Osteoarticular Involvement.

Figure S9-4. Sacroiliitis.

Figure S9-5. Sacroiliitis and sclerosis.

Figure S9-6. Spondylitis.

Figure S9-7. Spondylodiscitis.

Figure S9-8. Sacroiliitis and Spondylodiscitis.

Figure S9-9. Spondylodiscitis and arthritis.

Figure S9-10. Spondylitis and epiphysitis.

Figure S9-11. Spondylitis or spondylodiscitis.

Figure S9-12. Discitis.

Figure S9-13. Spinal brucellosis.

Figure S9-14. Spinal Cord Involvement (vertebral osteomyelitis).

Figure S9-15. Arthritis or peripheral arthritis.

Figure S9-16. Sternoclavicular arthritis.

Figure S9-17. Arthropathy.

Figure S9-18. Osteomyelitis.

Figure S9-19. Joint effusion.

Figure S9-20. Epidural abscess.

Figure S9-21. Vertebral abscess.

Figure S9-22. Paravertebral abscess.

Figure S9-23. Paravertebral or epidural abscess.

Figure S9-24. Periarticular soft tissue involvement.

Figure S9-25. Bursitis.

Figure S9-26. Tendinitis.

Figure S9-27. Synovitis.

Figure S9-28. Avascular necrosis of femoral head.

Figure S9-29. Genito-urinary system involvement.

Figure S9-30. Urinary system involvement.

Figure S9-31. Orchitis.

Figure S9-32. Epididymitis.

Figure S9-33. Epididymoorchitis.

Figure S9-34. Testis hydrocele.

Figure S9-35. Testicular abscess.

Figure S9-36. Testicular edema.

Figure S9-37. Prostatitis.

Figure S9-38. Nephritis.

Figure S9-39. Renal failure.

Figure S9-40. Lymphadenopathy.

Figure S9-41. Lymphadenitis.

Figure S9-42. Lymphadenomegaly.

Figure S9-43. Adenopathy.

Figure S9-44. Parotitis.

Figure S9-45. Spleen abscess.

Figure S9-46. Nervous system involvement.

Figure S9-47. Central nervous system involvement.

Figure S9-48. Peripheral nervous system involvement.

Figure S9-49. Neurobrucellosis.

Figure S9-50. Neuropsychiatric involvement.

Figure S9-51. Psychiatric involvement.

Figure S9-52. Insomnia.

Figure S9-53. Sleep disturbance.

Figure S9-54. Meningitis.

Figure S9-55. Meningoencephalitis.

Figure S9-56. Meningitis or meningoencephalitis.

Figure S9-57. Meningeal irritation findings.

Figure S9-58. Meningovascular involvement.

Figure S9-59. Encephalitis.

Figure S9-60. Myelitis.

Figure S9-61. Intra-cerebral haemorrhage.

Figure S9-62. Subdural hematoma.

Figure S9-63. Sub-arachnoid haemorrhage.

Figure S9-64. Polyneuropathy.

Figure S9-65. Radiculopathy.

Figure S9-66. Polyradiculopathy.

Figure S9-67. Polyradiculoneuritis.

Figure S9-68. Peripheral neuritis.

Figure S9-69. Peripheral neuropathy.

Figure S9-70. Brain atrophy.

Figure S9-71. Hydrocephalus.

Figure S9-72. Brain abscess.

Figure S9-73. Cranial nerve involvement.

Figure S9-74. Cranial nerve palsy.

Figure S9-75. Nervus Abducens involvement.

Figure S9-76. Vestibulocochlear nerve involvement.

Figure S9-77. Facial paralysis.

Figure S9-78. Bell’s palsy.

Figure S9-79. Hearing loss.

Figure S9-80. Tinnitus.

Figure S9-81. Papilledema.

Figure S9-82. Paralysis.

Figure S9-83. Myelopathy.

Figure S9-84. Ataxia.

Figure S9-85. Depression.

Figure S9-86. Status epilepticus.

Figure S9-87. Neurological disorder.

Figure S9-88. Cardiovascular system involvement.

Figure S9-89. Cardiac involvement.

Figure S9-90. Carditis.

Figure S9-91. Endocarditis.

Figure S9-92. Myocarditis.

Figure S9-93. Pericarditis.

Figure S9-94. Thrombophlebitis.

Figure S9-95. Deep vein thrombosis.

Figure S9-96. Capillary leak syndrome.

Figure S9-97. Hematologic involvement.

Figure S9-98. Leukopenia.

Figure S9-99. Leukocytosis.

Figure S9-100. Lymphopenia.

Figure S9-101. Lymphocytosis.

Figure S9-102. Neutropenia.

Figure S9-103. Neutrophilia.

Figure S9-104. Eosinophilia.

Figure S9-105. Lymphomonocytosis.

Figure S9-106. Monocytosis.

Figure S9-107. Anemia.

Figure S9-108. Thrombocytopenia.

Figure S9-109. Thrombocytosis.

Figure S9-110. Pancytopenia.

Figure S9-111. Cytopenia.

Figure S9-112. Hemophagocytosis.

Figure S9-113. Disseminated intravascular coagulation.

Figure S9-114. Cutaneous involvement.

Figure S9-115. Erythema Nodosum.

Figure S9-116. Petechiae.

Figure S9-117. Petechiae and purpura.

Figure S9-118. Petechial rash.

Figure S9-119. Purpuric rash.

Figure S9-120. Maculopapular eruptions.

Figure S9-121. Psoriasiform lesions.

Figure S9-122. Malar eruption.

Figure S9-123. Palmar erythema.

Figure S9-124. Gastrointestinal involvement.

Figure S9-125. Hepatobiliary system involvement.

Figure S9-126. Hepatitis.

Figure S9-127. Liver involvement.

Figure S9-128. Liver abscess.

Figure S9-129. Ascites.

Figure S9-130. Cholecystitis.

Figure S9-131. Pancreatitis.

Figure S9-132. Peritonitis.

Figure S9-133. Respiratory system involvement.

Figure S9-134. Pulmonary involvement.

Figure S9-135. Lung involvement.

Figure S9-136. Lung abscess.

Figure S9-137. Pneumonia.

Figure S9-138. Bronchitis.

Figure S9-139. Pleural effusion.

Figure S9-140. Pleurisy.

Figure S9-141. Ocular involvement.

Figure S9-142. Conjunctivitis.

Figure S9-143. Panuveitis.

Figure S9-144. Anterior uveitis.

Figure S9-145. Dacryoadenitis.

Figure S9-146. Episcleritis.

Figure S9-147. Choroiditis.

Figure S9-148. Abortion.

Figure S9-149. Intrauterine fetal demise.

Figure S9-150. Preterm labour.

Figure S9-151. Infant low birth weight.

Figure S9-152. Focal involvement.
